# Information extraction from free text for aiding transdiagnostic psychiatry: constructing NLP pipelines tailored to clinicians’ needs

**DOI:** 10.1186/s12888-022-04058-z

**Published:** 2022-06-17

**Authors:** Rosanne J. Turner, Femke Coenen, Femke Roelofs, Karin Hagoort, Aki Härmä, Peter D. Grünwald, Fleur P. Velders, Floortje E. Scheepers

**Affiliations:** 1 University Medical Center Utrecht, Brain Center, Amsterdam, Netherlands; 2 Machine Learning Group, CWI, Amsterdam, Netherlands; 3 Philips Research, Eindhoven, Netherlands; 4 Department of Mathematics, Leiden University, Leiden, Netherlands

**Keywords:** Transdiagnostic psychiatry, Natural language processing, Machine learning, Depression, Hamilton, SF-36

## Abstract

**Background:**

Developing predictive models for precision psychiatry is challenging because of unavailability of the necessary data: extracting useful information from existing electronic health record (EHR) data is not straightforward, and available clinical trial datasets are often not representative for heterogeneous patient groups. The aim of this study was constructing a natural language processing (NLP) pipeline that extracts variables for building predictive models from EHRs. We specifically tailor the pipeline for extracting information on outcomes of psychiatry treatment trajectories, applicable throughout the entire spectrum of mental health disorders (“transdiagnostic”).

**Methods:**

A qualitative study into beliefs of clinical staff on measuring treatment outcomes was conducted to construct a candidate list of variables to extract from the EHR. To investigate if the proposed variables are suitable for measuring treatment effects, resulting themes were compared to transdiagnostic outcome measures currently used in psychiatry research and compared to the HDRS (as a gold standard) through systematic review, resulting in an ideal set of variables. To extract these from EHR data, a semi-rule based NLP pipeline was constructed and tailored to the candidate variables using Prodigy. Classification accuracy and F1-scores were calculated and pipeline output was compared to HDRS scores using clinical notes from patients admitted in 2019 and 2020.

**Results:**

Analysis of 34 questionnaires answered by clinical staff resulted in four themes defining treatment outcomes: symptom reduction, general well-being, social functioning and personalization. Systematic review revealed 242 different transdiagnostic outcome measures, with the 36-item Short-Form Survey for quality of life (SF36) being used most consistently, showing substantial overlap with the themes from the qualitative study. Comparing SF36 to HDRS scores in 26 studies revealed moderate to good correlations (0.62—0.79) and good positive predictive values (0.75—0.88). The NLP pipeline developed with notes from 22,170 patients reached an accuracy of 95 to 99 percent (F1 scores: 0.38 – 0.86) on detecting these themes, evaluated on data from 361 patients.

**Conclusions:**

The NLP pipeline developed in this study extracts outcome measures from the EHR that cater specifically to the needs of clinical staff and align with outcome measures used to detect treatment effects in clinical trials.

**Supplementary Information:**

The online version contains supplementary material available at 10.1186/s12888-022-04058-z.

## Background

In psychiatry, it is still difficult to choose the best treatment for individual patients based on their specific characteristics. For example, in major depressive disorder, only one third of patients achieves remission after first-line treatment [1]. This is why there is a plethora of attempts at developing machine learning models that support shared decision making and precision psychiatry (for example see Ermers et al. [2] for a recent overview of machine learning models in major depressive disorder, and Sanfelici et al. [3] for psychosis). However, as patient needs are personal and treatment outcomes are never binary in psychiatry [4], choosing a representative outcome measure on which the machine learning models should report is key, but not straightforward.

In clinical trials, diagnosis-specific symptom rating scales are frequently used to detect treatment effects. However, these measures restrict developing decision support models to just one group of patients with the same “diagnostic label”, whereas in practice, there almost never is a one-to-one correspondence between diagnostic labels and patients [5]. In addition, availability of patients’ scores on rating scales in the electronic health records (EHR) is limited in practice, as they are mostly registered structurally in the clinical trial setting. Lastly and perhaps most importantly, symptom rating scales may not cover all information patients and clinicians are actually interested in with regard to recovery, for example insights into daily and social functioning.

Hence, alternative outcome measures for machine learning models to support patients and clinicians in (shared) decision making seem warranted. One alternative could be using scores that represent the patient's functioning, as they can be used to follow up treatment effectiveness in patients with different psychiatric disorders. This way, predictive models in which patients from a wide spectrum of mental disorders are included could utilize these outcome measures. Functional outcome measures may also better reflect added value for patients and the community [6], making machine learning models’ predictions more insightful in comparison to predicting improvements on symptom rating scales.

This kind of information is not registered in a structured manner in the EHR, and extracting such outcome variables from clinical free text is a time-consuming process. On the other hand, it is unwarranted to introduce new questionnaires to clinical staff to collect data prospectively in a structured format for each predictive model that is built, as this would disproportionally increase administrative burden. Therefore, the aim of this study was to build a natural language processing (NLP) pipeline that can easily be tailored towards extracting specific information from clinical notes, and to show a specific application for extracting transdiagnostic outcome measures for mental health disorders.

To investigate which information would be valuable to report on in psychiatric clinical practice, psychiatry clinical staff of an academic hospital answered questionnaires to assess which outcome measures they would find appropriate to determine the effectiveness of treatment throughout the entire spectrum of mental health disorders. So far, most predictive models in psychiatry have been built around diagnosis-specific outcome measures [7], hence it is currently unknown whether treatment effects could be reflected adequately through more transdiagnostic and functional outcome measures, and whether it would be sensible to construct predictive models for these outcome measures at all. Therefore, to assess which transdiagnostic outcome measures resulting from the questionnaires were candidates, an overview of transdiagnostic measures used for detecting treatment effects in the research setting was created through systematic review. Second, the aptness of the found transdiagnostic measures for measuring treatment effects was assessed through comparing transdiagnostic domain scores in depression clinical trials with the gold standard in depression, the Hamilton Depression Rating Scale (HDRS), also through systematic review [8, 9].

The results of the questionnaires and systematic reviews were combined into a list of candidate transdiagnostic outcome measures. Finally, it was assessed whether these could be accurately extracted from the EHR data with our proposed NLP pipeline. To compare the extracted outcomes to a gold standard measure in a subgroup of patients with symptoms of depression, analogously to the comparison of the outcome measures and HDRS through the systematic review, the association between the outcome measures constructed with the NLP pipeline and HDRS scores of patients at the academic hospital was assessed.

## Methods

### Determining which information on treatment outcomes is valuable in clinical practice

To investigate which transdiagnostic outcome measures contain useful information for clinical practice, online questionnaires were developed and distributed among clinical staff at the Psychiatry department of UMCU (through Castor EDC, Ciwit B.V.). Questionnaires contained a combination of seven closed and seven open questions on defining recovery and treatment goals relevant for clinical decision making. For the analysis of the open questions, the framework for thematic analysis by Braun and Clarke was used [10]. Detailed methods can be found in the additional information file, Sect. 2.

### Identifying transdiagnostic outcome measures used in research

To further assess which outcome measures would be potential candidate measures for measuring treatment effects throughout the entire spectrum of mental health disorders, we aimed to find all transdiagnostic outcome measures that have been used in clinical trials from 2015 up to July 2020 through systematic review. The six-year cutoff was chosen to be able to focus on currently relevant outcome measures applicable to the Diagnostic and Statistical Manual of Mental Disorders, fifth Edition [11]. Studies concerning adult patients primarily diagnosed with a psychiatric disorder where at least one transdiagnostic outcome measure was used were included (details in additional information, Sect. 3).

### Assessing transdiagnostic outcomes for measuring treatment effects

In the second review, the aptness of a transdiagnostic outcome measure to measure treatment effects was investigated through comparing changes in the 36-item Short-Form Survey for quality of life (SF36) with the gold standard in depression, the HDRS. All clinical trials up to July 2020 concerning patients with depression where both the HDRS and the SF36 were utilized as primary or secondary outcome measures were included. Mean SF36 subcomponent score changes were compared to the mean HDRS score changes through weighted correlation, and a confusion matrix was created to investigate the ability of the SF36 to reveal a significant treatment effect (details in additional information, Sect. 4).

### Assessing routinely collected information in the EHR as information sources

To find sources to extract information on candidate themes after systematic review and qualitative analysis, the full spectrum of EHR data available at the psychiatry department of UMCU until 2020 was assessed, which included data from 22,170 patients: de-identified doctors’ and nurses’ notes [12], referral and dismissal letters, standardized forms containing treatment and prevention plans, standardized questionnaires performed (semi-)structurally, juridical status, destination after dismissal, lab measurements and prescribed medication. These sources were qualitatively assessed with regard to frequency of availability, relevance and quality.

### Constructing an NLP pipeline

To extract outcome measures from the unstructured data sources, the doctors’ and nurses’ notes, an NLP pipeline for analyzing Dutch clinical notes was developed, using as many available clinical text as possible, including notes from 5664 inpatient trajectories and from 18,689 patients that were treated ambulatory. The main aim of the pipeline was to find for each patient all sentences that contain clinically relevant information about the candidate themes resulting from the qualitative study and reviews, and to attach a sentiment score for each theme to the sentences to be able to see if observations were positive or negative. As there is often a lot of repetition in daily written clinical notes (e.g., “Situation has not changed, patient still lacks initiative and still has a depressed mood”), we aimed to let the pipeline only filter and score sentences that contained an indicator of change in the patient’s situation. This would probably give clinicians information that is more relevant to the course of treatment, compared to including sentences without change indicators in the scores.

**Fig. 1:**
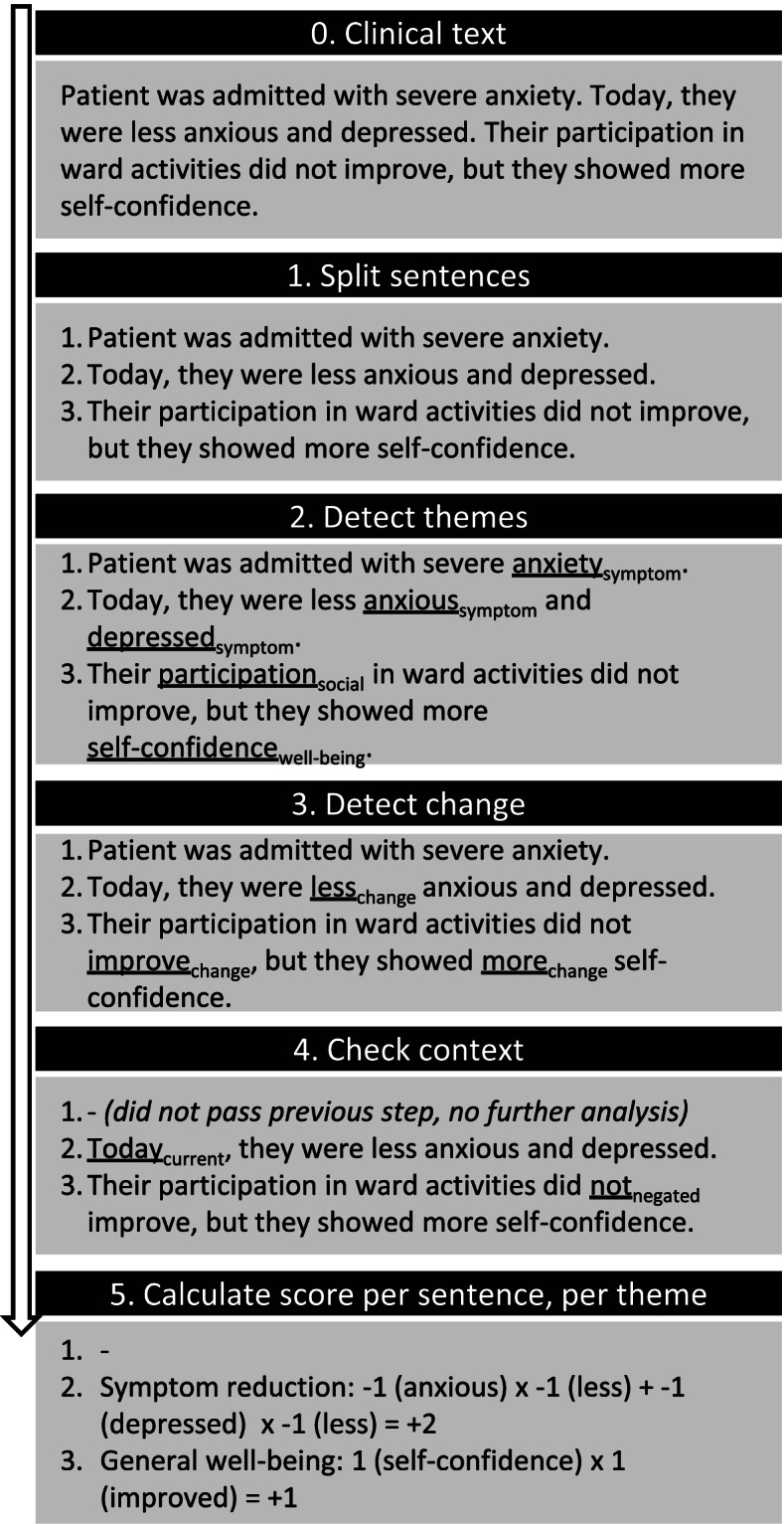
Schematic depiction of the NLP pipeline for extracting moments of change for each patient from clinical notes with a hypothetical example of a clinical text passing through all steps. Note that because in step 3 no change word was detected in sentence 1, further analysis of that sentence is cancelled. Note also that in step 4, a negated context is detected for the word “improve” in sentence 3, hence this change word and the corresponding theme word are not passed further through the analysis

A schematic overview of the proposed NLP pipeline with a hypothetical example of the analysis of a piece of clinical text can be found in Fig. 1. The five steps of analysis are briefly described in the next two paragraphs. Main units of analysis in the pipeline are sentences: in the first step, clinical notes are preprocessed by splitting them into sentences with a spaCy tokenizer [13]. In the second step, the sentences pass the theme filter, passing only when at least one phrase corresponding to one of the candidate themes is detected. In the third step, sentences pass through the change filter when they contain a phrase indicating a moment of change. This could either be a word directly describing change (e.g. “improvement”), or a comparative form of an adjective (e.g. “angrier”). For the theme and change filters, lists of phrases for rule-based filtering (in Dutch) were needed. These were composed with the annotation tool Prodigy by authors RJT and FC (Prodigy, ExplosionAI, Berlin, Germany). Prodigy takes as input a spaCy model and a list of seed terms, and based on these seed terms and the word embeddings in the model efficiently suggests new phrases to add to the list. One of the major advantages of this method for composing a phrase list is that frequent spelling errors are included. Examples of parts of the composed lists with translations to English can be found in additional Table 1, and complete composed phrase lists (in Dutch) can be found in the online repository for this project, available on GitHub [14].

In the fourth step, a context filter was applied to check if the theme phrase and change phrase were mentioned in a correct sentence context. Five checks were performed: whether the phrases were current, not hypothetical, concerned the patient, not negated, and whether the change concerned the theme (e.g., we need to detect “Today, anxiety symptoms increased”, but not “We increased the medication doses but the patient’s anxiety did not respond”). This filter uses part-of-speech and dependency tagging based on a previously developed spaCy model, regular expressions and literal phrases; details and a tutorial of the software can be found in [15] and [16]. In the fifth and final step, the sentences received scores for all themes that passed the filter for that specific sentence. This was done by RJT and FC through assigning sentiment scores to the theme and change phrases. For this project, we chose to assign negative phrases (e.g. “anxiety”, “anger”) the value -1, and positive phrases (e.g., “joy”, “hygiene”) the value 1. Change words indicating an increase were assigned the value 1, and those indicating a decrease the value -1. Final sentiment scores per sentence were calculated by multiplying each theme phrase score with its corresponding change phrase score. This way, an increase in something with a negative connotation, such as “more anxiety”, would result in a score of -1, and an increase in something with a positive connotation, such as “participation improved”, would result in a score of + 1 (see also Fig. 1). When a sentence contained multiple theme phrases with a corresponding change phrase, e.g., “The patient was more anxious and sad”, the scores were added, this example sentence resulting in a score of -2.

To assess if this pipeline could accurately extract sentences containing a moment of change with respect to the themes (regardless of sentiment), four validation datasets, one for each theme, were composed efficiently with the use of spaCy and Prodigy. As validation data, clinical notes from adult patients with one or more inpatient treatment trajectories at UMCU in 2020 were used, which were unseen during the phrase list development process described above. Using the theme phrases as a warm start, Prodigy selected sentences from the total data pool to label based on classification difficulty. Sentences were then labelled manually by RJT and FC, labelling a sentence as “accept” when it was judged that it should pass through all filters, and “reject” when it was judged to not contain a moment of change concerning the theme, in the correct contexts. The pipeline was also applied to this validation set, also labelling a sentence as “accept” when it passed through all filters for that specific theme, and “reject” when it did not pass. Given and predicted labels were then compared, and classification accuracy, precision, recall and F1-scores were calculated.

### Comparison of our candidate transdiagnostic outcomes to a gold standard

Finally, to compare our structured and unstructured transdiagnostic outcome measures to a more symptom-specific gold standard, the NLP theme scores and scores on domains extracted from structured sources (e.g., juridical status and medication prescriptions) were compared to HDRS scores for patients admitted in 2019 and 2020 through linear regression with stepwise AIC-based model selection in R. Summary scores for each patient were obtained by calculating the mean sentiment over all sentences that passed the filters for that patient for each theme. E.g., if for the theme “symptoms” three sentences passed the filter for a patient, with scores -2, 1 and 2, the mean symptom sentiment score for this patient would be 1/3. Only complete cases, with clinical notes and information from all selected structured sources available, were analyzed. The scores from structured sources were incorporated as categorical data, either having worsened (e.g. more benzodiazepine prescriptions at the end of an admission compared to the start), having stayed the same or having improved. In potential, such a linear model trained to reflect gold standard HDRS scores could be used to in the end compose a combined weighted score from the NLP scores and information from structured data.

For all analyses, R (version 4.0.3) and Python (version 3.7.4) were used.

## Results

### Clinicians' views on outcomes

Between June 23, 2020, and July 27, 2020, 38 healthcare professionals gave consent to participate in a survey on defining goals of treatment and recovery. 34 completed at least one item of the questionnaire. The group comprised 12 nurses, 3 nurse practitioners, 9 residents in psychiatry, and 10 psychiatrists. Through qualitative analysis, four distinct themes were identified that comprise the concepts “goals of treatment” and “recovery of a patient”: personalization, symptom reduction, general well-being and social functioning. Detailed descriptions of the themes are depicted in Table 1.

**Table 1:** Qualitative analysis of clinical staff’s responses to a questionnaire on defining goals of treatment and recovery

Theme	Description	Examples
Personalization	Recovery is a highly personal process that is shaped by the patient’s goals, story and views. Therefore, the treatment goals are dependent on the needs and goals of the patient. A situation is pursued in which professional care is no longer needed and the patient returns to his usual environment and position before illness	“The patient’s request, what he/she requires to function to his/her own needs…” “In this respect it is always necessary to look at the patient’s position before his illness, what he/she aims to accomplish, and which other factors are hindering, respectively facilitating the patient.”
Symptom reduction	Treatment goals include reduction of symptoms, encompassing both psychiatric and somatic complaints. This reduction ranges from complete remission to mere stabilization in the acute phase of the illness. The recovery process is hard work and sometimes involves an initial aggravation (e.g., side effects). The aim is that the symptoms are diminished in a way that the patient is not restricted by them anymore (e.g., in daily functioning), or that the patient can function on his previous level again	“Supporting patients in their recovery by treatment of psychiatric illness or symptoms.” “Reduction or recovery of symptoms.” “…as symptom-free as possible…” “Recovery to the level of premorbid functioning and reduction of symptoms to premorbid”
General well-being	Another treatment goal is to raise general well-being and quality of life. The treatment stimulates that the patient gains insight into his illness and learns to cope with it and the vulnerability that remains when the symptoms are reduced. A new balance is established between the patient’s capacities and the burden of the illness. This gives room for positive experiences, joy and a regained purpose in life	“Improvement of quality of life.” “Feeling like living and being able to experience life satisfaction again.” “Regaining a purpose and a balance between the patient’s capacities and the burden of the illness.”
Social functioning	Finally, treatment aims to improve the patient' social and societal functioning. The healthcare professionals try to enhance autonomy and self-sufficiency, so that the patient becomes able to participate in society again. This entails e.g., living independently, engaging in activities that are important to the patient, having a job and meaningful relationships with others	“Treatment of complaints, that give severe hinder in daily life, of the patient so that the patient is able to gradually resume his/her life and participate in society again.” “Recovery of healthy functioning on life domains like work, relationships, living and spare time.”

### Transdiagnostic outcome measures in research

The search for clinical trials where transdiagnostic outcomes were used yielded 1962 studies, of which 362 were included (details of exclusion criteria and an overview of included studies can be found in additional information, Sect. 3 and additional Table 2). In these studies, 242 different transdiagnostic outcome measures were applied. The most prevalent outcome measures were the Clinical Global Impression (CGI), Short Form Health Survey (SF), Global Assessment of Functioning (GAF), EuroQol 5d (EQ-5D) and World Health Organization Quality of Life (WHOQOL) questionnaires [17–21]. An overview of the ten most-used outcome measures is provided in the additional information, additional Table 3. The CGI and the GAF concern very short surveys, but the SF, EQ-5D and WHOQOL all three concern longer, detailed questionnaires with overlapping themes concerning physical, mental and emotional well-being, and social and societal functioning.

**Fig. 2:**
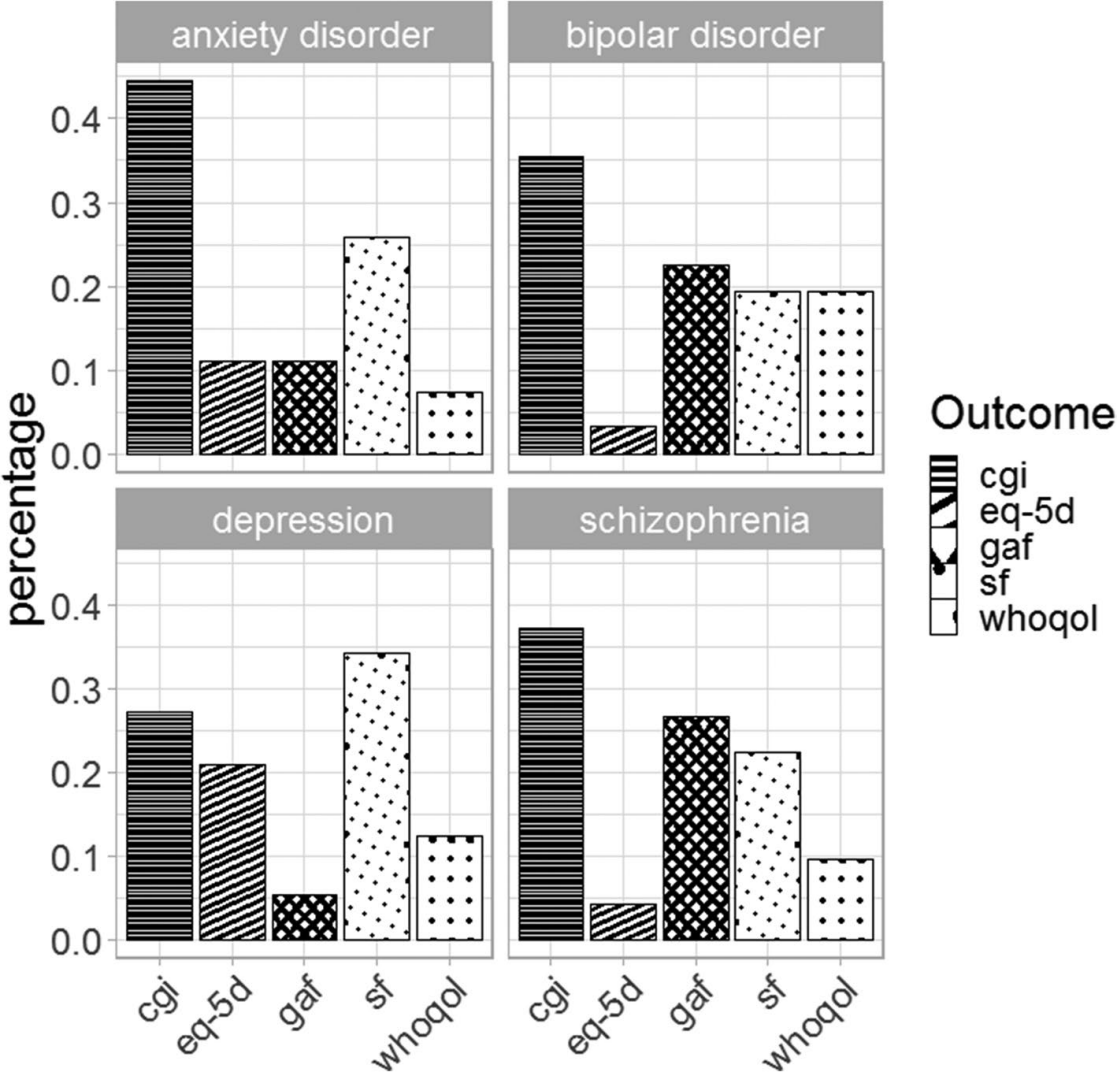
Top 5 most-used transdiagnostic outcome measures during the past five years. The prevalence of usage of the top 5 most-used transdiagnostic outcome measures for the most prevalent diagnoses for which general outcome measures were used during the past 5 years in clinical trials are shown

In Fig. 2 the frequency of usage of these outcome measures per diagnosis is illustrated. The SF is used in a substantial portion of studies for all diagnoses, whereas for the other questionnaires the usage varies depending on the specific diagnosis. The 36-item, most widely used version of the SF (SF36) consists of eight subcomponents; physical health, physical role perception, bodily pain, general health perception, mental health, emotional role perception, vitality and social functioning, which together roughly cover the spectrum of topics covered by the other most-used questionnaires. As the SF36 also is the most widely-used method to quantify health-related quality of life [22], these SF36 subcomponents were used for further investigation of the extent to which a transdiagnostic outcome measure is as sensitive to changes over the course of treatment compared with diagnosis-specific questionnaires.

### Transdiagnostic outcomes for measuring treatment effects

Systematic review yielded 26 studies where both SF36 and HDRS were measured during treatment trajectories of patients with depression; detailed results can be found in the additional information, additional table 4. The strength of the Pearson correlation coefficients between SF36 subscores and changes in HDRS scores varied from moderate to strong with bodily pain to be the lowest, and physical health perception to be the highest (R = -0.601, and R = -0.786, respectively), i.e., better scores on the subcomponents of the SF36 indicate an improvement of symptoms of depression (additional table 5). The positive predictive value of most of the SF36 subscores was high, indicating that the SF36 is apt for detecting treatment effects (additional table 6).

Interestingly, the themes resulting from the interviews with psychiatry staff show substantial overlap with the subcomponents of the ten most prevalent questionnaires found in our systematic reviews, which also mainly focused on (physical and) mental health symptoms, social and societal functioning and more general emotional well-being. Specifically comparing them to the SF36, the theme “symptom reduction” corresponds to the subcomponents physical health, bodily pain and mental health, the theme “social functioning” directly to its social functioning counterpart, “general well-being” to vitality and general health perception and “personalization” to physical role perception, general health perception and emotional role perception. With the SF36 subcomponents deemed as good alternatives for measuring treatment effects compared to a syndrome-specific gold standard in the systematic review above, it was hypothesized that these four themes would be good candidate treatment effect measures as well, while also catering to the specific needs of clinicians in psychiatry practice.

### Extraction from the EHR

To report on outcomes on the four found themes for individual patient treatment trajectories, all EHR sources available at the psychiatry department of UMCU until 2020 (22,170 patients) were assessed with regard to availability of information on the themes, and for relevance and quality of this information. An overview of assessed sources and their aptness is given in the additional information, additional table 7. For the information extraction, the definition of the “personalization” theme was narrowed down to “patient experience”, and for this theme, the EHR sources were searched for information on the thoughts and remarks of patients about their treatment trajectory. Sources selected as feasible for calculating theme scores were clinical notes (for each theme), juridical status (for symptoms and social functioning), medication prescriptions during admission (for symptoms) and destination after dismissal (for symptoms and social functioning).

### NLP pipeline assessment

To validate the NLP pipeline for extracting information from unstructured EHR sources, validation sets were composed for each theme with all clinical notes of admitted, adult patients at the UMCU in 2020. This set comprised 439 trajectories of 361 patients with a mean duration of 57 days; 39 percent was admitted to emergency care, 31 percent to a ward specialized in the diagnosis of first episode psychosis and 26 percent to a ward specialized in affective and psychotic disorders. In Table 2, the average number of sentences containing a phrase for each of the four themes per inpatient treatment trajectory, the number of sentences selected by the pipeline as mentioning a change in the theme in the correct context and some example sentences can be found. On validation sets with 663, 292, 328 and 269 sentences for symptoms, social, well-being and patients’ experience, respectively, 0/1 accuracies between 95 and 99 percent were achieved on each of the themes (also see Table 2). Remarkable is the high precision, but low recall for the symptom reduction and general well-being themes; reviewing the false negative sentences revealed that a large part could be contributed to missed verb conjugations in the change phrases, and specifically conjugation breaks, which occur a lot in Dutch. Also notable is the low precision but high recall for the patient experience theme.

**Table 2:** Results of the NLP pipeline applied to all clinical notes of 2020

Theme	Mean number of sentences concerning theme per trajectory (sd)	Mean number of sentences with relevant change in theme per trajectory (sd)	Examples of sentences translated from Dutch marked as correct	Classification accuracy of pipeline	Precision	Recall	F1-score
Symptom reduction	103 (99.8)	8.0(10.2)	“Nervousness increased over the course of the day”, “The patient appears drowsier than before”	0.988	0.857	0.461	0.6
Social functioning	131 (121)	4.0 (5.1)	“Friendly, more interaction than yesterday”	0.997	0.750	1.00	0.857
General well-being	119 (126)	6.4 (8.9)	“This afternoon, the patient felt less well”, “Had less energy”	0.951	0.833	0.250	0.385
Patient experience	164 (159)	9.4 (11.0)	“Says that it is going well, has the idea that it is going better and better”	0.993	0.333	1.00	0.5

As an example of clinical applicability, to assess the aptness of these scores, in addition to the scores from the structured sources (medication prescriptions, juridical status and destination after dismissal) to reflect treatment effects during an inpatient treatment trajectory, they were compared to changes in HDRS scores in patients with symptoms of depression. These were available for 120 patients in 2019 and 2020; 80 of these patients were admitted to the ward for affective and psychotic disorders, and 40 to other wards. The mean HDRS score at the end of inpatient treatment trajectories was 14, with a minimum of 2 and a maximum of 33. On average, 2802 sentences of clinical notes were available for each patient, and 88 change sentences passed the filter. Linear regression with stepwise model selection revealed that the most parsimonious model (based on AIC) for predicting HDRS scores at the end of inpatient treatment trajectories included mean sentiment scores for the symptom and social functioning themes, juridical status, benzodiazepine prescriptions and other psychiatric medication prescriptions as covariates (Table 3). Negative model coefficients were found for the sentiment of psychiatric core symptoms and a decrease (i.e. improvement) in benzodiazepine prescriptions, implying that improvements on these themes are associated with improvement of depression symptoms.

**Table 3:** The most parsimonious linear regression model after stepwise model selection with AIC for predicting HDRS scores at the end of treatment trajectories

Predictor	Coefficient	Standard error	*P*-value
*(Intercept)*	*8.549*	*3.151*	*0.00777*
Mean sentiment psychiatry symptoms	-3.711	1.336	0.00645
Mean sentiment social functioning	2.354	1.318	0.07692
No change in juridical status	5.496	0.920	0.35966
Juridical status improved	5.412	2.698	0.04739
No change in benzodiazepine prescriptions	-3.769	1.774	0.03589
Decrease in benzodiazepine prescriptions	-3.467	2.288	0.13264
No change in other psychiatry medication prescriptions	2.346	1.628	0.15243
Decrease in other psychiatry medication prescriptions	3.403	1.881	0.07315

## Discussion

With the research described in this paper, we aimed to identify useful and real-time extractable outcome measures for machine learning models in psychiatry. Through systematic review, transdiagnostic outcome measures concerning core symptoms, social functioning and general well-being were identified. Comparison of scores on these themes with Hamilton scores through systematic review showed that these themes appropriately reflect outcomes of treatment trajectories. Themes defined by clinicians at the academic hospital that together cover the spectrum of defining successful treatment trajectories were symptom reduction, general well-being, social functioning and personalization, which show substantial overlap with the themes found through systematic review. Through combining structured and unstructured EHR data that was already available, an NLP pipeline was developed through which scores on the subthemes could be extracted from the EHR, with good F1-scores for detecting information on symptoms and social functioning.. The symptom reduction and social functioning themes were associated with HDRS scores for patients admitted in 2019 and 2020.

In this study we composed the phrase lists for text mining each theme ourselves, tailored to the current specific problem and clinician writing styles, which required a substantial time investment. In future research the performance of our phrase lists could be compared with existing medical ontologies like Systemized Nomenclature of Medicine Clinical Terminology (SNOMED-CT) [23]. However, existing medical ontologies do not address issues like spelling mistakes and form variability, which might decrease their sensitivity. Similarly, the rule-based nature of our pipeline did not allow for enough flexibility to cover all verb conjugations in Dutch, possibly explaining the low recall on the symptom and well-being themes. The pipeline also had a low precision for the patient experience theme, probably also explained by the rule-based nature; because of the broad, unspecific nature of this theme, many generic phrases were included in the filtering lists. A tool which could potentially handle more flexibility is the open-source Medical Concept Annotation Toolkit (MedCAT) [24]. This is a novel self-supervised machine learning algorithm that uses concept vocabulary (including SNOMED-CT) for extracting concepts and also supports contextualization through unsupervised learning, matching ambiguous concepts to the best fitting overarching concepts.

To enable comparing the transdiagnostic measures we selected based on the qualitative study into clinical staff’s beliefs and literature review to an objective measure, a linear model with stepwise model selection was fitted with the transdiagnostic measures as predictors, and HDRS scores as outcomes. Ideally, one would compare the candidate outcome measures to an existing transdiagnostic outcome questionnaire such as SF36 to be able to extend this comparison beyond depressive symptoms, but these are not often part of routine clinical care and were not available for our retrospectively collected cohort. The second systematic review performed in this study however revealed that changes in HDRS scores are correlated with changes in SF36 scores. The HDRS quantifies depression symptoms; with this analysis, we have shown that several of the candidate outcome measures are associated with this gold standard. For the NLP themes, the themes reflecting social functioning and psychiatric symptoms were associated, perhaps reflecting the symptom-oriented nature of the HDRS. These associations might indicate that the theme scores developed in this study could potentially be used to measure treatment effects transdiagnostically, but to prove this, comparison with an objective transdiagnostic standard such as the SF36 or syndrome specific gold standards reflecting other mental illnesses would be necessary.

Not for all patients for whom clinical notes were available, sentences with changes were present for every theme in the clinical notes. This highlights the possibility of the existence of bias in these retrospective clinical notes: possibly, only more remarkable changes during treatment trajectories are denoted. When trying to gain qualitative insights into treatment trajectories for individual patients these “noisy” observations being omitted might actually be helpful, but when trying to create quantitative overviews or to find associations results could be misleading. This is an unavoidable challenge when trying to use existing data to develop predictive models, and warrants the need for prospective studies into the coherence between transdiagnostic outcomes measured through standardized questionnaires, and the content of clinical notes.

The research in this paper emphasizes the need for standardized outcome measures for comparing and combining machine learning models in mental health. The Core Outcome Measures in Effectiveness Trials (COMET) initiative has initiated this sort of work with the goal of streamlining clinical trial initiatives [25]; it would be interesting to further formalize standards for prediction models as well, as this would certainly aid working towards FAIR use of data and initiatives for sharing and collectively training machine learning models in healthcare. [26, 27].

## Conclusions

This paper highlights information extraction from clinical notes as a good alternative for standardized questionnaires when one aims to gain insight into treatment outcomes at their facility. We have shown that it is not only feasible to extract information on outcome measures of interest from clinical text, but we also validated that these transdiagnostic themes might accurately reflect treatment outcomes in a subgroup of patients with symptoms of depression, as compared with the Hamilton questionnaire. This approach has a closer connection to clinical practice and individual patients, as it is directly based on real data and clinical practice as opposed to measuring instruments for clinical research. From here forward, pipelines like this could be used to generate better insights into treatment outcomes for all patients in a cohort for which clinical notes are available, as opposed to only patients for which standardized questionnaires are available, a possible source of selection bias. Clinicians could for example be offered real-time insights into treatment outcomes for diverse patient groups at their department through a dashboard with summary statistics of all the outcome measures. An interesting addition would be the construction of a combined weighted outcome score, with weights for example based on a linear regression model, such as the one trained in this paper, with the HDRS scores at outcomes.

## Supplementary Information

**Additional file 1:** **Table 1.** Examples from the lists used for rule-based filtering ofthe four themes and change phrases. **Table 2. **Overview of clinical trials in psychiatry where atransdiagnostic outcome measurewas used, studied diagnoses and the outcome measures. **Table 3. **Ten most prevalent global outcome measures within the 362included clinical trials. **Table 4. **Clinical trials in psychiatry that both used the HDRS and theSF36 as outcome measures. For HDRS and SF36 scores in each intervention group,see the additional excel file (additional information 2). **Table 5. **Correlation coefficients ofthe HDRS and SF36 subscores. **Table6. **Specificity, sensitivity and positive predictive value of the SF36subcomponents for severity of depression, as compared to the HDRS. **Table 7. **EHR sourced assessed for extracting information on each outcomemeasure theme.

**Additional file 2:** 

## Data Availability

The datasets used and/or analyzed during the qualitative study and NLP comparison to HDRS scores are available from the corresponding author on reasonable request. Clinical notes are not available for sharing to ensure privacy of involved patients. All data generated or analyzed during the systematic reviews are included in the supplementary information files of this published article.

## Abbreviations

EHR: Electronic health records
HDRS: Hamilton depression rating scale
NLP: Natural language processing
SF-36: 36-Item Short-Form Survey for quality of life
