# Supplementary material for: Information extraction from free text for aiding transdiagnostic psychiatry: constructing NLP pipelines tailored to clinicians’ needs
Source: BMC Psychiatry. 2022 Jun 17;22:407. doi: 10.1186/s12888-022-04058-z (PMC9206307; doi:10.1186/s12888-022-04058-z)
Supplement: Supplementary file 1 — Additional file 1: Table 1. Examples from the lists used for rule-based filtering ofthe four themes and change phrases. Table 2. Overview of clinical trials in psychiatry where atransdiagnostic outcome measurewas used, studied diagnoses and the outcome measures. Table 3. Ten most prevalent global outcome measures within the 362included clinical trials. Table 4. Clinical trials in psychiatry that both used the HDRS and theSF36 as outcome measures. For HDRS and SF36 scores in each intervention group,see the additional excel file (additional information 2). Table 5. Correlation coefficients ofthe HDRS and SF36 subscores. Table6. Specificity, sensitivity and positive predictive value of the SF36subcomponents for severity of depression, as compared to the HDRS. Table 7. EHR sourced assessed for extracting information on each outcomemeasure theme. [file 12888_2022_4058_MOESM1_ESM.docx]

Additional methods and results for the manuscript “information extraction from free text for aiding transdiagnostic psychiatry: constructing NLP pipelines tailored to clinicians’ needs”

Dr. Rosanne J. Turner^1,2,*^, Femke Coenen^1^, Femke Roelofs^1^, Karin Hagoort^1^, Dr. Aki Härmä^3^, Prof. Peter D. Grünwald^2,4^, Dr. Fleur P. Velders^1^, Prof. Dr. Floortje E. Scheepers^1^

1: University Medical Center Utrecht, Brain Center, Netherlands

2: CWI, Machine Learning group, Netherlands

3: Philips research, Eindhoven, Netherlands

4: Leiden University, Department of Mathematics, Netherlands

# **Contents of additional information, file 1**

Section 1: Examples of theme and change phrases used for filtering sentences in the NLP pipeline

- 1. Additional table 1: examples from the lists used for rule-based filtering of the four themes and change phrases.

Section 2: Questionnaire for healthcare professionals on defining treatment goals and recovery

2.1 Questionnaire

2.2 Detailed methods thematic analysis

Section 3: detailed methods and results systematic review transdiagnostic outcome measured used in psychiatry research

3.1 Detailed methods

3.2 Search strategies

3.3 Overview of included studies

- additional table 2: Overview of clinical trials in psychiatry where a transdiagnostic outcome measure was used, studied diagnoses and the outcome measures.

3.4 Most prevalent transdiagnostic outcome measures

- additional table 3: ten most prevalent global outcome measures within the 362 included clinical trials

Section 4: detailed methods and results systematic review aptness of transdiagnostic outcome measure for detecting treatment effects compared to HDRS

4.1 Detailed methods

4.2 Search strategies

4.3 Overview included studies

- additional table 4: clinical trials in psychiatry that both used the HDRS and the SF36 as outcome measures. For HDRS and SF36 scores in each group, see the supplementary excel file.

4.4 Detailed results

- additional table 5: Correlation coefficients of the HDRS and SF36 subscores.
- additional table 6: Specificity, sensitivity and positive predictive value of the SF36 subcomponents for severity of depression, as compared to the HDRS.

Section 5: detailed results for quantitative assessment of feasibility of extracting information on outcome measure themes from the EHR

- additional table 7: EHR sourced assessed for extracting information on each outcome measure theme.

References for the Systematic Reviews

# **Online supplement section 1: Examples of theme and change phrases used for filtering sentences in the NLP pipeline**

| **Category** | **Dutch** | **Translation to English** | **Sentiment score** |
| --- | --- | --- | --- |
| Symptom reduction | Angstiger | More anxious | -1 |
|  | Angstigheid | Anxiety | -1 |
|  | Agressie | Aggression | -1 |
|  | Agresie | Aggression (misspelled) | -1 |
|  | Somber | Sad | -1 |
|  | Somer | Sad (misspelled) | -1 |
|  | Rotgevoel | Bad feeling | -1 |
|  | Doelloosheid | Aimlessness | -1 |
| Social functioning | Zelfstandig | Independent | 1 |
|  | Zelfstandige | Independent (conjugation) | 1 |
|  | Zelfstandigheid | Independence | 1 |
|  | Resocialiseren | Resocialize | 1 |
|  | Participeert | Participates | 1 |
|  | Vriendinnen | Girlfriends | 1 |
|  | Vriendschappen | Friendships | 1 |
|  | Verantwoordelijkheid | Responsibility | 1 |
| General well-being | Welbevinden | Well-being | 1 |
|  | Welzijn | Well-being (synonym) | 1 |
|  | Hoop | Hope | 1 |
|  | Zingeving | Meaning | 1 |
|  | Zinvol | Meaningful | 1 |
|  | Zelfwaardering | Self-esteem | 1 |
|  | Eigenwaarde | Self-esteem (synonym) | 1 |
|  | Zelfvertrouwen | Self-confidence | 1 |
|  | Zelfvetouwen | Self-confidence (misspelled) | 1 |
| Patient experience | Voelde | Felt | 1 |
|  | Nez | In their own words (abbreviated) | 1 |
|  | Voelt | Feels | 1 |
|  | Uitte | Expressed | 1 |
|  | Verwoorde | Articulated | 1 |
|  | Constateert | Noted | 1 |
|  | Merkt | Notes | 1 |
|  | Mekrt | Notes (misspelled) | 1 |
| Change indicator | Afnam | Decreased | -1 |
|  | Afname | Decrease | -1 |
|  | Afgenomen | Decreased (conjugation) | -1 |
|  | Toenemende | Increasing | 1 |
|  | Toenemde | Increasing (misspelled) | 1 |
|  | Verbeter | Improve | 1 |
|  | Verminder | Reduce | -1 |
|  | Vermindern | Reduce (misspelled) | -1 |

**additional table 1: examples from the lists used for rule-based filtering of the four themes and change phrases.**

# **Online supplement section 2: Questionnaire for healthcare professionals on defining treatment goals and recovery**

## **2.1 Questionnaire**

## Treatment and recovery

**Question:**
According to you, what is the most important goal of treatment (both outpatient and inpatient) on the psychiatry department?
* Treatment refers to all forms of offered care.

**Question:**
What is your definition of recovery?

**Question:**
Which form(s) of recovery do you work on during treatment?
(Multiple answers are possible, range them in order of importance)
☐ Clinical recovery (aimed at the reduction of symptoms)
☐ Social/societal recovery (aimed at daily functioning, participation)
☐ Personal recovery (own experience and general well-being of the patient)

**-----------------------------------------------------------------------------------------------------------------------------------**
Determination and registration of the efficacy of treatment in the EHR

*Below you will find a couple of questions about the determination of the efficacy of a treatment. With efficacious treatment we mean a treatment that contributes to the recovery of the patient.*

**Question:**
On what basis do you determine the efficacy of treatment?
(Multiple answers are possible)

**Question:**
When the treatment is efficacious, how and where is this registered in the EHR?
(Multiple answers are possible. For example, certain terms or scores that you use.)

**Question:**
According to you, where in the EHR do we find the most valuable information about the efficacy of treatment?

**Question**: Are there any aspects about the efficacy of treatment that you do not register in the EHR?
-----------------------------------------------------------------------------------------------------------------------------------
Aspects of recovery

**Question**: A couple aspects of recovery are listed below. Can you indicate per aspect how important this is to you in the determination of the treatment outcome?
Answering options: *Not important at all – Not important - Neutral- Important – Very important*

1. Symptom reduction
For example, less delusional, hallucinations, depressed or fewer behavioral problems.

2. Improved bodily functioning (independent of the disorder)
For example, feeling healthy and fit; having enough sleep, food and exercise.

3. Improved social societal functioning
For example, living independently and taking care of oneself; having a job, education or other goal oriented daily activities; or engaging in social activities.

4. Improved quality of life
For example, life satisfaction, feeling good in your own skin.

5. The degree of recovery according to the patient himself

6. The degree of recovery according to the family/system of the patient

**Question***:* Are there according to you any other aspects of recovery that must be taken into account?

## **2.2 Detailed methods thematic analysis**

The thematic analysis was performed by the first and second author (RJT and FC). First, the researchers read and familiarized themselves with the data and noted initial thoughts and interpretations. Second, the researchers coded the answers separately using software for analysis of qualitative data (NVivo 12). Inductive coding was used to identify data segments that were relevant to the research question. These codes were developed, compared and modified during the coding process. Next, the data were examined for broader patterns using mind maps. This led to the generation of semantic themes, which were checked against the data associated with the themes and the dataset as a whole. Accordingly, the themes were refined and detailed descriptions of the content and scope of the themes were written. Finally, the themes were named and data extracts were chosen to illustrate the themes.

# **Online supplement section 3: detailed methods and results systematic review transdiagnostic outcome measured used in psychiatry research**

## **3.1 Detailed methods**

The search was limited to clinical trials concerning adults with a psychiatric diagnosis in Pubmed and Embase; the full search can be found below. All studies were reviewed for inclusion by two separate researchers (RJT and AW). Study setups needed to contain an intervention, because the aim of this review was to find transdiagnostic outcome measures used in clinical trials that can measure treatment effects. Study populations had to comprise of patients with a psychiatric disorder as a primary diagnosis. Studies with a population with a primary psychiatric diagnosis and comorbidities were included as long as the intervention was aimed at the psychiatric disorder. Excluded were general outcome measures that were not captured in a questionnaire, such as MRI results and lab results, because the primary focus of this study was to find transdiagnostic outcome measures that can be extracted from existing data for a wide spectrum of patients.

## **3.2 Search strategies**

### **Search in Pubmed**

**#1**

Mental Disorder*[tiab] OR ”Mental Disorders”[Majr] OR Psychiatric disorder*[tiab] OR anxiety disorder*[tiab] OR psychosis[tiab] OR mood disorder*[tiab] OR "depressive disorder"[tiab] OR "clinical depression"[tiab] OR mania[tiab] OR bipolar[tiab] OR eating disorder*[tiab] OR schizophrenia[tiab] OR personality disorder*[tiab] OR substance abuse[tiab] OR substance-related disorder*[tiab] OR post-traumatic stress disorder*[tiab]

**#2**

”Patient Reported Outcome Measures”[Mesh:noexp] OR Patient Reported Outcome*[tiab] OR ”Mental Health Recovery”[Mesh] OR ”Mental Health Recovery”[tiab] OR functioning scale*[tiab] OR functioning score*[tiab] OR functioning assessment*[tiab] OR “global assessment of functioning”[tiab] OR global assessment scale*[tiab] OR functioning index*[tiab] OR ”quality of life”[Mesh] OR ”quality of life”[tiab] OR HRQOL[tiab] OR life quality[tiab] OR “Short-Form 36 Health Survey”[tiab] OR “Short-Form 12 Health Survey”[tiab] OR treatment response*[tiab] OR "response to treatment”[tiab] OR "responses to treatment”[tiab]

**#1 AND #2 = #3**

Delirium[MeSH] OR delirium[tiab] OR Alzheimer Disease[MeSH] OR Alzheimer[tiab] OR Dementia[tiab] OR cognitive disorder[title] OR Infan*[tiab] OR toddler*[tiab] OR minor[tiab] OR minors*[tiab] OR boy[tiab] OR boys[tiab] OR boyfriend[tiab] OR boyfriends[tiab] OR boyhood[tiab] OR girl[tiab] OR girls[tiab] OR girlfriend[tiab] OR girlfriends[tiab] OR kid[tiab] OR kids[tiab] OR child[tiab] OR children*[tiab] OR schoolchild*[tiab] OR school child*[tiab] OR adolescen*[tiab] OR juvenil*[tiab] OR youth*[tiab] OR teen*[tiab] OR underage*[tiab] OR pubescen*[tiab] OR puberty[tiab] OR pediatrics[MESH] OR pediatri*[tiab] OR paediatri*[tiab] OR school[tiab] OR school*[tiab] OR prematur*[tiab] OR preterm*[tiab] OR youth[tiab] OR youths[tiab] OR teen[tiab] OR teens[tiab] OR teenager[tiab] OR youngster*[tiab] OR child[MeSH] = #4

**#3 NOT #4**

**Filters: Clinical Trial; 5 years**

### **Search in Embase**

‘mental disease*’:ti,ab,kw OR 'mental disease'/mj OR ‘psychiatric disorder*’:ti,ab,kw OR ‘anxiety disorder*’:ti,ab,kw OR ‘psychosis’:ti,ab,kw OR ‘mood disorder*’:ti,ab,kw OR ‘depressive disorder’:ti,ab,kw OR ‘clinical depression’:ti,ab,kw OR ‘mania’:ti,ab,kw OR ‘bipolar’:ti,ab,kw OR ‘eating disorder*’:ti,ab,kw OR ‘schizophrenia’:ti,ab,kw OR ‘personality disorder*’:ti,ab,kw OR ‘substance abuse’:ti,ab,kw OR ‘substance-related disorder*’:ti,ab,kw OR ‘post-traumatic stress disorder*’:ti,ab,kw

**AND**

'patient-reported outcome'/de OR ‘patient reported outcome*’:ti,ab,kw OR 'mental health recovery'/de OR ‘mental health recovery’:ti,ab,kw OR ‘functioning scale*’:ti,ab,kw OR ‘functioning score*’:ti,ab,kw OR ‘functioning assessment*’:ti,ab,kw OR ‘global assessment of functioning’:ti,ab,kw OR ‘global assessment scale*’:ti,ab,kw OR ‘functioning index*’:ti,ab,kw OR 'quality of life'/exp OR ‘quality of life’:ti,ab,kw OR ‘HRQOL’:ti,ab,kw OR ‘life quality’:ti,ab,kw OR ‘Short-Form 36 Health Survey’:ti,ab,kw OR ‘Short-Form 12 Health Survey’:ti,ab,kw OR ‘treatment response*’:ti,ab,kw OR ‘response to treatment’:ti,ab,kw OR ‘responses to treatment’:ti,ab,kw

**NOT**

'delirium'/exp OR ‘delirium’:ti,ab,kw OR 'Alzheimer disease'/exp OR ‘Alzheimer’:ti,ab,kw OR ‘dementia’:ti,ab,kw OR ‘cognitive disorder’:ti OR ‘Infan*’:ti,ab,kw OR ‘toddler*’:ti,ab,kw OR ‘minor’:ti,ab,kw OR ‘minors*’:ti,ab,kw OR ‘boy’:ti,ab,kw OR ‘boys’:ti,ab,kw OR ‘boyfriend’:ti,ab,kw OR ‘boyfriends’:ti,ab,kw OR ‘boyhood’:ti,ab,kw OR ‘girl’:ti,ab,kw OR ‘girls’:ti,ab,kw OR ‘girlfriend’:ti,ab,kw OR ‘girlfriends’:ti,ab,kw OR ‘kid’:ti,ab,kw OR ‘kids’:ti,ab,kw OR ‘child’:ti,ab,kw OR ‘children*’:ti,ab,kw OR ‘schoolchild*’:ti,ab,kw OR ‘school child*’:ti,ab,kw OR ‘adolescen*’:ti,ab,kw OR ‘juvenil*’:ti,ab,kw OR ‘youth*’:ti,ab,kw OR ‘teen*’:ti,ab,kw OR ‘underage*’:ti,ab,kw OR ‘pubescen*’:ti,ab,kw OR ‘puberty’:ti,ab,kw OR 'pediatrics'/exp OR ‘pediatri*’:ti,ab,kw OR ‘school’:ti,ab,kw OR ‘school*’:ti,ab,kw OR ‘prematur*’:ti,ab,kw OR ‘preterm*’:ti,ab,kw OR ‘youth’:ti,ab,kw OR ‘youths’:ti,ab,kw OR ‘teen’:ti,ab,kw OR ‘teens’:ti,ab,kw OR ‘teenager’:ti,ab,kw OR ‘youngster*’:ti,ab,kw OR 'child'/exp

## **3.3 Overview of included studies**

**additional table 2: Overview of clinical trials in psychiatry where a transdiagnostic outcome measure was used, studied diagnoses and the outcome measures.**

| **Study ID** | **Diagnoses** | **Transdiagnostic outcome measures** |
| --- | --- | --- |
| Monfort 2019^1^ | Mental disorder and substance abuse | Life Skills Profile questionnaire (LSP); Barrat impulsiveness scale |
| Crowe 2020^2^ | Depression | Social Adjustment Scale. |
| Anjara 2019^3^ | Mental disorder | The Health of the Nation Outcome Scale (HoNOS); WHODAS 2.0; EQ-5D; Client Service Receipt Inventory (CSRI); Revised Clinical Interview Schedule (CIS-R) |
| Murata 2019^4^ | Depression | Mental Defeat Scale (MDS); EQ-5D; Cognitive Flexibility Scale (CFS) |
| Veselinovic 2019^5^ | Schizophrenia | SF-36; Subjective Well-being Under Neuroleptic Treatment Scale, Short Form (SWN-K), Cumulative Needs for Care Monitor (CNCM7, Van-Os scale) |
| Lewis 2019^6^ | Depression | SF-12; EQ-5D |
| Aubry 2019^7^ | Mental disorder | Multnomah Community Ability Scale (MCAS); EQ-5D; Colorado Symptom Index (CSI); Quality of Life Interview (QoLI-20); Recovery Assessment Scale (RAS) |
| Hahn 2019^8^ | Schizophrenia | QL; BPRS; UCSD Performance-Based Skills Assessment; MATRICS Consensus Cognitive Battery (MCCB) composite score |
| Xie 2019^9^ | Anxiety disorder | GAF; SF-12; Severity Subscale of the Clinical Global  Impression Scale (CGI-S) |
| Gilliam 2019^10^ | Depression | MINI; he Adverse Events Profile (AEP) |
| Adewuya 2019^11^ | Depression | WHODAS-12; WHOQOL-bref |
| Zhao 2019^12^ | Depression | WHOQOL-BREF |
| Wesner 2019^13^ | Panic disorder | CGI; The Resilience Scale; Coping Strategies Inventory (CSI); WHOQOL-bref |
| Philip 2019^14^ | PTSD | SOFAS; Q-LES-Q; |
| Hensel 2019^15^ | Mental disorder | RAS-r; EQ-5D-5L |
| Meng 2019^16^ | OCD | GAF; CGI-S |
| Serfaty 2019^17^ | Depression | EQ-5D-5L; Client Services Receipt Inventory (SCRI) |
| Chan 2019^18^ | Depression (postpartum) | SF-12 |
| Gomes 2019^19^ | Bipolar disorder | WHOQOL-bref; Functioning Assessment Short Test (FAST); Wechsler Abbreviated Scale of Intelligence; CANTAB |
| Mustafa 2019^20^ | Schizophrenia | GAF; SOFAS; CGI-S; BPRS |
| Puspitosari 2019^21^ | Schizophrenia | QOLI |
| Geraets 2019^22^ | Anxiety disorder | Manchester Short Assessment of Quality of Life (MANSA); Paranoid Thought Scales (GPTS) |
| Mak 2019^23^ | Anxiety disorder | EQ-5D-5L |
| Best 2019^24^ | Schizophrenia | Specific Levels of Functioning Scale (SLOF); Canadian Objective Assessment of Life Skills-“Brief; BPRS; Sheehan Disability Scale (SDS); Need for Cognition Scale |
| Gowda 2019^25^ | OCD | CGI |
| Wilhelm 2019^26^ | Body Dysmorphic Disorder | Brown Assessment of Beliefs Scale (BABS); Sheehan Disability Scale (SDS); Q-LES-Q-SF |
| van Emmerik-van Oortmerssen 2019^27^ | ADHD; SUD | EQ-5D |
| Jensen 2019^28^ | Schizophrenia; Bipolar disorder | GAF; Personal and Social Performance (PSP) scale; Mental Health Recovery Measure (MHRM); Adult Hope Scale (AHS); Client Satisfaction Questionnaire (CSQ) |
| Bressington 2019^29^ | Depression | SF-12; CSQ |
| Kählke 2019^30^ | Anxiety disorder | BSI; Assessment of Quality of Life (AQoL); CSQ; Inventory of Interpersonal Problems (IIP) |
| Quilty 2019^31^ | Eating disorder | QOLI; UPPS-P Impulsive Behavior Scale |
| Berk 2019^32^ | Bipolar disorder | CGI-I; CGI-S; patient Global Impression scale; SOFAS; Longitudinal Interval Follow-Up Evaluation (LIFE); Range of Impaired Functioning Tool (LIFE-RIFT); Q-LES-Q-SF |
| van Dijk 2019^33^ | Personality disorder | BSI-53; EQ-5D-5L; Cantril Ladder; WEMWBS |
| Kilbourne 2019^34^ | Depression; Bipolar disorder | SF-12 |
| Cho 2019^35^ | Schizophrenia | subjective Well-Being Under Neuroleptic Treatment Scale; Berkeley Expressivity Questionnaire |
| Pos 2019^36^ | Schizophrenia | GAF; Dysfunctional Attitudes Scale-Defeatist Performance Attitude (DAS-DPA) |
| Pezzin 2018^37^ | PTSD | EuroQoL |
| Saglam Aykut 2019^38^ | Schizophrenia | CGI; SF-36 |
| Jelinek 2019^39^ | Depression | WHOQOL-BREF |
| Bauer 2018^40^ | Bipolar disorder | GAF |
| Vieta 2019^41^ | Depression | CGI-S |
| Howard 2018^42^ | VLOSLP | BPRS; EuroQol-5; WHOQoL-BREF |
| Lee 2019^43^ | OCD | SF-36; Oxford Happiness Questionnaire (OHQ); Warwick-Edinburgh Mental Well-Being Scale (WEMWBS); Sheehan Disability Scale (SDS) |
| Possemato 2019^44^ | PTSD | The WHOQOL-BREF; Connor-Davidson Resilience Scale (CD-RISC); Client Satisfaction Questionnaire (CSQ); Patient Activation Measure for Mental Health (PAM-MH) |
| Kessler 2018^45^ | Depression | EQ-5D-5L; SF-12 |
| Kulz 2019^46^ | OCD | WHOQOL-BREF; CSQ; Distress Tolerance Scale (DTS); Kentucky Inventory of Mindfulness Skills (KIMS); self-Compassion-Scale (SCS); Metacognitions Questionnaire (MCQ-30) |
| Sanz 2018^47^ | Adjustment disorder | Health Questionnaire; SF-12; Revised Symptom Inventory, SCL-90-R |
| Kessler 2018^48^ | Depression | SF-12; EQ-5D-5L |
| Muralidharan 2019^49^ | Mental disorder | 6-item Self-Management Self-Efficacy scale (SSE-6); SF-12; 6-item Internal Health Locus of Control (IHLC); Maryland Assessment of Recovery Scale (MARS); The Instrument to Measure Self-Management (IMSM); behavior and Symptom Identification Scale 24 - Modified (BASIS) |
| Blanco 2019^50^ | Depression | CGI-S; QLESQ; CSQ; SF-12 |
| Rosengard 2019^51^ | Psychosis | GAF; SOFAS |
| Mahmood 2019^52^ | Psychosis | Quality of Life Interview (QOLI) |
| Jahoda 2018^53^ | Depression | EuroQol-5 Dimensions -“ Youth version (EQ-5D-Y); Behavior Problems Inventory for Individuals with Intellectual Disabilities -“ Short Form (BPI-S); Community Involvement (ICI); Index of Participation in Domestic Life (IPDL); Social Support Questionnaire -“ three questions (SSQ3) |
| Sajatovic 2018^54^ | Bipolar disorder | Brief Psychiatric Rating Scale (BPRS); GAF; CGI |
| Halverson 2019^55^ | Schizophrenia | Specific Level of Functioning Scale (SLOF); The Interpersonal Perception Task (IPT); Interpersonal Reactivity Index (IRI); Paranoid Thought Scales (GPTS); |
| Schennach 2019^56^ | Schizophrenia | GAF; SOFAS |
| Petzold 2019^57^ | Mental disorder | Symptom-Checklist-27 (SCL-27) part of Symptom-Checklist-90 Revised (SCL-90-R); SF-12; Physical Activity Questionnaire (IPAQ); Food-Frequency Questionnaire |
| Petersen 2018^58^ | Depression | EQ-5D |
| Meltzer 2018^59^ | Depression (postpartum) | CGI-I |
| Conway 2018^60^ | Depression | Q-LES-Q-SF |
| Stein 2018^61^ | Anxiety disorder | CGI-S; Toronto Hospital Alertness Test; Leeds Sleep Evaluation Questionnaire Scores |
| Sarris 2018^62^ | Depression | SF-12; CGI; Leeds Sleep Evaluation Questionnaire (LSEQ) |
| Ertekin 2019^63^ | Schizophrenia | WHOQOL-BREF |
| Gao 2018^64^ | Bipolar disorder | CGI-EI; Q-LES-Q-SF; Sheehan Disability Scale (SDS); Iowa Fatigue Scale (IFS) |
| Cladder 2018^65^ | Depression | MINI; WHOQOL; Ruminative Response Scale (RRS-EXT); Five Facet Mindfulness Questionnaire; Self-Compassion Scale |
| Kool 2018^66^ | Depression; Personality disorder | MINI; Brief Symptom Inventory (BSI); Outcome Questionnaire-45 (OQ-45); EuroQol 5D (EQ-5D-5 L); Structured Clinical Interview for DSM-IV personality disorders (SCID-II) |
| Menon 2018^67^ | Bipolar disorder | WHOQOL-Bref |
| Reme 2019^68^ | Mental disorder | WODAS; Subjective Health Complaints Inventory; Euro-Qol Visual Analog Scale |
| Tendler 2018^69^ | Depression | CGI-S |
| Geramita 2018^70^ | Mental disorder | SF-12 |
| Björk Brämberg 2018^71^ | Mental disorder | EQ-5D; Work ability index |
| Dalton 2018^72^ | Anorexia | EQ-5D |
| Emsley 2018^73^ | Depression | CGI-S; CGI-I |
| Lequimener 2019^74^ | Bipolar disorder | WHOQOL-Bref; GAF; Rosenberg Self-Esteem Scale; Birchwood Insight Scale; |
| Luo 2019^75^ | Schizophrenia | (WHOQOL -“ BREF); CGI; Personal and Social Performance Scale; Social Disability Screening Schedule (SDSS0 |
| Iglesias 2018^76^ | Depression | WHODAS; EQ-5D |
| Yesavage 2018^77^ | Depression | Veterans RAND 36-item Health Survey |
| Schlosser 2018^78^ | Schizophrenia | Quality of Life Scale - Abbreviated (QOL-A); Motivation and Pleasure-Self Report scale (MAP-SR); Role Functioning Scale (RFS); Dysfunctional Attitudes Scale; BDI; Revised Self-Efficacy Scale (R-SES) |
| Delgadillo 2018^79^ | Depression; Anxiety disorder | WSAS |
| Patel 2018^80^ | OCD | Q-LES-Q-SF |
| Li 2018^81^ | Depression | EQ-5D |
| Gliddon 2019^82^ | Bipolar disorder | Q-LES-Q |
| Bormann 2018^83^ | PTSD | WHOQOL; Insomnia Severity Index; Five Facet Mindfulness Questionnaire; State-Trait Anger Inventory-Short Form |
| Turakitwanakan 2017^84^ | Depression | WHOQOL-BREF-THAI |
| Ben-Zeev 2018^85^ | Mental disorder | Symptom-Checklist-27 (SCL-27) part of Symptom-Checklist-90 Revised (SCL-90-R); RAS |
| El Hage 2018^86^ | Depression | WHOQOL-BREF; CGI |
| Sanches 2018^87^ | Mental disorder | The WHOQOL-BREF; Brief Psychiatric Rating Scale (BPRS) |
| Renna 2018^88^ | Depression; Anxiety disorder | The Sheehan Disability Scale (SDS); The Quality of Life Inventory (QOLI); The Difficulties in Emotion Regulation Scale (DERS); Five Facets of Mindfulness Questionnaire (FFMQ); The Affect Intensity Measure-”Negative Intensity Subscale (AIM-NI); The Emotion Regulation Questionnaire-”Reappraisal Subscale (ERQ-R); The Experiences Questionnaire-”Decentering Subscale (EQ-D); The State Trait Anxiety Inventory-“7 (STAI-7); The Perseverative Thinking Questionnaire (PTQ); The Brooding Subscale of the Rumination Scale (RS); The Penn State Worry Questionnaire (PSWQ) |
| de Jong 2019^89^ | Schizophrenia | Indiana Psychiatric Illness Interview; CGI; MINI; Personal and Social Performance Scale (PSP); Self-Rated Manchester Short Assessment of Quality of Life  (MANSA); Metacognition Assessment Scale-A (MAS-A); Beck Cognitive Insight Scale (BCIS); Empathic Accuracy Task; Interpersonal relativity index; Questionnaire of Cognitive and Affective Empathy |
| Kamijima 2018^90^ | Depression | CGI; Social Adaptation Self-Evaluation Scale (SASS) |
| Samalin 2018^91^ | Bipolar disorder | Clinician Rating Scale (CRS); WHOQOL; Decision Conflict Scale; Decision-making Involvement and Satisfaction Scale (CDIS) |
| Nishida 2018^92^ | Psychosis | GAF; WHOQOL 26 |
| Crawford 2018^93^ | Borderline Personality Disorder | EQ-5D-3L; Acts of Deliberate Self-Harm Inventor; social functioning questionnair SFQ |
| Li 2018^94^ | Schizophrenia | BPRS; GAF; Self-Esteem Scale (SES); Stigma of Mental Illness scale (ISMI), Discrimination and Stigma Scale (DISC-12), |
| Dalum 2018^95^ | Mental disorder | GAF |
| Shimada 2018^96^ | Schizophrenia | GAF; social functioning scale; CSQ; |
| Porcu 2018^97^ | Depression | SDS, CGI |
| Moritz 2018^98^ | Depression | WHOQOL-BREF |
| Chen 2019^99^ | Bipolar disorder | CGI |
| Tjak 2018^100^ | Depression | European Health Interview Surveys Quality of Life Scale (EUROHIS) |
| Hui 2018^101^ | Schizophrenia | SF-37; SOFAS; Role functioning scale |
| Peña 2018^102^ | Schizophrenia | UCSD Performance-based Skills Assessment (UPSA); GAF |
| Mennin 2018^103^ | Anxiety disorder | QOLI; SDS; CGI; Penn State Worry Questionnaire (PSWQ); Rumination Scale; |
| Stiekema 2018^104^ | Mental disorder | Health of the Nation Outcome Scales (HoNOS) and the Manchester Short Assessment of Quality of Life (MANSA) |
| Berle 2018^105^ | PTSD | Personal Wellbeing Index |
| du Sert 2018^106^ | Schizophrenia | Q-LES-Q-SF |
| Schottle 2018^107^ | Schizophrenia | BPRS; CGI-S; GAF; Q-LES-Q-18; CSQ |
| Laurenssen 2018^108^ | Borderline Personality Disorder | EQ-5D-3L; GSI; self-harm inventory |
| Najavits 2018^109^ | PTSD | GSI; Q-LES-Q-SF; Coping Self-Efficacy Scale; CGI; World Assumptions Scale |
| Harned 2018^110^ | PTSD; Borderline Personality Disorder | Global Adjustment Scale (GAS); Quality of Life Inventory (QOLI); Global Social Adjustment (GSA); |
| Haji 2018^111^ | Schizophrenia | WHOQOL; Wechsler Memory Scale |
| Bjorkelund 2018^112^ | Depression | EQ-5D |
| Lin 2018^113^ | Schizophrenia | QOLS; GAF |
| O'Haire 2018^114^ | PTSD | Satisfaction With Life Scale (SWLS); The Bradburn Scale of Psychological Wellbeing (BSPW); The Work Productivity and Activity Impairment Questionnaire; Connor Davidson Resilience Scale (CDRS); PROMIS Ability to Participate in Social Activities |
| Savitz 2018^115^ | Bipolar disorder | CGI-I |
| Hoxhaj 2018^116^ | ADHD | BSI; SF-36 |
| Mergl 2018^117^ | Depression | psychiatric rating scales (PSR); GAF |
| Bryce 2018^118^ | Schizophrenia | EUROHIS-QOL; MATRICS Consensus Cognitive Battery; Revised Self-Efficacy Scale (RSES); Independent Living Skills Survey-Self Report (ILSS-SR; |
| Aftab 2018^119^ | Bipolar disorder | BPRS; GAF |
| Gaughran 2017^120^ | Psychosis | SF-36; Dietary Instrument for Nutrition Education (DINE); Physical Activity Questionnaire (IPAQ-SF); GAF |
| Di Lorenzo 2018^121^ | Schizophrenia | psychiatric hospitalizations and urgent consultations required; CGI-S; CGI-I; GAF |
| Penades 2018^122^ | Schizophrenia | EQ-5D; Wechsler Adult Intelligence Scale-Third Revision; Wechsler Memory Scale-Third Edition; |
| Inchausti 2018^123^ | Schizophrenia-Spectrum Disorders | SOFAS; Personal and Social Performance Scale (PSP); Metacognition Assessment Interview (MAI); MAS-A |
| Corrigan 2018^124^ | Mental disorder | Service engagement with weekly contact report health-related appointments; e Righteous Anger (RA) subscale of the 28-item Empowerment Scale; 24-item Recovery Assessment Scale (RAS); Quality of Life Scale (QLS) |
| McGuire 2018^125^ | Schizophrenia | GAF; CGI-I; CGI-S |
| Sahin 2018^126^ | Borderline Personality Disorder | DIP-I; Symptom Checklist 90-Revised; GAF |
| Bitter 2017^127^ | Mental disorder | MANSA; Social Functioning Scale (SFS); Mental Health Recovery Measure (MHRM); Dutch Empowerment Scale; Herth Hope Index (HHI); Mental Health Confidence Scale (MHCS); Camberwell Assessment of Needs Short Appraisal Schedule (CANSAS) |
| Chang 2018^128^ | Mental disorder | Rosenberg Self-Esteem Scale (RSES); WHOQOL-BREF |
| Younan 2018^129^ | complex trauma syndrome | Rosenberg Self-Esteem Scale; Brief Symptom Inventory (BSI); WHOQOL-BREF; Schema Mode Inventory (SMI) |
| Katsumi 2019^130^ | Schizophrenia | GAF |
| Calabrese 2018^131^ | Bipolar disorder | Time to hospitalization; Functioning Assessment Short Test (FAST) |
| Bosanquet 2017^132^ | Depression | EQ-5D; Connor-Davidson Resilience Scale-2 items; adverse events; medication questionnaire |
| Berger 2018^133^ | Depression | SF-12; CSQ-8 |
| Goldstein 2018^134^ | PTSD | WHOQOL-BREF; Feasibility and Acceptability Questionnaire; Godin Leisure-Time Exercise Questionnaire |
| Shimizu 2017^135^ | Schizophrenia | SF-36; Frontal assessment battery (FAB) |
| Huang 2018^136^ | Depression | Q-LES-Q-SF; CGI-S |
| Sibeko 2017^137^ | Mental disorder | GAF; MARS; EUROQUEL; adherence to appointments; re-admissions |
| Eklund 2017^138^ | Mental disorder | Profiles of Occupational Engagement among people with Severe mental illness (POES); Satisfaction with daily occupations and occupational balance (SDO-OB); Occupational Value with predefined items (OVal-pd); Manchester Short Assessment of Quality of Life (MANSA); Rosenberg self-esteem scale (RSES); SF-36; GAF |
| Rollman 2018^139^ | Mood disorder; Anxiety disorder | SF-12 |
| Cohen 2017^140^ | Anxiety disorder | CGI; QOLI |
| Azevedo 2017^141^ | Depression | SF-36 |
| Girgis 2018^142^ | Schizophrenia | CGI-I; CGI-S; GAF; MATRICS consensus cognitive battery |
| Kivelitz 2017^143^ | Depression | SF-8, EQ-5D; self-efficacy (SWE); proportion of patients initiating outpatient psychotherapy |
| Castillo 2018^144^ | Depression | SF-12; hospitalizations; homelessness risk; mental wellness |
| Ruesch 2017^145^ | Depression; Adjustment disorder | Brief Symptom Inventory (BSI); SF-12 |
| Gordon 2018^146^ | Schizophrenia | Bell-Lysaker Emotion Recognition Task; Ambiguous Intentions Hostility Questionnaire-Ambiguous items (AIHQ-A); Social Cognition Screening Questionnaire (SCSQ); Social Skills Performance Assessment (SSPA); QLS; The Life Skills Profile 16 (LSP-16) |
| Contreras 2018^147^ | Schizophrenia | MATRICS Consensus Cognitive Battery (MCCB); self-report 20-item Self-Esteem Rating Scale short form (SERS); 8-item Quality of Life Scale (EUROHIS-QoL); 5-item Friendship Scale (FS); |
| Schjerning 2018^148^ | Schizophrenia | WHOQOL-BREF; CGI-S; CGI-I; Leeds Sleep Evaluation Questionnaire (LSEQ); Personal and Social Performance Scale (PSP); |
| Buchheim 2017^149^ | Borderline Personality Disorder | GAF |
| Vergunst 2017^150^ | Psychosis | The Social Network Schedule (SNS); The Objective Social Outcomes Index (SIX); EQ-5D-3L; Oxford Capability Questionnaire for Mental Health (OxCAP-MH); The Admission Experience Survey (AES); BPRS; GAF |
| Husain 2017^151^ | Depression | CGI; EQ-5D |
| Richards 2017^152^ | Depression | SF-36; EQ-5D |
| Lima 2018^153^ | Depression; Bipolar disorder | CGI |
| Baruah 2018^154^ | OCD | CGI |
| Gershkovich 2017^155^ | Anxiety disorder | Sheehan Disability Scale (SDS); Quality of Life Inventory (QOLI); Client Satisfaction Survey (CSS); CGI |
| de Zwaan 2017^156^ | Eating disorder | RSES; Clinical Impairment Assessment (CIA) |
| Bozzatello 2017^157^ | Borderline Personality Disorder | CGI-S; SOFAS; Barratt Impulsiveness Scale (BIS-11); Modified Overt Aggression Scale (MOAS); Self-Harm Inventory (SHI) |
| Ong 2017^158^ | Depression | 12-item mental composite score (MCS-12); 12-item physical composite score, PCS-12); hospitalization |
| Valimaki 2017^159^ | Psychosis | hospitalization; Client Satisfaction Questionnaire-8 [CSQ-8]; Q-LES-Q; Global Assessment Scale [GAS]; adverse events |
| Khan 2019^160^ | Mental disorder | WODAS 2.0 |
| Zoellner 2017^161^ | PTSD | (CGI-I); SCID-IV; Automated Operation Span (OSPAN); Subjective Units of Distress Scale (SUDS); SDS; Medication Effects Form (MEF); SF-36 |
| Wessels 2019^162^ | Psychosis | GAF |
| Lovell 2017^163^ | OCD | SF-36; Clinical Outcomes in Routine Evaluation (CORE-OM); IAPT Employment Status Questions (A13-A14); WSAS; Clinical Interview Schedule-Revised; CIS-R; Client Satisfaction Questionnaire (CSQ-8-UK) |
| Gabbay 2017^164^ | Depression | Short Warwick-“Edinburgh Mental Wellbeing Scale (SWEMWBS); Client Service Receipt Inventory (CSRI) ; EQ-5D-5L; MANSA; Stanford Presenteeism Scale; CAB/Control Debt Assessment and Outcomes questionnaire; General Satisfaction Questionnaire (GSQ); Hope Trait Scale; Life Events and Difficulties Schedule-short (LED-S); Other as Shamer scale (OAS) |
| Biesheuvel 2017^165^ | Depression | Structured Clinical Interview for DSM-IV axis 1 disorders; SF-12; EQ-5D |
| Driessen 2017^166^ | Depression | Outcome Questionnaire (OQ-45); Brief Symptom Inventory - Global Severity Index (BSI-GSI); pain (VAS); EQ-5D |
| Citrome 2018^167^ | Schizophrenia | CGI-S; CGI-I |
| Watanabe 2017^168^ | Depression | Social Adjustment Scale-Self-Report (SAS-SR); Q-LES-Q; Life Enjoyment Scale Short Version (LES-S); SF-36 |
| Eriksson 2017^169^ | Depression | The General Health Questionnaire (GHQ-12); EQ-5D |
| Dean 2017^170^ | Depression | Q-LES-Q; CGI-I; CGI-S; Range of Impaired Functioning Tool (LIFE-“RIFT); SOFAS |
| Hellstrom 2017^171^ | Mood disorder; Anxiety disorder | employment or education; WHO-Five Well-being Index (WHO-5); GAF; SDS; Empowerment Scale; Changes Questionnaire; CSQ |
| Sajatovic 2017^172^ | Mental disorder | CGI; GAF; BPRS; SDS; SF-36 |
| Hasan 2017^173^ | Schizophrenia | Modified Learned Helplessness Scale (MLHS; LHS); Recovery Assessment Scale (RAS) |
| Videler 2018^174^ | Personality disorder | strength of idiosyncratic beliefs; SCID-II; Symptom Checklist 90 (SCL-90); WHOQOL-BREF; target complaints/problem points; Dutch Young Schema Questionnaire (YSQ) |
| Hansson 2017^175^ | Mental disorder | Self-Stigma of Mental Illness Scale-Short Form (SSMIS-SF); Rosenberg self-esteem scale (RSES); MANSA |
| Moreno 2017^176^ | Bipolar disorder | reduction of affective episodes; Screen for Cognitive Impairment in Psychiatry; Mayer-Salovey-Caruso Emotional Intelligence Test; Functioning Assessment Short Test (FAST); The Holmes-Rahe Life Stress Inventory |
| Knaevelsrud 2017^177^ | PTSD | Brief Symptom Inventory-18 (BSI-18); General Self-Efficacy Scale (GSE); EUROHIS-QOL 8-item index (EUROHIS) |
| Mahlke 2017^178^ | Mental disorder | General Self-Efficacy Scale (GSE); EQ-5D; GAF |
| Kendrick 2017^179^ | Depression | WSAS; EQ-5D; Client Services Receipt Inventory; Medical Informant Satisfaction Scale (MISS) |
| Ngai 2017^180^ | Depression (postpartum) | SF-12 |
| Kang 2017^181^ | Schizophrenia | CGI-S |
| Schafer 2017^182^ | PTSD; Substance Use Disorder | SF-12; Dissociative Experiences Scale (DES); Difficulties in Emotion Regulation Scale, DERS); Serious Adverse Events (SAE) |
| Si 2017^183^ | Depression | SF-12 |
| Awan 2017^184^ | Schizophrenia | Short Explanatory Model Interview (SEMI); BPRS; Compliance Rating Scale |
| Wiltink 2017^185^ | Anxiety disorder | CGI; EQ-5D; treatment duration |
| Kloep 2017^186^ | PTSD | The Quality of Life Scale (QOLS); Dimensions of Anger Reactions-5 (DAR-5) |
| Sankhe 2017^187^ | Depression; Anxiety disorder | WHOQOL-BREF |
| Ishoy 2017^188^ | Schizophrenia | Rey-Osterreith complex figure test (REY); SF-36; Personal and Social Performance Scale (PSP) |
| Bewernick 2017^189^ | Depression | SF-36; GAF |
| Berk 2017^190^ | Bipolar disorder | BPRS; GAF; Vocation & Location Index; SOFAS |
| de Jong 2018^191^ | Depression | Global Impression of Change Questionnaire (PGIC); Brief Pain Inventory short form (BPI-sf); SF-36 |
| Bambling 2017^192^ | Depression | Outcome Questionnaire (OQ-45); quality of life (QoL) |
| Schramm 2017^193^ | Depression | GAF; SF-12 |
| Morozova 2017^194^ | Schizophrenia | CGI-I; CGI-S; Personal and Social Performance Scale |
| Lin 2017^195^ | Schizophrenia | CGI-S; GAF |
| Tomba 2017^196^ | Eating disorder | General Health Questionnaire (GHQ-30); Psychological Well-being Scales (PWB) |
| Wikberg 2017^197^ | Depression | EQ-5D; sick leave; health care use |
| Corrigan 2017^198^ | Mental disorder | Recovery Assessment Scale (RAS); Quality of Life Scale (QLS); TCU Health Form (TCU-HF); SF-36 |
| Theleritis 2017^199^ | Depression | CGI-S |
| Kwong 2017^200^ | Psychosis | SF-36; SOFAS |
| Tan 2017^201^ | Mental disorder | Illness management and recovery scale (IMRS); BPRS; Global assessment scale (GAS) |
| Cha 2017^202^ | Depression | global index of neurocognition; Q-LES-Q SF; SDS |
| Lee 2016^203^ | Bipolar disorder | Wisconsin Card Sorting Test (WCST); Conners' Continuous Performance Test (CPT); WHOQOL |
| Gumley 2017^204^ | Depression | Process of Recovery Questionnaire (QPR) |
| de Roten 2017^205^ | Depression | GAF |
| Abramovitch 2017^206^ | Tic disorder | CGI-I |
| Briest 2017^207^ | Depression | Coping Strategies Questionnaire (CSQ-D); SF-36 |
| Robinson 2016^208^ | Eating disorder | GAF; EQ-5D; Adult Service Use Schedule (AD-SUS) |
| Chen 2017^209^ | Eating disorder | GAF |
| Lagomasino 2017^210^ | Depression | SF-12; health care use; medication use |
| Asnaani 2017^211^ | OCD | QLESQ-SF; Social Adjustment Scale-Self-Report (SAS-SR); SDS |
| Mansson 2017^212^ | Anxiety disorder | CGI |
| O'Donnel 2016^213^ | Psychosis | BPRS |
| Egede 2016^214^ | Depression | SF-36; Charleston Psychiatric Outpatient Satisfaction Scale |
| Stern 2016^215^ | ADHD | IntegNeuroTM assessment; Ecological measures of everyday functioning and quality of life; BRIEF-A; Canadian Occupational Performance Measure (COPM); AAQoL Scale |
| Sanacora 2017^216^ | Depression | CGI; SDS |
| Kang 2016^217^ | Schizophrenia | WHOQOL-BREF; agression; hospitalization; medication adherence |
| Dunayevich 2017^218^ | Schizophrenia | Q-LES-Q-SF; SDS |
| Chen 2016^219^ | Depression | Q-LES-Q-SF |
| Arnedt 2016^220^ | Depression | SF-12 |
| Stergiopoulos 2016^221^ | Mental disorder | housing stability: Residential Time Line Follow-Back (RTLFB) Inventory; EQ-5D; Colorado Symptom Index (CSI); community functioning MCAS; Quality of Life Index (QoLI-20; Health Service and Justice Service Use Questionnaire (HSJSU) |
| Mora 2016^222^ | Bipolar disorder | FAST |
| Danovitch 2017^223^ | Depression; alcohol use disorder | Q-LES-Q; WSAS; Psychiatric Diagnostic Screening Questionnaire (PDSQ) |
| Young 2017^224^ | ADHD | Recovery Assessment Scale-revised (RAS-r); EQ-5D-5L; 15-item CIQ |
| Shimada 2016^225^ | Schizophrenia | GAF |
| Rosen 2017^226^ | PTSD | Conflict Tactics Scale (for agressive behavior) |
| Eustis 2016^227^ | Anxiety disorder | Acceptance and Action Questionnaire (AAQ); Penn State Worry Questionnaire (PSWQ); Quality of Life Inventory (QOLI); Experiences Questionnaire-”Decentering Subscale (EQ-Decentering; |
| Seki 2016^228^ | Panic disorder | EQ-5D |
| Rollman 2017^229^ | Anxiety disorder | SF-36 |
| Berger 2017^230^ | Anxiety disorder | BSI; GSI; Body Sensations Questionnaire (BSQ; 16-item Penn State Worry Questionnaire (PSWQ; CSQ |
| Pankowski 2017^231^ | Bipolar disorder | QOLI; Acceptance and Action Questionnaire - AAQ-2 |
| Rossell 2016^232^ | Schizophrenia | Quality of Life Profile (LQoLP) |
| Fiszdon 2016^233^ | Psychosis | UCSD Performance-Based Skills Assessment (UPSA); Social Skills Performance Assessment (SSPA); Medication Management Ability Assessment (MMAA); Independent Living Skills Survey (ILSS); Quality of Life Scale (QLS; Wisconsin Card Sorting Test; Trail Making Test Part B; Wechsler Memory Scale; California Verbal Learning Test-II |
| Yeung 2016^234^ | Depression | Q-LES-Q; CGI-I; CGI-S |
| Ngo 2016^235^ | Depression | Health care use; employment |
| Kidd 2018^236^ | Schizophrenia | The Multnomah Community Ability Scale (MCAS); Satisfaction With Life Scale (SWLS; 31-item Involvement Evaluation Questionnaire (IEQ; |
| Goodman 2017^237^ | ADHD | CGI-I; Continuous Performance Test (CPT); Behavior Rating Inventory of Executive Function-Adult (BRIEF-A); Dyadic Adjustment Scale (DAS)-Satisfaction subscale |
| Richards 2016^238^ | Depression | SCID; SF-36 |
| Aardoom 2016^239^ | Eating disorder | Eq-5D |
| Steuwe 2016^240^ | Borderline Personality Disorder | WHOQOL |
| McMurran 2016^241^ | Personality disorder | Social Functioning Questionnaire (SFQ); EQ-5D |
| Ritzert 2016^242^ | Anxiety disorder | Penn State Worry Questionnaire (PSWQ); QOLI; |
| Muntingh 2016^243^ | Depression | WHOQOL |
| Zoun 2016^244^ | Depression; Anxiety disorder | WHOQOL |
| Gingnell 2016^245^ | Anxiety disorder | CGI-I |
| Otto 2016^246^ | Panic disorder | role functioning (LIFE-RIFT); Q-LES-Q |
| Engel 2016^247^ | PTSD; depression | SF-12 |
| Juretic 2016^248^ | Schizophrenia | MANSA |
| Rakitzi 2016^249^ | Schizophrenia | GAF; WHOQOL |
| Grunder 2016^250^ | Schizophrenia | SF-36; CGI-I |
| Atiwannapat 2016^251^ | Depression | SF-36 |
| Yoon 2016^252^ | Schizophrenia | GAF; Schedule of Recent Experience (SRE); Personal Behavioral Analysis (PBA); WHOQOL; adherence |
| Rajagopalan 2016^253^ | Bipolar disorder | Q-LES-Q |
| Gelkopf 2016^254^ | Mental disorder | MANSA; colorado Symptom Index (CSI); psychiatric health care use |
| Schaap 2016^255^ | Personality disorder | The Brief Symptom Inventory (BSI); The Young Schema Questionnaire (YSQ; ); The Short Schema Mode Inventory (SMI; The Young Compensation Inventory (YCI); The Young-Rygh Avoidance Inventory (YRAI); The Mental Health Continuum-Short Form (MHC-SF |
| Ito 2016^256^ | Depression; Anxiety disorder | GAF; CGI; SDS; EQ-5D |
| Bozzatello 2016^257^ | Borderline Personality Disorder | CGI-S; SOFAS; Satisfaction Profile (SAT-P) |
| Berry 2016^258^ | Psychosis | GAF |
| Barnes 2016^259^ | Schizophrenia | Heinrich's Quality of Life Scale |
| Makkos 2016^260^ | Depression | PDQ-39 summary index (PDQ-39 SI) |
| Isitt 2016^261^ | Schizophrenia | EuroQol EQ-5D-5L; |
| Chang 2016^262^ | Psychosis | Employment; Role Functioning Scale (RFS); SOFAS |
| Richards 2016^263^ | Depression | EQ-5D-3L; SF-36; CSQ |
| Dagani 2016^264^ | Schizophrenia | State Trait Anger Expression Inventory2 (STAXI 2) ; WHOQOL-BREF; Self Esteem Rating Scale (SERS); Questionnaire on the social network (SNQ) |
| Priebe 2016^265^ | Schizophrenia | MANSA; SIX; size of social network SNS; EQ-5D; CSQ |
| Omer 2016^266^ | Psychosis | MANSA |
| Huijbers 2016^267^ | Depression | SCID-I; WHOQOL |
| Meredith 2016^268^ | PTSD | SF-12 |
| Wong 2016^269^ | Anxiety disorder | Penn State Worry Questionnaire (PSWQ); SF-12; Five Facet Mindfulness Questionnaire (FFMQ) |
| Nierenberg 2016^270^ | Bipolar disorder | Longitudinal Interval Follow-up Evaluation-Range of Impaired Functioning Tool (LIFE-RIFT); Clinical Global Impressions-Efficacy Index (CGI-EI); clinical adjustment |
| Enander 2016^271^ | Body Dysmorphic Disorder | GAF; CGI-I; EQ-5D |
| Koshikawa 2016^272^ | Schizophrenia | Social Functioning Scale (SFS); University of California San Diego Performance-Based Skills Assessment Brief (UPSA-B), Social Emotional Cognition Task (SECT) |
| Eisendrath 2016^273^ | Depression | CGI |
| Souza 2016^274^ | Depression | WHOQOL-BREF; CGI |
| Buszewicz 2016^275^ | Depression | Euroquol-EQ-VAS; WSAS |
| Meuldijk 2016^276^ | Depression; Anxiety disorder | Brief Symptom Inventory (BSI) and the Web Screening Questionnaire (WSQ); CGI; SF-36 |
| Kampmann 2016^277^ | Anxiety disorder | Fear of Negative Evaluation Scale-Brief Form (FNE-B) |
| Dahlin 2016^278^ | Anxiety disorder | PSWQ Penn State Worry Questionnaire; QOLI |
| Kasper 2016^279^ | Depression; Anxiety disorder | CGI; SDS; SF-36 |
| Lin 2017^280^ | Schizophrenia | GAF |
| Littlewood 2015^281^ | Depression | EQ-5D; SF-36; Clinical Outcomes in Routine Evaluation -Outcome Measure (CORE-OM) questionnaire |
| Steibliene 2016^282^ | Schizophrenia | CGI-S; CGI-I; BPRS |
| McLean 2015^283^ | OCD | Q-LES-Q; Social Adjustment Scale-Self-report (SAS-SR); Brown Assessment of Beliefs Scale (BABS) |
| Bonnin 2016^284^ | Bipolar disorder | FAST |
| Holliday 2015^285^ | PTSD | Quality of Life Inventory QOLI; SF-36 |
| Burton 2016^286^ | Depression | Dysfunctional Attitude Scale - Short Form (DAS-SF); EQ-5D-3L |
| Schreiner 2015^287^ | Schizophrenia | time to relapse; CGI-S; CGI-C; Treatment Satisfaction Questionnaire for Medication; TSQM; SF-36; EQ-5D |
| Morgensterns 2016^288^ | ADHD | Perceived Stress Scale (PSS); SDS; Mindful Attention Awareness Scale (MAAS); Acceptance & Action Questionnaire, 9-item version (AAQ-9); |
| Aljumah 2015^289^ | Depression | EQ-5D |
| Silva 2015^290^ | Schizophrenia | SF-36 |
| Michalak 2015^291^ | Depression | social Adaptation Self-Evaluation Scale (SASS); SF-36 |
| Valenstein 2016^292^ | Depression | Veterans RAND 36-Item Health Survey (VR-36); Q-LES-Q |
| Florea 2015^293^ | Depression | SF-36; EQ-5D; 12-item Health Status Questionnaire |
| Robinson 2015^294^ | Schizophrenia | SCID; CGI |
| Yeh 2015^295^ | Schizophrenia | CGI-S |
| Lopez-Navarro 2015^296^ | Mental disorder | WHOQOL-BREF; Mindfulness Attention Awareness Scale |
| Priebe 2015^297^ | Psychosis | MANSA; Camberwell Assessment of Need Short Appraisal Schedule (CANSAS); CSQ; General Self-efficacy Scale (GSS); Warwick-Edinburgh Mental Well-Being Scale (WEMWBS); Scale for Assessing Therapeutic Relationships in Community Mental Health Care, (STAR-P)(STAR-C); SIX Objective Social Outcomes Index |
| Visser 2015^298^ | OCD | EuroQol |
| Reme 2015^299^ | mental disorder | EQ-5D; work participation |
| Polusny 2015^300^ | PTSD | WHOQOL-BREF; Five Facet Mindfulness Questionnaire (FFMQ); Credibility/Expectancy Questionnaire |
| Loh 2015^301^ | Schizophrenia | global functioning (PSP); SF-36 |
| Naber 2015^302^ | Schizophrenia | QLS; CGI |
| Mauri 2015^303^ | Schizophrenia | CGI-S; Personal and Social Performance (PSP) scale; sleep evaluation scale |
| Martin 2015^304^ | Depression | Australian Quality of Life instrument (AQoL); SF-36 |
| Knekt 2015^305^ | Depression; Anxiety disorder | (Social Adjustment Scale (SAS-SR); (Sense of Coherence Scale (SOC); (Self-Performance Survey); Life Orientation Test (LOT); Life Situation Survey (LSS) |
| Chen 2015^306^ | Depression | WHOQOL-BREF; Occupational self-assessment, the Mastery scale, the Social support questionnaire |
| Schneier 2015^307^ | PTSD | CGI-C; Pittsburgh Sleep Quality Index (PSQI); Q-LES-Q; SF-12 |
| ter Huurne 2015^308^ | Eating disorder | EQ-5D; International Classification of Functioning, Disability and Health (MATE-ICN) |
| Targum 2015^309^ | Depression | Massachusetts General Hospital Cognitive and Physical Functioning Questionnaire (MGH-CPFQ); CGI-S; CGI-I |
| Young 2015^310^ | ADHD | CGI; QOLS |
| Kastner 2015^311^ | Schizophrenia | BPRS; GAF; WODAS-II |
| Schreiner 2015^312^ | Schizophrenia | CGI-S; Social Performance (PSP) ; Mini International Classification of Functionality, Disability and Health (ICF) Rating for Activity and Participation Disorders in Psychological Illnesses (Mini-ICF-APP) scale; Treatment Satisfaction Questionnaire for Medication (TSQM) |
| Reimherr 2015^313^ | ADHD | CGI-I |
| Andersson 2015^314^ | OCD | CGI-I; CGI-S; EQ-5D |
| Mennin 2015^315^ | Depression; Anxiety disorder | Penn State Worry Questionnaire (PSWQ); SDS; Affect Intensity Measure (AIM); Experiences Questionnaire; Difficulties in Emotion Regulation Scale (DERS); Emotion Regulation Questionnaire (ERQ); CGI |
| Mueser 2015^316^ | PTSD | SCID; QOLI; GAF |
| van der Voort 2015^317^ | Bipolar disorder | FAST; WHO-QoL-bref |
| Lepp anen2016^318^ | Borderline Personality Disorder | Health Related Quality of Life (HRQoL) |
| Malchow 2015^319^ | Schizophrenia | GAF; Social Adjustment Scale-II (SAS-II); CGI-S; erbal Learning Memory Test (VLMT); Wisconsin Card Sorting Test (WCST); Trail Making Tests (TMT-a and TMT-B) |
| Martiny 2015^320^ | Depression | WHO (Five) Well-Being Index; GAF; The Preskorn scale |
| Ran 2015^321^ | Schizophrenia | GAF |
| Lavretsky 2015^322^ | Depression | SF-36; CGI-I |
| Ucok 2015^323^ | Schizophrenia | Personal and Social Performance Scale (PSP); GAF |
| Kim 2015^324^ | Depression | WHOQOL-BREF |
| Kilbourne 2015^325^ | Bipolar disorder | Internal State Scale (ISS); SF-12; WODAS-2 |
| Huys 2016^326^ | Tourette Syndrome | GAF; MSQoL, Modular System for Quality of Life; STAI, State-Trait Anxiety Inventory; |
| Wesner 2015^327^ | Panic disorder | Coping Strategies Inventory (CSI); (WHOQOL-Bref) |
| Chiliza 2016^328^ | Schizophrenia | CGI; SOFAS; WHOQOL-BREF |
| Goto 2017^329^ | ADHD | Behavior Rating Inventory of Executive Function-Adult Version: Self-Report (BRIEF-A: Self-Report) |
| Stech 2020^330^ | Panic disorder; Agoraphobia | Work and Social Adjustment Scale (WSAS); Days out of role (DOR) |
| Manolache 2020^331^ | Bipolar disorder; alcohol use disorder | GAF; EQ-5D; CGI-S; 10 point Visual Analogic Scale (VAS) for the treatment tolerability self-evaluation |
| Juretic 2020^332^ | Depression | MANSA |
| Gaudiano 2020^333^ | Psychosis | BPRSClinical Outcomes in Routine Evaluation (CORE); QLS; WODAS-2; Acceptance and Action Questionnaire-II (AAQ-II); Cognitive and Affective Mindfulness Scale-Revised (CAMS-R); Valuing Questionnaire (VQ:; CSQ |
| Norton 2019^334^ | Depression | CGI-I |
| Shafti 2019^335^ | OCD | CGI-S |
| Shah 2018^336^ | Panic disorder | Quality-of-Life Scale |
| Tang 2018^337^ | Schizophrenia | BPRS; Q-LES-Q; Autobiographical Memory Inventory (AMI), Montreal Cognitive Assessment (MoCA), and reorientation time |
| Singh 2018^338^ | Schizophrenia | CGI; GAF |
| Lalande 2017^339^ | Depression; Anxiety disorder | The outcome rating scale (ORS); Toronto mindfulness scale (TMS); GRMT-self-efficacy scale (GRMT-SE); working alliance inventory-short form |
| Glick 2017^340^ | Schizophrenia | Global Outcome Scale [GOS]; Global  Assessment of Functioning Scale [GAFS]); Satisfaction with Life Scale (SLS) |
| Buchanan 2017^341^ | Schizophrenia | Birchwood Social Functioning Scale (BSFS) ; Defeatist Performance Attitude Scale (DPAS) ; BPRS |
| Madrid 2017^342^ | Depression | CGI-I; CGI-S; Facial Expression Recognition Task (FERT) |
| Miller 2019^343^ | Schizophrenia | CogState computerized cognitive battery |
| De la Gandara 2017^344^ | Psychosis | CGI |
| Goud 2017^345^ | Schizophrenia | CGI-I |
| Tomassini 2017^346^ | Depression; Bipolar disorder | CGI-S; BPRS; WHOQOL |
| Salzman 2017^347^ | Bipolar disorder | FAST |
| Guillena 2017^348^ | Depression | SDS |
| Nunes 2017^349^ | Depression | CGI; SDS; WHOQOL-BREF; Structured Clinical Interview, International Physical Activity Questionnaire |
| Fernandez-Miranda 2017^350^ | Bipolar disorder; substance use disorder | CGI; visual Analogue Scale for craving (VASc), the Functional Assessment Staging Test (FAST) and the Quality of Life-Index (QL-INDEX) |
| Cuomo 2017^351^ | Psychosis; substance use disorder | CGI; WHOQOL |
| Anand 2017^352^ | Schizophrenia | CGI |
| Mofsen 2017^353^ | Schizophrenia | CGI-S |
| Zrinzo 2017^354^ | OCD | GAF |
| Voort 2016^355^ | Depression | CGI-S; CGI-C |
| Schlaepfer 2016^356^ | Depression | SF-36; GAF |
| Sturup 2018^357^ | Schizophrenia | social functioning (GSDS (Groningen Social Disabilities Schedule)), recovery, quality of life (WHO-5), client satisfaction (CSQ (Client Satisfaction Questionaire)), side-effects of antipsychotic medication (UKU), sexuality (CSFQ (Changes in Sexual Functioning questionaire)) |
| Jauhar 2019^358^ | Psychosis | CGI |
| Zarranz 2016^359^ | Mental disorder | Personal and Social Performance (PSP) ; Heinrichs-Carpenter of Life Scale (QLS) |
| Waite 2016^360^ | Depression | Q-LES-Q SF |
| Gavrilova 2016^361^ | Depression | CGI |
| Kaltsatou 2015^362^ | Schizophrenia | GAF; Q-LES-Q; functional capacity assessments |

## **3.4 Most prevalent transdiagnostic outcome measures**

**additional table 3: ten most prevalent global outcome measures within the 362 included clinical trials**

| **Outcome measure** | **Frequency** |
| --- | --- |
| Clinical Global Impression | 93 |
| Short Form Health Survey | 66 |
| Global Assessment of Functioning | 60 |
| EuroQol 5d | 55 |
| World Health Organization Quality of Life | 46 |
| Quality of Life, Enjoyment, and Satisfaction Questionnaire | 27 |
| Brief Psychiatric Rating scale | 21 |
| Client’s Satisfaction Questionnaire | 20 |
| Lehman’s 20-item Quality of Life Interview | 16 |
| Social and Occupational Functioning Assessment Scale | 14 |

# **Online supplement section 4: detailed methods and results systematic review aptness of transdiagnostic outcome measure for detecting treatment effects compared to HDRS**

## **4.1 Detailed methods**

The search was limited to Pubmed and Embase; the full search strategies can be found below. Two of the authors removed duplicates and screened the titles and abstracts and subsequently full text of found literature to exclude titles that were not relevant for this analysis. Studies that did not primarily focus on depressive disorder, studies that did not use the HDRS and SF36 and observational studies without an intervention were excluded. For each of the selected studies, the number and description of the participants (comorbidities, in/outpatients), objective of the study, interventions used (anti-depressants, psychotherapy or alternative) and follow-up time in weeks were extracted. The data from the change, baseline and follow up score of the Hamilton and SF36 sub-scores were collected using the mean and standard deviations of the groups, where available. When mean changes in HDRS of SF36 sub scores were unavailable, the baseline and follow up value would be used to calculate the mean change. P-values for a paired t-test for the null hypothesis that treatment effects were equal to 0 were obtained for each outcome measure, for each study, and a confusion matrix was created to investigate the ability of the SF36 to reveal a significant treatment effect.

## **4.2 Search strategies**

### Pubmed search:

(“depressive disorder” [MeSH] OR “Depression” [MeSH] OR “Affective Disorders, Psychotic” [MeSH] OR depress* [Tiab] OR “affective disorder*”[Tiab] ) AND (“SF 36” [Tiab] OR “36-item short form survey*” [Tiab] OR “Short form survey*” [Tiab] OR “Short form score*” [Tiab] OR “short form scale*” [Tiab] OR “short form 36*” [Tiab] OR “short form health*” [Tiab] OR “SF health*” [Tiab] OR “SF questionnaire” [Tiab] OR “short form questionnaire*” [Tiab]) AND (“Hamilton depression rating score” [Tiab] OR “HAM D” [Tiab] OR (“HAMD” [Tiab] AND “depression” [Tiab]) OR “hamilton score”[tiab] OR “HRDS” [Tiab] OR “HDRS” [Tiab] OR “HRSD” [Tiab] OR “Hamilton rating score for depression” [Tiab] OR “Hamilton depression rating*” [Tiab] OR “hamilton rating*” [Tiab] OR “HAMD17” [Tiab] OR “17-Item Hamilton Depression Rating Scale” [Tiab] OR "hamilton depress*" [Tiab] )

### Embase search:

(depressive disorder’ OR ‘Depression’ ti:ab OR ‘Affective Disorders, Psychotic’ ti:ab OR ‘depress’ ti:ab OR ‘affective disorder ‘:ti,ab ) AND (‘SF 36’ :ti,ab OR ‘36-item short form survey ‘ :ti,ab OR ‘Short form survey ‘ :ti,ab OR ‘Short form score ‘ :ti,ab OR ‘short form scale ‘ :ti,ab OR ‘short form 36 ‘ :ti,ab OR ‘short form health ‘ :ti,ab OR ‘SF health ‘ :ti,ab OR ‘SF questionnaire’ :ti,ab OR ‘short form questionnaire ‘ :ti,ab) AND (‘Hamilton depression rating score’ :ti,ab OR ‘HAM D’ :ti,ab OR (‘HAMD’ :ti,ab AND ‘depression’ :ti,ab) OR ‘hamilton score’:ti,ab OR ‘HRDS’ :ti,ab OR ‘HDRS’ :ti,ab OR ‘HRSD’ :ti,ab OR ‘Hamilton rating score for depression’ :ti,ab OR ‘Hamilton depression rating ‘ :ti,ab OR ‘hamilton rating ‘ :ti,ab OR ‘HAMD17’ :ti,ab OR ‘17-Item Hamilton Depression Rating Scale’ :ti,ab OR ‘hamilton depress ‘ :ti,ab )

## **4.3 Overview included studies**

**additional table 4: clinical trials in psychiatry that both used the HDRS and the SF36 as outcome measures. For HDRS and SF36 scores in each intervention group, see the additional excel file (additional information 2).**

| **Study ID** | **Follow up (weeks)** | **intervention type** | **specification of**  **intervention** | **Patient type** | **group size**  **(n= …)** |
| --- | --- | --- | --- | --- | --- |
| Philipp 1999^363^ | 8 | control | placebo | outpatients | 47 |
|  |  | Alternative  treatment | hyperemicum |  | 106 |
|  |  | anti-depressants | imipramine |  | 110 |
| Fortner  1999^364^ | 12 | intervention | buproprion | outpatients | 18 |
| Gleason  2002^365^ | 8 | intervention | citalopram | outpatients | 15 |
| Ceroni  2002^366^ | 52 | intervention | experimental case program | outpatients | 80 |
| Miner  2002^367^ | 12 | intervention | citalopram | outpatients | 81 |
|  |  | intervention | paroxetine |  | 72 |
|  |  | intervention | sertraline |  | 82 |
| Marangell  2002^368^ | 52 | intervention | vagus nerve stimulation | outpatients | 30 |
| Gask 2004^369^ | 52 | intervention | educational intervention  GP | outpatients | 92 |
| Trick 2004^370^ | 26 | intervention | venlafaxine | outpatients | 45 |
|  |  | intervention | dothiepin |  | 43 |
| Gulseren  2005^371^ | 12 | intervention | fluoxetine | outpatients | 11 |
|  |  | intervention | paroxetine |  | 9 |
| Cole 2006^372^ | 26 | intervention | Psychiatrist treatment | inpatients | 33 |
|  |  | control | usual geriatric care |  | 31 |
| Wise 2007^373^ | 8 | intervention | duloxetine | outpatients | 52 |
|  |  | control | placebo |  | 26 |
| Rollmann  2009^374^ | 32 | intervention | telephone delivered care | outpatients | 150 |
|  |  | control | usual care |  | 152 |
| Kendrick  2009^375^ | 26 | intervention | SSRI | outpatients | 112 |
|  |  | control | supportive care |  | 108 |
| Brothers  2011^376^ | 26 | intervention | exercise and cognitive intervention | outpatients | 36 |
| Han  2012^377^ | 8 | intervention | GGBT | outpatients | 82 |
|  |  | control | placebo |  | 82 |
| Bewernick  2012^378^ | 104 | intervention | deep brain stimulation | outpatients | 11 |
| Cao  2013^379^ | 6 | intervention | fluoxetine | outpatients | 103 |
| Zhang  2016^380^ | 52 | intervention | CGT | outpatients | 32 |
|  |  | control | usual care |  | 30 |
| Shinmei  2016^381^ | 12 | intervention | CGT | outpatients | 18 |
| Huang  2017^382^ | 12 | intervention | ECT | inpatients | 95 |
| Yang 2017^383^ | 6 | intervention | fluoxetine | inpatients | 106 |
| Jiang  2018^384^ | 12 | intervention | EPA_DHA | outpatients | 28 |
|  |  | intervention | high_EPA |  | 24 |
|  |  | control | placebo |  | 28 |
| Trottier-Duclos  2018^385^ | 12 | intervention | vagus nerve stimulation | outpatients | 20 |
| Deslandes  2010^386^ | 52 | intervention | exercise and cognitive intervention | outpatients | 10 |
|  |  | control | pharmacological |  | 10 |
| Revicki  1998^387^ | 26 | control | subMRT | outpatients | 163 |
|  |  | intervention | MRT |  | 195 |
| Thompson  2000^388^ | 12 | intervention | fluoxetine | outpatients | 73 |
|  |  | intervention | dothiepin |  | 72 |

##

## **4.4 Detailed results**

**additional table 5: Correlation coefficients of the HDRS and SF36 subscores.**

| **SF36 subscore** | **Correlation coefficient** |
| --- | --- |
| physical health | -0.786 |
| role perception physical | -0.717 |
| bodily pain | -0.601 |
| general health perception | -0.730 |
| mental health | -0.709 |
| role perception emotional | -0.623 |
| vitality | -0.623 |
| social functioning | -0.610 |

**additional table 6: Specificity, sensitivity and positive predictive value of the SF36 subcomponents for severity of depression, as compared to the HDRS.**

| **SF36 subcomponent** | **Specificity** | **Sensitivity** | **Positive Predictive Value** |
| --- | --- | --- | --- |
| Physical health | 0.667 | 0.550 | 0.846 |
| Role Perception Physical | 0.667 | 0.706 | 0.857 |
| Bodily pain | 0.667 | 0.421 | 0.800 |
| General Health Perception | 0.667 | 0.588 | 0.833 |
| Mental Health | 0.286 | 0.850 | 0.773 |
| Role Perception Emotional | 0.286 | 0.882 | 0.750 |
| Vitality | 0.500 | 0.900 | 0.857 |
| Social Functioning | 0.667 | 0.750 | 0.882 |

# **Online supplement section 5: detailed results for quantitative assessment of feasibility of extracting information on outcome measure themes from the EHR**

**additional table 7: EHR sourced assessed for extracting information on each outcome measure theme.**

| **Source** | **Theme(s)*** | **Structured** | **Availability** | **Relevance** | **Quality** |
| --- | --- | --- | --- | --- | --- |
| Clinical notes | 1, 2, 3, 4 | No | High, 98-100% | High | High |
| Correspondence | 1, 2 | No | High, 87% | High | Medium |
| Kennedy Axis V questionnaire | 1, 2 | Mixed | Low, 21% | High | Medium |
| Crisis prevention plan | 1 | Mixed | Low, 1% | Medium | Low |
| Global Assessment of Functioning (GAF) end score | 1, 2 | Yes | High, 75% | Medium | Medium |
| Juridical status | 1, 2 | Yes | High, 100% | High | High |
| Destination after dismissal | 1, 2 | Yes | High, 100% | Medium | Medium |
| Medication use | 1 | Yes | High, 93-100% | Medium | Medium |
| Positive health questionnaire  end score | 4 | Yes | Low, 17% | High | High |
| Lab measurements | 1 | Yes | High, 83% | Low | High |

**Note: theme 1 = symptom reduction, 2 = social functioning, 3 = general well-being, 4 = patient's*
*experience.*

## **References for the Systematic Reviews**

1. Monfort Montolio M, Sancho-Pelluz J. Animal-Assisted Therapy in the Residential Treatment of Dual Pathology. *Int J Environ Res Public Health.* 2019;17(1).

2. Crowe M, Inder M, Douglas K, et al. Interpersonal and Social Rhythm Therapy for Patients With Major Depressive Disorder. *Am J Psychother.* 2020;73(1):29-34.

3. Anjara SG, Bonetto C, Ganguli P, et al. Can General Practitioners manage mental disorders in primary care? A partially randomised, pragmatic, cluster trial. *PLoS One.* 2019;14(11):e0224724.

4. Murata T, Hiramatsu Y, Yamada F, et al. Alterations of mental defeat and cognitive flexibility during cognitive behavioral therapy in patients with major depressive disorder: a single-arm pilot study. *BMC Res Notes.* 2019;12(1):723.

5. Veselinovic T, Scharpenberg M, Heinze M, et al. Dopamine D2 Receptor Occupancy Estimated From Plasma Concentrations of Four Different Antipsychotics and the Subjective Experience of Physical and Mental Well-Being in Schizophrenia: Results From the Randomized NeSSy Trial. *J Clin Psychopharmacol.* 2019;39(6):550-560.

6. Lewis G, Duffy L, Ades A, et al. The clinical effectiveness of sertraline in primary care and the role of depression severity and duration (PANDA): a pragmatic, double-blind, placebo-controlled randomised trial. *Lancet Psychiatry.* 2019;6(11):903-914.

7. Aubry T, Bourque J, Goering P, et al. A randomized controlled trial of the effectiveness of Housing First in a small Canadian City. *BMC Public Health.* 2019;19(1):1154.

8. Hahn B, Shrieves ME, Yuille MB, Buchanan RW, Wells AK. Nicotine effects on cognitive remediation training outcome in people with schizophrenia: A pilot study. *Psychiatry Res.* 2019;280:112498.

9. Xie ZJ, Han N, Law S, et al. The efficacy of group cognitive-behavioural therapy plus duloxetine for generalised anxiety disorder versus duloxetine alone. *Acta Neuropsychiatr.* 2019;31(6):316-324.

10. Gilliam FG, Black KJ, Carter J, et al. A Trial of Sertraline or Cognitive Behavior Therapy for Depression in Epilepsy. *Ann Neurol.* 2019;86(4):552-560.

11. Adewuya AO, Ola BA, Coker O, Atilola O, Fasawe A, Ajomale T. A stepped care intervention for non-specialist health workers' management of depression in the Mental Health in Primary Care (MeHPriC) project, Lagos, Nigeria: A cluster randomised controlled trial. *Gen Hosp Psychiatry.* 2019;60:76-82.

12. Zhao B, Li Z, Wang Y, et al. Can acupuncture combined with SSRIs improve clinical symptoms and quality of life in patients with depression? Secondary outcomes of a pragmatic randomized controlled trial. *Complement Ther Med.* 2019;45:295-302.

13. Wesner AC, Behenck A, Finkler D, et al. Resilience and coping strategies in cognitive behavioral group therapy for patients with panic disorder. *Arch Psychiatr Nurs.* 2019;33(4):428-433.

14. Philip NS, Barredo J, Aiken E, et al. Theta-Burst Transcranial Magnetic Stimulation for Posttraumatic Stress Disorder. *Am J Psychiatry.* 2019;176(11):939-948.

15. Hensel JM, Shaw J, Ivers NM, et al. A Web-Based Mental Health Platform for Individuals Seeking Specialized Mental Health Care Services: Multicenter Pragmatic Randomized Controlled Trial. *J Med Internet Res.* 2019;21(6):e10838.

16. Meng FQ, Han HY, Luo J, et al. Efficacy of cognitive behavioural therapy with medication for patients with obsessive-compulsive disorder: A multicentre randomised controlled trial in China. *J Affect Disord.* 2019;253:184-192.

17. Serfaty M, King M, Nazareth I, et al. Manualised cognitive-behavioural therapy in treating depression in advanced cancer: the CanTalk RCT. *Health Technol Assess.* 2019;23(19):1-106.

18. Chan KL, Leung WC, Tiwari A, Or KL, Ip P. Using Smartphone-Based Psychoeducation to Reduce Postnatal Depression Among First-Time Mothers: Randomized Controlled Trial. *JMIR Mhealth Uhealth.* 2019;7(5):e12794.

19. Gomes BC, Rocca CC, Belizario GO, et al. Cognitive behavioral rehabilitation for bipolar disorder patients: A randomized controlled trial. *Bipolar Disord.* 2019;21(7):621-633.

20. Mustafa S, Bougie J, Miguelez M, et al. Real-life assessment of aripiprazole monthly (Abilify Maintena) in schizophrenia: a Canadian naturalistic non-interventional prospective cohort study. *BMC Psychiatry.* 2019;19(1):114.

21. Puspitosari WA, Wardaningsih S, Nanwani S. Improving the quality of life of people with schizophrenia through community based rehabilitation in Yogyakarta Province, Indonesia: A quasi experimental study. *Asian J Psychiatr.* 2019;42:67-73.

22. Geraets CNW, Veling W, Witlox M, Staring ABP, Matthijssen S, Cath D. Virtual reality-based cognitive behavioural therapy for patients with generalized social anxiety disorder: a pilot study. *Behav Cogn Psychother.* 2019;47(6):745-750.

23. Mak AD, Chung VCH, Yuen SY, et al. Noneffectiveness of electroacupuncture for comorbid generalized anxiety disorder and irritable bowel syndrome. *J Gastroenterol Hepatol.* 2019;34(10):1736-1742.

24. Best MW, Milanovic M, Iftene F, Bowie CR. A Randomized Controlled Trial of Executive Functioning Training Compared With Perceptual Training for Schizophrenia Spectrum Disorders: Effects on Neurophysiology, Neurocognition, and Functioning. *Am J Psychiatry.* 2019;176(4):297-306.

25. Gowda SM, Narayanaswamy JC, Hazari N, et al. Efficacy of pre-supplementary motor area transcranial direct current stimulation for treatment resistant obsessive compulsive disorder: A randomized, double blinded, sham controlled trial. *Brain Stimul.* 2019;12(4):922-929.

26. Wilhelm S, Phillips KA, Greenberg JL, et al. Efficacy and Posttreatment Effects of Therapist-Delivered Cognitive Behavioral Therapy vs Supportive Psychotherapy for Adults With Body Dysmorphic Disorder: A Randomized Clinical Trial. *JAMA Psychiatry.* 2019;76(4):363-373.

27. van Emmerik-van Oortmerssen K, Vedel E, Kramer FJ, et al. Integrated cognitive behavioral therapy for ADHD in adult substance use disorder patients: Results of a randomized clinical trial. *Drug Alcohol Depend.* 2019;197:28-36.

28. Jensen SB, Dalum HS, Korsbek L, et al. Illness management and recovery: one-year follow-up of a randomized controlled trial in Danish community mental health centers: long-term effects on clinical and personal recovery. *BMC Psychiatry.* 2019;19(1):65.

29. Bressington D, Mui J, Yu C, et al. Feasibility of a group-based laughter yoga intervention as an adjunctive treatment for residual symptoms of depression, anxiety and stress in people with depression. *J Affect Disord.* 2019;248:42-51.

30. Kahlke F, Berger T, Schulz A, et al. Efficacy of an unguided internet-based self-help intervention for social anxiety disorder in university students: A randomized controlled trial. *Int J Methods Psychiatr Res.* 2019;28(2):e1766.

31. Quilty LC, Allen TA, Davis C, Knyahnytska Y, Kaplan AS. A randomized comparison of long acting methylphenidate and cognitive behavioral therapy in the treatment of binge eating disorder. *Psychiatry Res.* 2019;273:467-474.

32. Berk M, Turner A, Malhi GS, et al. A randomised controlled trial of a mitochondrial therapeutic target for bipolar depression: mitochondrial agents, N-acetylcysteine, and placebo. *BMC Med.* 2019;17(1):18.

33. van Dijk SDM, Veenstra MS, Bouman R, et al. Group schema-focused therapy enriched with psychomotor therapy versus treatment as usual for older adults with cluster B and/or C personality disorders: a randomized trial. *BMC Psychiatry.* 2019;19(1):26.

34. Kilbourne AM, Prenovost KM, Liebrecht C, et al. Randomized Controlled Trial of a Collaborative Care Intervention for Mood Disorders by a National Commercial Health Plan. *Psychiatr Serv.* 2019;70(3):219-224.

35. Cho M, Jang SJ. Effect of an emotion management programme for patients with schizophrenia: A quasi-experimental design. *Int J Ment Health Nurs.* 2019;28(2):592-604.

36. Pos K, Franke N, Smit F, et al. Cognitive behavioral therapy for social activation in recent-onset psychosis: Randomized controlled trial. *J Consult Clin Psychol.* 2019;87(2):151-160.

37. Pezzin LE, Larson ER, Lorber W, McGinley EL, Dillingham TR. Music-instruction intervention for treatment of post-traumatic stress disorder: a randomized pilot study. *BMC Psychol.* 2018;6(1):60.

38. Saglam Aykut D. Comparison of Paliperidone Palmitate and Second-Generation Oral Antipsychotics in Terms of Medication Adherence, Side Effects, and Quality of Life. *J Clin Psychopharmacol.* 2019;39(1):57-62.

39. Jelinek L, Faissner M, Moritz S, Kriston L. Long-term efficacy of Metacognitive Training for Depression (D-MCT): A randomized controlled trial. *Br J Clin Psychol.* 2019;58(3):245-259.

40. Bauer IE, Green C, Colpo GD, et al. A Double-Blind, Randomized, Placebo-Controlled Study of Aspirin and N-Acetylcysteine as Adjunctive Treatments for Bipolar Depression. *J Clin Psychiatry.* 2018;80(1).

41. Vieta E, Earley WR, Burgess MV, et al. Long-term safety and tolerability of cariprazine as adjunctive therapy in major depressive disorder. *Int Clin Psychopharmacol.* 2019;34(2):76-83.

42. Howard R, Cort E, Bradley R, et al. Amisulpride for very late-onset schizophrenia-like psychosis: the ATLAS three-arm RCT. *Health Technol Assess.* 2018;22(67):1-62.

43. Lee DJ, Dallapiazza RF, De Vloo P, et al. Inferior thalamic peduncle deep brain stimulation for treatment-refractory obsessive-compulsive disorder: A phase 1 pilot trial. *Brain Stimul.* 2019;12(2):344-352.

44. Possemato K, Johnson EM, Emery JB, et al. A pilot study comparing peer supported web-based CBT to self-managed web CBT for primary care veterans with PTSD and hazardous alcohol use. *Psychiatr Rehabil J.* 2019;42(3):305-313.

45. Kessler D, Burns A, Tallon D, et al. Combining mirtazapine with SSRIs or SNRIs for treatment-resistant depression: the MIR RCT. *Health Technol Assess.* 2018;22(63):1-136.

46. Kulz AK, Landmann S, Cludius B, et al. Mindfulness-based cognitive therapy (MBCT) in patients with obsessive-compulsive disorder (OCD) and residual symptoms after cognitive behavioral therapy (CBT): a randomized controlled trial. *Eur Arch Psychiatry Clin Neurosci.* 2019;269(2):223-233.

47. Sanz Cruces JM, Garcia Cuenca IM, Lacomba-Trejo L, et al. Group Therapy for Patients with Adjustment Disorder in Primary Care. *Span J Psychol.* 2018;21:E50.

48. Kessler DS, MacNeill SJ, Tallon D, et al. Mirtazapine added to SSRIs or SNRIs for treatment resistant depression in primary care: phase III randomised placebo controlled trial (MIR). *BMJ.* 2018;363:k4218.

49. Muralidharan A, Brown CH, J EP, et al. Living Well: An Intervention to Improve Medical Illness Self-Management Among Individuals With Serious Mental Illness. *Psychiatr Serv.* 2019;70(1):19-25.

50. Blanco C, Markowitz JC, Hellerstein DJ, et al. A randomized trial of interpersonal psychotherapy, problem solving therapy, and supportive therapy for major depressive disorder in women with breast cancer. *Breast Cancer Res Treat.* 2019;173(2):353-364.

51. Rosengard RJ, Malla A, Mustafa S, et al. Association of Pre-onset Subthreshold Psychotic Symptoms With Longitudinal Outcomes During Treatment of a First Episode of Psychosis. *JAMA Psychiatry.* 2019;76(1):61-70.

52. Mahmood Z, Clark JMR, Twamley EW. Compensatory Cognitive Training for psychosis: Effects on negative symptom subdomains. *Schizophr Res.* 2019;204:397-400.

53. Jahoda A, Hastings R, Hatton C, et al. Behavioural activation versus guided self-help for depression in adults with learning disabilities: the BeatIt RCT. *Health Technol Assess.* 2018;22(53):1-130.

54. Sajatovic M, Tatsuoka C, Cassidy KA, et al. A 6-Month, Prospective, Randomized Controlled Trial of Customized Adherence Enhancement Versus Bipolar-Specific Educational Control in Poorly Adherent Individuals With Bipolar Disorder. *J Clin Psychiatry.* 2018;79(6).

55. Halverson T, Jarskog LF, Pedersen C, Penn D. Effects of oxytocin on empathy, introspective accuracy, and social symptoms in schizophrenia: A 12-week twice-daily randomized controlled trial. *Schizophr Res.* 2019;204:178-182.

56. Schennach R, Riedel M, Spellmann I, et al. Comparing Schizophrenia Patients With a Predicted High/Low Risk of Nonresponse Receiving Treatment with Ziprasidone and Haloperidol: A Randomized-Controlled Study. *Pharmacopsychiatry.* 2019;52(4):180-185.

57. Petzold MB, Mumm JLM, Bischoff S, et al. Increasing physical activity and healthy diet in outpatients with mental disorders: a randomized-controlled evaluation of two psychological interventions. *Eur Arch Psychiatry Clin Neurosci.* 2019;269(5):529-542.

58. Petersen JJ, Hartig J, Paulitsch MA, et al. Classes of depression symptom trajectories in patients with major depression receiving a collaborative care intervention. *PLoS One.* 2018;13(9):e0202245.

59. Meltzer-Brody S, Colquhoun H, Riesenberg R, et al. Brexanolone injection in post-partum depression: two multicentre, double-blind, randomised, placebo-controlled, phase 3 trials. *Lancet.* 2018;392(10152):1058-1070.

60. Conway CR, Kumar A, Xiong W, Bunker M, Aaronson ST, Rush AJ. Chronic Vagus Nerve Stimulation Significantly Improves Quality of Life in Treatment-Resistant Major Depression. *J Clin Psychiatry.* 2018;79(5).

61. Stein DJ, Khoo JP, Ahokas A, et al. 12-week double-blind randomized multicenter study of efficacy and safety of agomelatine (25-50mg/day) versus escitalopram (10-20mg/day) in out-patients with severe generalized anxiety disorder. *Eur Neuropsychopharmacol.* 2018;28(8):970-979.

62. Sarris J, Byrne GJ, Bousman C, et al. Adjunctive S-adenosylmethionine (SAMe) in treating non-remittent major depressive disorder: An 8-week double-blind, randomized, controlled trial<sup/>. *Eur Neuropsychopharmacol.* 2018;28(10):1126-1136.

63. Ertekin Pinar S Rn P, Tel H Rn P. The Effect of Music on Auditory Hallucination and Quality of Life in Schizophrenic Patients: A Randomised Controlled Trial. *Issues Ment Health Nurs.* 2019;40(1):50-57.

64. Gao K, Goto T, Yuan C, et al. A Pilot Study of the Effectiveness of Lithium Versus Quetiapine Immediate Release Monotherapy in Patients With Bipolar Spectrum Disorders. *J Clin Psychopharmacol.* 2018;38(5):422-434.

65. Cladder-Micus MB, Speckens AEM, Vrijsen JN, AR TD, Becker ES, Spijker J. Mindfulness-based cognitive therapy for patients with chronic, treatment-resistant depression: A pragmatic randomized controlled trial. *Depress Anxiety.* 2018;35(10):914-924.

66. Kool M, Van HL, Bartak A, et al. Optimizing psychotherapy dosage for comorbid depression and personality disorders (PsyDos): a pragmatic randomized factorial trial using schema therapy and short-term psychodynamic psychotherapy. *BMC Psychiatry.* 2018;18(1):252.

67. Menon V, Selvakumar N, Kattimani S, Andrade C. Therapeutic effects of mobile-based text message reminders for medication adherence in bipolar I disorder: Are they maintained after intervention cessation? *J Psychiatr Res.* 2018;104:163-168.

68. Reme SE, Monstad K, Fyhn T, et al. A randomized controlled multicenter trial of individual placement and support for patients with moderate-to-severe mental illness. *Scand J Work Environ Health.* 2019;45(1):33-41.

69. Tendler A, Gersner R, Roth Y, Zangen A. Alternate day dTMS combined with SSRIs for chronic treatment resistant depression: A prospective multicenter study. *J Affect Disord.* 2018;240:130-136.

70. Geramita EM, Herbeck Belnap B, Abebe KZ, Rothenberger SD, Rotondi AJ, Rollman BL. The Association Between Increased Levels of Patient Engagement With an Internet Support Group and Improved Mental Health Outcomes at 6-Month Follow-Up: Post-Hoc Analyses From a Randomized Controlled Trial. *J Med Internet Res.* 2018;20(7):e10402.

71. Bjork Bramberg E, Holmgren K, Bultmann U, et al. Increasing return-to-work among people on sick leave due to common mental disorders: design of a cluster-randomized controlled trial of a problem-solving intervention versus care-as-usual conducted in the Swedish primary health care system (PROSA). *BMC Public Health.* 2018;18(1):889.

72. Dalton B, Bartholdy S, McClelland J, et al. Randomised controlled feasibility trial of real versus sham repetitive transcranial magnetic stimulation treatment in adults with severe and enduring anorexia nervosa: the TIARA study. *BMJ Open.* 2018;8(7):e021531.

73. Emsley R, Ahokas A, Suarez A, et al. Efficacy of Tianeptine 25-50 mg in Elderly Patients With Recurrent Major Depressive Disorder: An 8-Week Placebo- and Escitalopram-Controlled Study. *J Clin Psychiatry.* 2018;79(4).

74. Lequimener-de Lorgeril V, Chirio-Espitalier M, Grall-Bronnec M. [Influence of a therapeutic patient education (TPE) program on quality of life in bipolar disorder: Preliminary results]. *Encephale.* 2019;45(2):127-132.

75. Luo X, Law SF, Wang X, et al. Effectiveness of an Assertive Community Treatment program for people with severe schizophrenia in mainland China - a 12-month randomized controlled trial. *Psychol Med.* 2019;49(6):969-979.

76. Iglesias-Gonzalez M, Aznar-Lou I, Penarrubia-Maria MT, et al. Effectiveness of watchful waiting versus antidepressants for patients diagnosed of mild to moderate depression in primary care: A 12-month pragmatic clinical trial (INFAP study). *Eur Psychiatry.* 2018;53:66-73.

77. Yesavage JA, Fairchild JK, Mi Z, et al. Effect of Repetitive Transcranial Magnetic Stimulation on Treatment-Resistant Major Depression in US Veterans: A Randomized Clinical Trial. *JAMA Psychiatry.* 2018;75(9):884-893.

78. Schlosser DA, Campellone TR, Truong B, et al. Efficacy of PRIME, a Mobile App Intervention Designed to Improve Motivation in Young People With Schizophrenia. *Schizophr Bull.* 2018;44(5):1010-1020.

79. Delgadillo J, de Jong K, Lucock M, et al. Feedback-informed treatment versus usual psychological treatment for depression and anxiety: a multisite, open-label, cluster randomised controlled trial. *Lancet Psychiatry.* 2018;5(7):564-572.

80. Patel SR, Wheaton MG, Andersson E, et al. Acceptability, Feasibility, and Effectiveness of Internet-Based Cognitive-Behavioral Therapy for Obsessive-Compulsive Disorder in New York. *Behav Ther.* 2018;49(4):631-641.

81. Li S, Blumenthal JA, Shi C, et al. I-CARE randomized clinical trial integrating depression and acute coronary syndrome care in low-resource hospitals in China: Design and rationale. *Am Heart J.* 2018;202:109-115.

82. Gliddon E, Cosgrove V, Berk L, et al. A randomized controlled trial of MoodSwings 2.0: An internet-based self-management program for bipolar disorder. *Bipolar Disord.* 2019;21(1):28-39.

83. Bormann JE, Thorp SR, Smith E, et al. Individual Treatment of Posttraumatic Stress Disorder Using Mantram Repetition: A Randomized Clinical Trial. *Am J Psychiatry.* 2018;175(10):979-988.

84. Turakitwanakan W, Pongpaplud P, Kitporntheranunt M. The Effect of Home Buddhist Mindfulness Meditation on Depressive Symptom in Major Depressive Patients. *J Med Assoc Thai.* 2017;99 Suppl 8:S171-S178.

85. Ben-Zeev D, Brian RM, Jonathan G, et al. Mobile Health (mHealth) Versus Clinic-Based Group Intervention for People With Serious Mental Illness: A Randomized Controlled Trial. *Psychiatr Serv.* 2018;69(9):978-985.

86. El Hage C, Ghabrash MF, Dubreucq S, et al. A pilot, open-label, 8-week study evaluating desvenlafaxine for treatment of major depression in methadone-maintained individuals with opioid use disorder. *Int Clin Psychopharmacol.* 2018;33(5):268-273.

87. Sanches SA, van Busschbach JT, Michon HWC, van Weeghel J, Swildens WE. The Role of Working Alliance in Attainment of Personal Goals and Improvement in Quality of Life During Psychiatric Rehabilitation. *Psychiatr Serv.* 2018;69(8):903-909.

88. Renna ME, Quintero JM, Soffer A, et al. A Pilot Study of Emotion Regulation Therapy for Generalized Anxiety and Depression: Findings From a Diverse Sample of Young Adults. *Behav Ther.* 2018;49(3):403-418.

89. de Jong S, van Donkersgoed RJM, Timmerman ME, et al. Metacognitive reflection and insight therapy (MERIT) for patients with schizophrenia. *Psychol Med.* 2019;49(2):303-313.

90. Kamijima K, Kimura M, Kuwahara K, Kitayama Y, Tadori Y. Randomized, double-blind comparison of aripiprazole/sertraline combination and placebo/sertraline combination in patients with major depressive disorder. *Psychiatry Clin Neurosci.* 2018;72(8):591-601.

91. Samalin L, Honciuc M, Boyer L, et al. Efficacy of shared decision-making on treatment adherence of patients with bipolar disorder: a cluster randomized trial (ShareD-BD). *BMC Psychiatry.* 2018;18(1):103.

92. Nishida A, Ando S, Yamasaki S, et al. A randomized controlled trial of comprehensive early intervention care in patients with first-episode psychosis in Japan: 1.5-year outcomes from the J-CAP study. *J Psychiatr Res.* 2018;102:136-141.

93. Crawford MJ, Sanatinia R, Barrett B, et al. Lamotrigine for people with borderline personality disorder: a RCT. *Health Technol Assess.* 2018;22(17):1-68.

94. Li J, Huang YG, Ran MS, et al. Community-based comprehensive intervention for people with schizophrenia in Guangzhou, China: Effects on clinical symptoms, social functioning, internalized stigma and discrimination. *Asian J Psychiatr.* 2018;34:21-30.

95. Dalum HS, Waldemar AK, Korsbek L, et al. Illness management and recovery: Clinical outcomes of a randomized clinical trial in community mental health centers. *PLoS One.* 2018;13(4):e0194027.

96. Shimada T, Ohori M, Inagaki Y, et al. A multicenter, randomized controlled trial of individualized occupational therapy for patients with schizophrenia in Japan. *PLoS One.* 2018;13(4):e0193869.

97. Porcu M, Urbano MR, Verri WA, Jr., et al. Effects of adjunctive N-acetylcysteine on depressive symptoms: Modulation by baseline high-sensitivity C-reactive protein. *Psychiatry Res.* 2018;263:268-274.

98. Moritz S, Ahlf-Schumacher J, Hottenrott B, et al. We cannot change the past, but we can change its meaning. A randomized controlled trial on the effects of self-help imagery rescripting on depression. *Behav Res Ther.* 2018;104:74-83.

99. Chen R, Zhu X, Capitao LP, et al. Psychoeducation for psychiatric inpatients following remission of a manic episode in bipolar I disorder: A randomized controlled trial. *Bipolar Disord.* 2019;21(1):76-85.

100. JGL AT, Morina N, Topper M, Emmelkamp PMG. A Randomized Controlled Trial in Routine Clinical Practice Comparing Acceptance and Commitment Therapy with Cognitive Behavioral Therapy for the Treatment of Major Depressive Disorder. *Psychother Psychosom.* 2018;87(3):154-163.

101. Hui CLM, Honer WG, Lee EHM, et al. Long-term effects of discontinuation from antipsychotic maintenance following first-episode schizophrenia and related disorders: a 10 year follow-up of a randomised, double-blind trial. *Lancet Psychiatry.* 2018;5(5):432-442.

102. Pena J, Ibarretxe-Bilbao N, Sanchez P, et al. Mechanisms of functional improvement through cognitive rehabilitation in schizophrenia. *J Psychiatr Res.* 2018;101:21-27.

103. Mennin DS, Fresco DM, O'Toole MS, Heimberg RG. A randomized controlled trial of emotion regulation therapy for generalized anxiety disorder with and without co-occurring depression. *J Consult Clin Psychol.* 2018;86(3):268-281.

104. Stiekema APM, Looijmans A, van der Meer L, et al. Effects of a lifestyle intervention on psychosocial well-being of severe mentally ill residential patients: ELIPS, a cluster randomized controlled pragmatic trial. *Schizophr Res.* 2018;199:407-413.

105. Berle D, Hilbrink D, Russell-Williams C, et al. Personal wellbeing in posttraumatic stress disorder (PTSD): association with PTSD symptoms during and following treatment. *BMC Psychol.* 2018;6(1):7.

106. du Sert OP, Potvin S, Lipp O, et al. Virtual reality therapy for refractory auditory verbal hallucinations in schizophrenia: A pilot clinical trial. *Schizophr Res.* 2018;197:176-181.

107. Schottle D, Schimmelmann BG, Ruppelt F, et al. Effectiveness of integrated care including therapeutic assertive community treatment in severe schizophrenia-spectrum and bipolar I disorders: Four-year follow-up of the ACCESS II study. *PLoS One.* 2018;13(2):e0192929.

108. Laurenssen EMP, Luyten P, Kikkert MJ, et al. Day hospital mentalization-based treatment v. specialist treatment as usual in patients with borderline personality disorder: randomized controlled trial. *Psychol Med.* 2018;48(15):2522-2529.

109. Najavits LM, Krinsley K, Waring ME, Gallagher MW, Skidmore C. A Randomized Controlled Trial for Veterans with PTSD and Substance Use Disorder: Creating Change versus Seeking Safety. *Subst Use Misuse.* 2018;53(11):1788-1800.

110. Harned MS, Wilks CR, Schmidt SC, Coyle TN. Improving functional outcomes in women with borderline personality disorder and PTSD by changing PTSD severity and post-traumatic cognitions. *Behav Res Ther.* 2018;103:53-61.

111. Haji Seyed Javadi A, Shafikhani AA, Zamir SM, Khanshir ZF. Evaluation of the Effect of Fluvoxamine in Patients With Schizophrenia Under Risperidone Treatment: A Clinical Trial. *J Clin Psychopharmacol.* 2018;38(2):119-124.

112. Bjorkelund C, Svenningsson I, Hange D, et al. Clinical effectiveness of care managers in collaborative care for patients with depression in Swedish primary health care: a pragmatic cluster randomized controlled trial. *BMC Fam Pract.* 2018;19(1):28.

113. Lin CH, Lin CH, Chang YC, et al. Sodium Benzoate, a D-Amino Acid Oxidase Inhibitor, Added to Clozapine for the Treatment of Schizophrenia: A Randomized, Double-Blind, Placebo-Controlled Trial. *Biol Psychiatry.* 2018;84(6):422-432.

114. O'Haire ME, Rodriguez KE. Preliminary efficacy of service dogs as a complementary treatment for posttraumatic stress disorder in military members and veterans. *J Consult Clin Psychol.* 2018;86(2):179-188.

115. Savitz JB, Teague TK, Misaki M, et al. Treatment of bipolar depression with minocycline and/or aspirin: an adaptive, 2x2 double-blind, randomized, placebo-controlled, phase IIA clinical trial. *Transl Psychiatry.* 2018;8(1):27.

116. Hoxhaj E, Sadohara C, Borel P, et al. Mindfulness vs psychoeducation in adult ADHD: a randomized controlled trial. *Eur Arch Psychiatry Clin Neurosci.* 2018;268(4):321-335.

117. Mergl R, Allgaier AK, Hautzinger M, Coyne JC, Hegerl U, Henkel V. One-year follow-up of a randomized controlled trial of sertraline and cognitive behavior group therapy in depressed primary care patients (MIND study). *J Affect Disord.* 2018;230:15-21.

118. Bryce SD, Rossell SL, Lee SJ, et al. Neurocognitive and Self-efficacy Benefits of Cognitive Remediation in Schizophrenia: A Randomized Controlled Trial. *J Int Neuropsychol Soc.* 2018;24(6):549-562.

119. Aftab A, Levin J, Aebi M, Bhat C, Sajatovic M. Associations of Comorbid Anxiety With Medication Adherence and Psychiatric Symptomatology in a Population of Nonadherent Bipolar Disorder Subjects. *J Nerv Ment Dis.* 2018;206(4):258-262.

120. Gaughran F, Stahl D, Ismail K, et al. Randomised control trial of the effectiveness of an integrated psychosocial health promotion intervention aimed at improving health and reducing substance use in established psychosis (IMPaCT). *BMC Psychiatry.* 2017;17(1):413.

121. Di Lorenzo R, Cameli M, Piemonte C, et al. Clinical improvement, relapse and treatment adherence with paliperidone palmitate 1-month formulation: 1-year treatment in a naturalistic outpatient setting. *Nord J Psychiatry.* 2018;72(3):214-220.

122. Penades R, Lopez-Vilchez I, Catalan R, et al. BDNF as a marker of response to cognitive remediation in patients with schizophrenia: A randomized and controlled trial. *Schizophr Res.* 2018;197:458-464.

123. Inchausti F, Garcia-Poveda NV, Ballesteros-Prados A, et al. The Effects of Metacognition-Oriented Social Skills Training on Psychosocial Outcome in Schizophrenia-Spectrum Disorders: A Randomized Controlled Trial. *Schizophr Bull.* 2018;44(6):1235-1244.

124. Corrigan P, Sheehan L, Morris S, et al. The Impact of a Peer Navigator Program in Addressing the Health Needs of Latinos With Serious Mental Illness. *Psychiatr Serv.* 2018;69(4):456-461.

125. McGuire P, Robson P, Cubala WJ, et al. Cannabidiol (CBD) as an Adjunctive Therapy in Schizophrenia: A Multicenter Randomized Controlled Trial. *Am J Psychiatry.* 2018;175(3):225-231.

126. Sahin Z, Vinnars B, Gorman BS, Wilczek A, Asberg M, Barber JP. Clinical severity as a moderator of outcome in psychodynamic and dialectical behavior therapies for borderline personality disorder. *Personal Disord.* 2018;9(5):437-446.

127. Bitter N, Roeg D, van Assen M, van Nieuwenhuizen C, van Weeghel J. How effective is the comprehensive approach to rehabilitation (CARe) methodology? A cluster randomized controlled trial. *BMC Psychiatry.* 2017;17(1):396.

128. Chang BH, Chen BW, Beckstead JW, Yang CY. Effects of a music-creation programme on the anxiety, self-esteem, and quality of life of people with severe mental illness: A quasi-experimental design. *Int J Ment Health Nurs.* 2018;27(3):1066-1076.

129. Younan R, Farrell J, May T. 'Teaching Me to Parent Myself': The Feasibility of an In-Patient Group Schema Therapy Programme for Complex Trauma. *Behav Cogn Psychother.* 2018;46(4):463-478.

130. Katsumi A, Hoshino H, Fujimoto S, et al. Effects of cognitive remediation on cognitive and social functions in individuals with schizophrenia. *Neuropsychol Rehabil.* 2019;29(9):1475-1487.

131. Calabrese JR, Sanchez R, Jin N, et al. Symptoms and functioning with aripiprazole once-monthly injection as maintenance treatment for bipolar I disorder. *J Affect Disord.* 2018;227:649-656.

132. Bosanquet K, Adamson J, Atherton K, et al. CollAborative care for Screen-Positive EldeRs with major depression (CASPER plus): a multicentred randomised controlled trial of clinical effectiveness and cost-effectiveness. *Health Technol Assess.* 2017;21(67):1-252.

133. Berger T, Krieger T, Sude K, Meyer B, Maercker A. Evaluating an e-mental health program ("deprexis") as adjunctive treatment tool in psychotherapy for depression: Results of a pragmatic randomized controlled trial. *J Affect Disord.* 2018;227:455-462.

134. Goldstein LA, Mehling WE, Metzler TJ, et al. Veterans Group Exercise: A randomized pilot trial of an Integrative Exercise program for veterans with posttraumatic stress. *J Affect Disord.* 2018;227:345-352.

135. Shimizu N, Umemura T, Matsunaga M, Hirai T. An interactive sports video game as an intervention for rehabilitation of community-living patients with schizophrenia: A controlled, single-blind, crossover study. *PLoS One.* 2017;12(11):e0187480.

136. Huang HC, Liu SI, Hwang LC, et al. The effectiveness of Culturally Sensitive Collaborative Treatment of depressed Chinese in family medicine clinics: A randomized controlled trial. *Gen Hosp Psychiatry.* 2018;50:96-103.

137. Sibeko G, Temmingh H, Mall S, et al. Improving adherence in mental health service users with severe mental illness in South Africa: a pilot randomized controlled trial of a treatment partner and text message intervention vs. treatment as usual. *BMC Res Notes.* 2017;10(1):584.

138. Eklund M, Tjornstrand C, Sandlund M, Argentzell E. Effectiveness of Balancing Everyday Life (BEL) versus standard occupational therapy for activity engagement and functioning among people with mental illness - a cluster RCT study. *BMC Psychiatry.* 2017;17(1):363.

139. Rollman BL, Herbeck Belnap B, Abebe KZ, et al. Effectiveness of Online Collaborative Care for Treating Mood and Anxiety Disorders in Primary Care: A Randomized Clinical Trial. *JAMA Psychiatry.* 2018;75(1):56-64.

140. Cohen JN, Drabick DAG, Blanco C, Schneier FR, Liebowitz MR, Heimberg RG. Pharmacotherapy for social anxiety disorder: Interpersonal predictors of outcome and the mediating role of the working alliance. *J Anxiety Disord.* 2017;52:79-87.

141. Azevedo da Silva R, de Azevedo Cardoso T, Campos Mondin T, et al. Is Narrative Cognitive Therapy as Effective as Cognitive Behavior Therapy in the Treatment for Depression in Young Adults? *J Nerv Ment Dis.* 2017;205(12):918-924.

142. Girgis RR, Ciarleglio A, Choo T, et al. A Randomized, Double-Blind, Placebo-Controlled Clinical Trial of Tocilizumab, An Interleukin-6 Receptor Antibody, For Residual Symptoms in Schizophrenia. *Neuropsychopharmacology.* 2018;43(6):1317-1323.

143. Kivelitz L, Kriston L, Christalle E, et al. Effectiveness of telephone-based aftercare case management for adult patients with unipolar depression compared to usual care: A randomized controlled trial. *PLoS One.* 2017;12(10):e0186967.

144. Castillo EG, Shaner R, Tang L, et al. Improving Depression Care for Adults With Serious Mental Illness in Underresourced Areas: Community Coalitions Versus Technical Support. *Psychiatr Serv.* 2018;69(2):195-203.

145. Ruesch M, Helmes A, Bengel J. Cognitive behavioral group therapy for patients with physical diseases and comorbid depressive or adjustment disorders on a waiting list for individual therapy: results from a randomized controlled trial. *BMC Psychiatry.* 2017;17(1):340.

146. Gordon A, Davis PJ, Patterson S, et al. A randomized waitlist control community study of Social Cognition and Interaction Training for people with schizophrenia. *Br J Clin Psychol.* 2018;57(1):116-130.

147. Contreras NA, Tan EJ, Lee SJ, Castle DJ, Rossell SL. Using visual processing training to enhance standard cognitive remediation outcomes in schizophrenia: A pilot study. *Psychiatry Res.* 2018;262:494-499.

148. Schjerning O, Damkier P, Lykkegaard SE, Jakobsen KD, Nielsen J. Pregabalin for anxiety in patients with schizophrenia - A randomized, double-blind placebo-controlled study. *Schizophr Res.* 2018;195:260-266.

149. Buchheim A, Horz-Sagstetter S, Doering S, et al. Change of Unresolved Attachment in Borderline Personality Disorder: RCT Study of Transference-Focused Psychotherapy. *Psychother Psychosom.* 2017;86(5):314-316.

150. Vergunst F, Rugkasa J, Koshiaris C, Simon J, Burns T. Community treatment orders and social outcomes for patients with psychosis: a 48-month follow-up study. *Soc Psychiatry Psychiatr Epidemiol.* 2017;52(11):1375-1384.

151. Husain MI, Chaudhry IB, Husain N, et al. Minocycline as an adjunct for treatment-resistant depressive symptoms: A pilot randomised placebo-controlled trial. *J Psychopharmacol.* 2017;31(9):1166-1175.

152. Richards DA, Rhodes S, Ekers D, et al. Cost and Outcome of BehaviouRal Activation (COBRA): a randomised controlled trial of behavioural activation versus cognitive-behavioural therapy for depression. *Health Technol Assess.* 2017;21(46):1-366.

153. Lima AF, Miguel SR, Cohen M, et al. Effectiveness evaluation of mood disorder treatment algorithms in Brazilian public healthcare patients. *Braz J Psychiatry.* 2018;40(1):26-34.

154. Baruah U, Pandian RD, Narayanaswamy JC, Bada Math S, Kandavel T, Reddy YCJ. A randomized controlled study of brief family-based intervention in obsessive compulsive disorder. *J Affect Disord.* 2018;225:137-146.

155. Gershkovich M, Herbert JD, Forman EM, Schumacher LM, Fischer LE. Internet-Delivered Acceptance-Based Cognitive-Behavioral Intervention for Social Anxiety Disorder With and Without Therapist Support: A Randomized Trial. *Behav Modif.* 2017;41(5):583-608.

156. de Zwaan M, Herpertz S, Zipfel S, et al. Effect of Internet-Based Guided Self-help vs Individual Face-to-Face Treatment on Full or Subsyndromal Binge Eating Disorder in Overweight or Obese Patients: The INTERBED Randomized Clinical Trial. *JAMA Psychiatry.* 2017;74(10):987-995.

157. Bozzatello P, Rocca P, Uscinska M, Bellino S. Efficacy and Tolerability of Asenapine Compared with Olanzapine in Borderline Personality Disorder: An Open-Label Randomized Controlled Trial. *CNS Drugs.* 2017;31(9):809-819.

158. Ong MK, Jones L, Aoki W, et al. A Community-Partnered, Participatory, Cluster-Randomized Study of Depression Care Quality Improvement: Three-Year Outcomes. *Psychiatr Serv.* 2017;68(12):1262-1270.

159. Valimaki M, Kannisto KA, Vahlberg T, Hatonen H, Adams CE. Short Text Messages to Encourage Adherence to Medication and Follow-up for People With Psychosis (Mobile.Net): Randomized Controlled Trial in Finland. *J Med Internet Res.* 2017;19(7):e245.

160. Khan MN, Hamdani SU, Chiumento A, et al. Evaluating feasibility and acceptability of a group WHO trans-diagnostic intervention for women with common mental disorders in rural Pakistan: a cluster randomised controlled feasibility trial. *Epidemiol Psychiatr Sci.* 2019;28(1):77-87.

161. Zoellner LA, Telch M, Foa EB, et al. Enhancing Extinction Learning in Posttraumatic Stress Disorder With Brief Daily Imaginal Exposure and Methylene Blue: A Randomized Controlled Trial. *J Clin Psychiatry.* 2017;78(7):e782-e789.

162. Wessels H, Wagner M, Kuhr K, et al. Predictors of treatment response to psychological interventions in people at clinical high risk of first-episode psychosis. *Early Interv Psychiatry.* 2019;13(1):120-127.

163. Lovell K, Bower P, Gellatly J, et al. Low-intensity cognitive-behaviour therapy interventions for obsessive-compulsive disorder compared to waiting list for therapist-led cognitive-behaviour therapy: 3-arm randomised controlled trial of clinical effectiveness. *PLoS Med.* 2017;14(6):e1002337.

164. Gabbay MB, Ring A, Byng R, et al. Debt Counselling for Depression in Primary Care: an adaptive randomised controlled pilot trial (DeCoDer study). *Health Technol Assess.* 2017;21(35):1-164.

165. Biesheuvel-Leliefeld KEM, Dijkstra-Kersten SMA, van Schaik DJF, et al. Effectiveness of Supported Self-Help in Recurrent Depression: A Randomized Controlled Trial in Primary Care. *Psychother Psychosom.* 2017;86(4):220-230.

166. Driessen E, Van HL, Peen J, et al. Cognitive-behavioral versus psychodynamic therapy for major depression: Secondary outcomes of a randomized clinical trial. *J Consult Clin Psychol.* 2017;85(7):653-663.

167. Citrome L, Risinger R, Cutler AJ, et al. Effect of aripiprazole lauroxil in patients with acute schizophrenia as assessed by the Positive and Negative Syndrome Scale-supportive analyses from a Phase 3 study. *CNS Spectr.* 2018;23(4):284-290.

168. Watanabe K, Thase ME, Kikuchi T, et al. Long-term function and psychosocial outcomes with venlafaxine extended release 75-225 mg/day versus placebo in the PREVENT study. *Int Clin Psychopharmacol.* 2017;32(5):271-280.

169. Eriksson MCM, Kivi M, Hange D, et al. Long-term effects of Internet-delivered cognitive behavioral therapy for depression in primary care - the PRIM-NET controlled trial. *Scand J Prim Health Care.* 2017;35(2):126-136.

170. Dean OM, Kanchanatawan B, Ashton M, et al. Adjunctive minocycline treatment for major depressive disorder: A proof of concept trial. *Aust N Z J Psychiatry.* 2017;51(8):829-840.

171. Hellstrom L, Bech P, Hjorthoj C, Nordentoft M, Lindschou J, Eplov LF. Effect on return to work or education of Individual Placement and Support modified for people with mood and anxiety disorders: results of a randomised clinical trial. *Occup Environ Med.* 2017;74(10):717-725.

172. Sajatovic M, Gunzler DD, Kanuch SW, et al. A 60-Week Prospective RCT of a Self-Management Intervention for Individuals With Serious Mental Illness and Diabetes Mellitus. *Psychiatr Serv.* 2017;68(9):883-890.

173. Hasan A, Musleh M. The impact of an empowerment intervention on people with schizophrenia: Results of a randomized controlled trial. *Int J Soc Psychiatry.* 2017;63(3):212-223.

174. Videler AC, van Alphen SPJ, van Royen RJJ, van der Feltz-Cornelis CM, Rossi G, Arntz A. Schema therapy for personality disorders in older adults: a multiple-baseline study. *Aging Ment Health.* 2018;22(6):738-747.

175. Hansson L, Lexen A, Holmen J. The effectiveness of narrative enhancement and cognitive therapy: a randomized controlled study of a self-stigma intervention. *Soc Psychiatry Psychiatr Epidemiol.* 2017;52(11):1415-1423.

176. Moreno-Alcazar A, Radua J, Landin-Romero R, et al. Eye movement desensitization and reprocessing therapy versus supportive therapy in affective relapse prevention in bipolar patients with a history of trauma: study protocol for a randomized controlled trial. *Trials.* 2017;18(1):160.

177. Knaevelsrud C, Bottche M, Pietrzak RH, Freyberger HJ, Kuwert P. Efficacy and Feasibility of a Therapist-Guided Internet-Based Intervention for Older Persons with Childhood Traumatization: A Randomized Controlled Trial. *Am J Geriatr Psychiatry.* 2017;25(8):878-888.

178. Mahlke CI, Priebe S, Heumann K, Daubmann A, Wegscheider K, Bock T. Effectiveness of one-to-one peer support for patients with severe mental illness - a randomised controlled trial. *Eur Psychiatry.* 2017;42:103-110.

179. Kendrick T, Stuart B, Leydon GM, et al. Patient-reported outcome measures for monitoring primary care patients with depression: PROMDEP feasibility randomised trial. *BMJ Open.* 2017;7(3):e015266.

180. Ngai FW, Wong PW, Chung KF, Leung KY. The effect of a telephone-based cognitive behavioral therapy on quality of life: a randomized controlled trial. *Arch Womens Ment Health.* 2017;20(3):421-426.

181. Kang SG, Chee IS, Lee K, Lee J. rs7968606 polymorphism of ANKS1B is associated with improvement in the PANSS general score of schizophrenia caused by amisulpride. *Hum Psychopharmacol.* 2017;32(2).

182. Schafer I, Chuey-Ferrer L, Hofmann A, Lieberman P, Mainusch G, Lotzin A. Effectiveness of EMDR in patients with substance use disorder and comorbid PTSD: study protocol for a randomized controlled trial. *BMC Psychiatry.* 2017;17(1):95.

183. Si T, Wang G, Yang F, et al. Efficacy and safety of escitalopram in treatment of severe depression in Chinese population. *Metab Brain Dis.* 2017;32(3):891-901.

184. Awan NR, Jehangir SF, Irfan M, Naeem F, Farooq S. Explanatory model of illness of the patients with schizophrenia and the role of educational intervention. *Schizophr Res.* 2017;190:68-73.

185. Wiltink J, Ruckes C, Hoyer J, et al. Transfer of manualized Short Term Psychodynamic Psychotherapy (STPP) for social anxiety disorder into clinical practice: results from a cluster-randomised controlled trial. *BMC Psychiatry.* 2017;17(1):92.

186. Kloep ML, Hunter RH, Kertz SJ. Examining the effects of a novel training program and use of psychiatric service dogs for military-related PTSD and associated symptoms. *Am J Orthopsychiatry.* 2017;87(4):425-433.

187. Sankhe A, Dalal K, Save D, Sarve P. Evaluation of the effect of Spiritual care on patients with generalized anxiety and depression: a randomized controlled study. *Psychol Health Med.* 2017;22(10):1186-1191.

188. Ishoy PL, Fagerlund B, Broberg BV, et al. No cognitive-enhancing effect of GLP-1 receptor agonism in antipsychotic-treated, obese patients with schizophrenia. *Acta Psychiatr Scand.* 2017;136(1):52-62.

189. Bewernick BH, Kayser S, Gippert SM, Switala C, Coenen VA, Schlaepfer TE. Deep brain stimulation to the medial forebrain bundle for depression- long-term outcomes and a novel data analysis strategy. *Brain Stimul.* 2017;10(3):664-671.

190. Berk M, Daglas R, Dandash O, et al. Quetiapine v. lithium in the maintenance phase following a first episode of mania: randomised controlled trial. *Br J Psychiatry.* 2017;210(6):413-421.

191. de Jong M, Peeters F, Gard T, et al. A Randomized Controlled Pilot Study on Mindfulness-Based Cognitive Therapy for Unipolar Depression in Patients With Chronic Pain. *J Clin Psychiatry.* 2018;79(1).

192. Bambling M, Edwards SC, Hall S, Vitetta L. A combination of probiotics and magnesium orotate attenuate depression in a small SSRI resistant cohort: an intestinal anti-inflammatory response is suggested. *Inflammopharmacology.* 2017;25(2):271-274.

193. Schramm E, Kriston L, Zobel I, et al. Effect of Disorder-Specific vs Nonspecific Psychotherapy for Chronic Depression: A Randomized Clinical Trial. *JAMA Psychiatry.* 2017;74(3):233-242.

194. Morozova M, Burminskiy D, Rupchev G, et al. 5-HT6 Receptor Antagonist as an Adjunct Treatment Targeting Residual Symptoms in Patients With Schizophrenia: Unexpected Sex-Related Effects (Double-Blind Placebo-Controlled Trial). *J Clin Psychopharmacol.* 2017;37(2):169-175.

195. Lin CH, Wang FC, Lin SC, Huang YH, Chen CC. A randomized, double-blind, comparison of the efficacy and safety of low-dose olanzapine plus low-dose trifluoperazine versus full-dose olanzapine in the acute treatment of schizophrenia. *Schizophr Res.* 2017;185:80-87.

196. Tomba E, Tecuta L, Schumann R, Ballardini D. Does psychological well-being change following treatment? An exploratory study on outpatients with eating disorders. *Compr Psychiatry.* 2017;74:61-69.

197. Wikberg C, Westman J, Petersson EL, et al. Use of a self-rating scale to monitor depression severity in recurrent GP consultations in primary care - does it really make a difference? A randomised controlled study. *BMC Fam Pract.* 2017;18(1):6.

198. Corrigan PW, Kraus DJ, Pickett SA, et al. Using Peer Navigators to Address the Integrated Health Care Needs of Homeless African Americans With Serious Mental Illness. *Psychiatr Serv.* 2017;68(3):264-270.

199. Theleritis C, Sakkas P, Paparrigopoulos T, et al. Two Versus One High-Frequency Repetitive Transcranial Magnetic Stimulation Session per Day for Treatment-Resistant Depression: A Randomized Sham-Controlled Trial. *J ECT.* 2017;33(3):190-197.

200. Kwong VW, Chang WC, Chan GH, et al. Clinical and treatment-related determinants of subjective quality of life in patients with first-episode psychosis. *Psychiatry Res.* 2017;249:39-45.

201. Tan CHS, Ishak RB, Lim TXG, Marimuthusamy P, Kaurss K, Leong JJ. Illness management and recovery program for mental health problems: reducing symptoms and increasing social functioning. *J Clin Nurs.* 2017;26(21-22):3471-3485.

202. Cha DS, Best MW, Bowie CR, et al. A randomized, double-blind, placebo-controlled, crossover trial evaluating the effect of intranasal insulin on cognition and mood in individuals with treatment-resistant major depressive disorder. *J Affect Disord.* 2017;210:57-65.

203. Lee SY, Wang TY, Chen SL, et al. The correlation between plasma brain-derived neurotrophic factor and cognitive function in bipolar disorder is modulated by the BDNF Val66Met polymorphism. *Sci Rep.* 2016;6:37950.

204. Gumley A, White R, Briggs A, et al. A parallel group randomised open blinded evaluation of Acceptance and Commitment Therapy for depression after psychosis: Pilot trial outcomes (ADAPT). *Schizophr Res.* 2017;183:143-150.

205. de Roten Y, Ambresin G, Herrera F, et al. Efficacy of an adjunctive brief psychodynamic psychotherapy to usual inpatient treatment of depression: Results of a randomized controlled trial. *J Affect Disord.* 2017;209:105-113.

206. Abramovitch A, Hallion LS, Reese HE, et al. Neurocognitive predictors of treatment response to randomized treatment in adults with tic disorders. *Prog Neuropsychopharmacol Biol Psychiatry.* 2017;74:9-14.

207. Briest J, Bethge M. [The impact of catastrophizing on the effect of depression on pain and functional ability : A longitudinal mediator analysis]. *Schmerz.* 2017;31(2):159-166.

208. Robinson P, Hellier J, Barrett B, et al. The NOURISHED randomised controlled trial comparing mentalisation-based treatment for eating disorders (MBT-ED) with specialist supportive clinical management (SSCM-ED) for patients with eating disorders and symptoms of borderline personality disorder. *Trials.* 2016;17(1):549.

209. Chen EY, Cacioppo J, Fettich K, et al. An adaptive randomized trial of dialectical behavior therapy and cognitive behavior therapy for binge-eating. *Psychol Med.* 2017;47(4):703-717.

210. Lagomasino IT, Dwight-Johnson M, Green JM, et al. Effectiveness of Collaborative Care for Depression in Public-Sector Primary Care Clinics Serving Latinos. *Psychiatr Serv.* 2017;68(4):353-359.

211. Asnaani A, Kaczkurkin AN, Alpert E, McLean CP, Simpson HB, Foa EB. The effect of treatment on quality of life and functioning in OCD. *Compr Psychiatry.* 2017;73:7-14.

212. Mansson KNT, Salami A, Carlbring P, Boraxbekk CJ, Andersson G, Furmark T. Structural but not functional neuroplasticity one year after effective cognitive behaviour therapy for social anxiety disorder. *Behav Brain Res.* 2017;318:45-51.

213. O'Donnell CP, Allott KA, Murphy BP, et al. Adjunctive Taurine in First-Episode Psychosis: A Phase 2, Double-Blind, Randomized, Placebo-Controlled Study. *J Clin Psychiatry.* 2016;77(12):e1610-e1617.

214. Egede LE, Acierno R, Knapp RG, Walker RJ, Payne EH, Frueh BC. Psychotherapy for Depression in Older Veterans Via Telemedicine: Effect on Quality of Life, Satisfaction, Treatment Credibility, and Service Delivery Perception. *J Clin Psychiatry.* 2016;77(12):1704-1711.

215. Stern A, Malik E, Pollak Y, Bonne O, Maeir A. The Efficacy of Computerized Cognitive Training in Adults With ADHD: A Randomized Controlled Trial. *J Atten Disord.* 2016;20(12):991-1003.

216. Sanacora G, Johnson MR, Khan A, et al. Adjunctive Lanicemine (AZD6765) in Patients with Major Depressive Disorder and History of Inadequate Response to Antidepressants: A Randomized, Placebo-Controlled Study. *Neuropsychopharmacology.* 2017;42(4):844-853.

217. Kang R, Wu Y, Li Z, et al. Effect of Community-Based Social Skills Training and Tai-Chi Exercise on Outcomes in Patients with Chronic Schizophrenia: A Randomized, One-Year Study. *Psychopathology.* 2016;49(5):345-355.

218. Dunayevich E, Buchanan RW, Chen CY, et al. Efficacy and safety of the glycine transporter type-1 inhibitor AMG 747 for the treatment of negative symptoms associated with schizophrenia. *Schizophr Res.* 2017;182:90-97.

219. Chen JA, Shapero BG, Trinh NT, et al. Association Between Stigma and Depression Outcomes Among Chinese Immigrants in a Primary Care Setting. *J Clin Psychiatry.* 2016;77(10):e1287-e1292.

220. Arnedt JT, Swanson LM, Dopp RR, et al. Effects of Restricted Time in Bed on Antidepressant Treatment Response: A Randomized Controlled Trial. *J Clin Psychiatry.* 2016;77(10):e1218-e1225.

221. Stergiopoulos V, Gozdzik A, Misir V, et al. The effectiveness of a Housing First adaptation for ethnic minority groups: findings of a pragmatic randomized controlled trial. *BMC Public Health.* 2016;16(1):1110.

222. Mora E, Portella MJ, Forcada I, Vieta E, Mur M. A preliminary longitudinal study on the cognitive and functional outcome of bipolar excellent lithium responders. *Compr Psychiatry.* 2016;71:25-32.

223. Danovitch I, Steiner AJ, Kazdan A, et al. Analysis of Patient-reported Outcomes of Quality of Life and Functioning Before and After Treatment of Major Depressive Disorder Comorbid With Alcohol Use Disorders. *J Addict Med.* 2017;11(1):47-54.

224. Young S, Emilsson B, Sigurdsson JF, et al. A randomized controlled trial reporting functional outcomes of cognitive-behavioural therapy in medication-treated adults with ADHD and comorbid psychopathology. *Eur Arch Psychiatry Clin Neurosci.* 2017;267(3):267-276.

225. Shimada T, Nishi A, Yoshida T, Tanaka S, Kobayashi M. Development of an Individualized Occupational Therapy Programme and its Effects on the Neurocognition, Symptoms and Social Functioning of Patients with Schizophrenia. *Occup Ther Int.* 2016;23(4):425-435.

226. Rosen CS, Azevedo KJ, Tiet QQ, et al. An RCT of Effects of Telephone Care Management on Treatment Adherence and Clinical Outcomes Among Veterans With PTSD. *Psychiatr Serv.* 2017;68(2):151-158.

227. Eustis EH, Hayes-Skelton SA, Roemer L, Orsillo SM. Reductions in experiential avoidance as a mediator of change in symptom outcome and quality of life in acceptance-based behavior therapy and applied relaxation for generalized anxiety disorder. *Behav Res Ther.* 2016;87:188-195.

228. Seki Y, Nagata S, Shibuya T, et al. A feasibility study of the clinical effectiveness and cost-effectiveness of individual cognitive behavioral therapy for panic disorder in a Japanese clinical setting: an uncontrolled pilot study. *BMC Res Notes.* 2016;9(1):458.

229. Rollman BL, Belnap BH, Mazumdar S, et al. Telephone-Delivered Stepped Collaborative Care for Treating Anxiety in Primary Care: A Randomized Controlled Trial. *J Gen Intern Med.* 2017;32(3):245-255.

230. Berger T, Urech A, Krieger T, et al. Effects of a transdiagnostic unguided Internet intervention ('velibra') for anxiety disorders in primary care: results of a randomized controlled trial. *Psychol Med.* 2017;47(1):67-80.

231. Pankowski S, Adler M, Andersson G, Lindefors N, Svanborg C. Group acceptance and commitment therapy (ACT) for bipolar disorder and co-existing anxiety - an open pilot study. *Cogn Behav Ther.* 2017;46(2):114-128.

232. Rossell SL, Francis PS, Galletly C, et al. N-acetylcysteine (NAC) in schizophrenia resistant to clozapine: a double blind randomised placebo controlled trial targeting negative symptoms. *BMC Psychiatry.* 2016;16(1):320.

233. Fiszdon JM, Choi KH, Bell MD, Choi J, Silverstein SM. Cognitive remediation for individuals with psychosis: efficacy and mechanisms of treatment effects. *Psychol Med.* 2016;46(16):3275-3289.

234. Yeung A, Martinson MA, Baer L, et al. The Effectiveness of Telepsychiatry-Based Culturally Sensitive Collaborative Treatment for Depressed Chinese American Immigrants: A Randomized Controlled Trial. *J Clin Psychiatry.* 2016;77(8):e996-e1002.

235. Ngo VK, Sherbourne C, Chung B, et al. Community Engagement Compared With Technical Assistance to Disseminate Depression Care Among Low-Income, Minority Women: A Randomized Controlled Effectiveness Study. *Am J Public Health.* 2016;106(10):1833-1841.

236. Kidd SA, Kerman N, Ernest D, et al. A pilot study of a family cognitive adaptation training guide for individuals with schizophrenia. *Psychiatr Rehabil J.* 2018;41(2):109-117.

237. Goodman DW, Starr HL, Ma YW, Rostain AL, Ascher S, Armstrong RB. Randomized, 6-Week, Placebo-Controlled Study of Treatment for Adult Attention-Deficit/Hyperactivity Disorder: Individualized Dosing of Osmotic-Release Oral System (OROS) Methylphenidate With a Goal of Symptom Remission. *J Clin Psychiatry.* 2017;78(1):105-114.

238. Richards DA, Ekers D, McMillan D, et al. Cost and Outcome of Behavioural Activation versus Cognitive Behavioural Therapy for Depression (COBRA): a randomised, controlled, non-inferiority trial. *Lancet.* 2016;388(10047):871-880.

239. Aardoom JJ, Dingemans AE, van Ginkel JR, Spinhoven P, Van Furth EF, Van den Akker-van Marle ME. Cost-utility of an internet-based intervention with or without therapist support in comparison with a waiting list for individuals with eating disorder symptoms: a randomized controlled trial. *Int J Eat Disord.* 2016;49(12):1068-1076.

240. Steuwe C, Rullkotter N, Ertl V, et al. Effectiveness and feasibility of Narrative Exposure Therapy (NET) in patients with borderline personality disorder and posttraumatic stress disorder - a pilot study. *BMC Psychiatry.* 2016;16:254.

241. McMurran M, Crawford MJ, Reilly J, et al. Psychoeducation with problem-solving (PEPS) therapy for adults with personality disorder: a pragmatic randomised controlled trial to determine the clinical effectiveness and cost-effectiveness of a manualised intervention to improve social functioning. *Health Technol Assess.* 2016;20(52):1-250.

242. Ritzert TR, Forsyth JP, Sheppard SC, Boswell JF, Berghoff CR, Eifert GH. Evaluating the Effectiveness of ACT for Anxiety Disorders in a Self-Help Context: Outcomes From a Randomized Wait-List Controlled Trial. *Behav Ther.* 2016;47(4):444-459.

243. Muntingh A, Laheij M, Sinnema H, et al. [Quality of life and symptoms in patients with chronic depression and anxiety after a self-management training: a randomised controlled trial]. *Tijdschr Psychiatr.* 2016;58(7):504-512.

244. Zoun MH, Koekkoek B, Sinnema H, et al. Effectiveness and cost-effectiveness of a self-management training for patients with chronic and treatment resistant anxiety or depressive disorders: design of a multicenter randomized controlled trial. *BMC Psychiatry.* 2016;16:216.

245. Gingnell M, Frick A, Engman J, et al. Combining escitalopram and cognitive-behavioural therapy for social anxiety disorder: randomised controlled fMRI trial. *Br J Psychiatry.* 2016;209(3):229-235.

246. Otto MW, Pollack MH, Dowd SM, et al. Randomized Trial of D-Cycloserine Enhancement of Cognitive-Behavioral Therapy for Panic Disorder. *Depress Anxiety.* 2016;33(8):737-745.

247. Engel CC, Jaycox LH, Freed MC, et al. Centrally Assisted Collaborative Telecare for Posttraumatic Stress Disorder and Depression Among Military Personnel Attending Primary Care: A Randomized Clinical Trial. *JAMA Intern Med.* 2016;176(7):948-956.

248. Juretic TG, Ruzic K, Letica-Crepulja M, Petric D, Dadic-Hero E, Franciskovic T. Effects of Psychosocial Day Care Programme on Quality of Life in Patients Affected with Schizophrenia - a Prospective Study. *Psychiatr Danub.* 2016;28(2):111-117.

249. Rakitzi S, Georgila P, Efthimiou K, Mueller DR. Efficacy and feasibility of the Integrated Psychological Therapy for outpatients with schizophrenia in Greece: Final results of a RCT. *Psychiatry Res.* 2016;242:137-143.

250. Grunder G, Heinze M, Cordes J, et al. Effects of first-generation antipsychotics versus second-generation antipsychotics on quality of life in schizophrenia: a double-blind, randomised study. *Lancet Psychiatry.* 2016;3(8):717-729.

251. Atiwannapat P, Thaipisuttikul P, Poopityastaporn P, Katekaew W. Active versus receptive group music therapy for major depressive disorder-A pilot study. *Complement Ther Med.* 2016;26:141-145.

252. Yoon S, Ryu JK, Kim CH, et al. Preliminary Effectiveness and Sustainability of Group Aerobic Exercise Program in Patients with Schizophrenia. *J Nerv Ment Dis.* 2016;204(9):644-650.

253. Rajagopalan K, Bacci ED, Ng-Mak D, Wyrwich K, Pikalov A, Loebel A. Effects on health-related quality of life in patients treated with lurasidone for bipolar depression: results from two placebo controlled bipolar depression trials. *BMC Psychiatry.* 2016;16:157.

254. Gelkopf M, Lapid L, Werbeloff N, et al. A strengths-based case management service for people with serious mental illness in Israel: A randomized controlled trial. *Psychiatry Res.* 2016;241:182-189.

255. Schaap GM, Chakhssi F, Westerhof GJ. Inpatient schema therapy for nonresponsive patients with personality pathology: Changes in symptomatic distress, schemas, schema modes, coping styles, experienced parenting styles, and mental well-being. *Psychotherapy (Chic).* 2016;53(4):402-412.

256. Ito M, Horikoshi M, Kato N, et al. Transdiagnostic and Transcultural: Pilot Study of Unified Protocol for Depressive and Anxiety Disorders in Japan. *Behav Ther.* 2016;47(3):416-430.

257. Bozzatello P, Bellino S. Combined therapy with interpersonal psychotherapy adapted for borderline personality disorder: A two-years follow-up. *Psychiatry Res.* 2016;240:151-156.

258. Berry K, Gregg L, Lobban F, Barrowclough C. Therapeutic alliance in psychological therapy for people with recent onset psychosis who use cannabis. *Compr Psychiatry.* 2016;67:73-80.

259. Barnes TR, Leeson VC, Paton C, et al. Antidepressant Controlled Trial For Negative Symptoms In Schizophrenia (ACTIONS): a double-blind, placebo-controlled, randomised clinical trial. *Health Technol Assess.* 2016;20(29):1-46.

260. Makkos A, Pal E, Aschermann Z, et al. High-Frequency Repetitive Transcranial Magnetic Stimulation Can Improve Depression in Parkinson's Disease: A Randomized, Double-Blind, Placebo-Controlled Study. *Neuropsychobiology.* 2016;73(3):169-177.

261. Isitt JJ, Nadipelli VR, Kouassi A, Fava M, Heidbreder C. Health-related quality of life in acute schizophrenia patients treated with RBP-7000 once monthly risperidone: An 8-week, randomized, double-blind, placebo-controlled, multicenter phase 3 study. *Schizophr Res.* 2016;174(1-3):126-131.

262. Chang WC, Kwong VW, Chan GH, et al. Prediction of functional remission in first-episode psychosis: 12-month follow-up of the randomized-controlled trial on extended early intervention in Hong Kong. *Schizophr Res.* 2016;173(1-2):79-83.

263. Richards DA, Bower P, Chew-Graham C, et al. Clinical effectiveness and cost-effectiveness of collaborative care for depression in UK primary care (CADET): a cluster randomised controlled trial. *Health Technol Assess.* 2016;20(14):1-192.

264. Dagani J, Sisti D, Abelli M, et al. Do we need oxytocin to treat schizophrenia? A randomized clinical trial. *Schizophr Res.* 2016;172(1-3):158-164.

265. Priebe S, Savill M, Wykes T, et al. Clinical effectiveness and cost-effectiveness of body psychotherapy in the treatment of negative symptoms of schizophrenia: a multicentre randomised controlled trial. *Health Technol Assess.* 2016;20(11):vii-xxiii, 1-100.

266. Omer S, Golden E, Priebe S. Exploring the Mechanisms of a Patient-Centred Assessment with a Solution Focused Approach (DIALOG+) in the Community Treatment of Patients with Psychosis: A Process Evaluation within a Cluster-Randomised Controlled Trial. *PLoS One.* 2016;11(2):e0148415.

267. Huijbers MJ, Spinhoven P, van Schaik DJ, Nolen WA, Speckens AE. Patients with a preference for medication do equally well in mindfulness-based cognitive therapy for recurrent depression as those preferring mindfulness. *J Affect Disord.* 2016;195:32-39.

268. Meredith LS, Eisenman DP, Han B, et al. Impact of Collaborative Care for Underserved Patients with PTSD in Primary Care: a Randomized Controlled Trial. *J Gen Intern Med.* 2016;31(5):509-517.

269. Wong SY, Yip BH, Mak WW, et al. Mindfulness-based cognitive therapy v. group psychoeducation for people with generalised anxiety disorder: randomised controlled trial. *Br J Psychiatry.* 2016;209(1):68-75.

270. Nierenberg AA, McElroy SL, Friedman ES, et al. Bipolar CHOICE (Clinical Health Outcomes Initiative in Comparative Effectiveness): a pragmatic 6-month trial of lithium versus quetiapine for bipolar disorder. *J Clin Psychiatry.* 2016;77(1):90-99.

271. Enander J, Andersson E, Mataix-Cols D, et al. Therapist guided internet based cognitive behavioural therapy for body dysmorphic disorder: single blind randomised controlled trial. *BMJ.* 2016;352:i241.

272. Koshikawa Y, Takekita Y, Kato M, et al. The Comparative Effects of Risperidone Long-Acting Injection and Paliperidone Palmitate on Social Functioning in Schizophrenia: A 6-Month, Open-Label, Randomized Controlled Pilot Trial. *Neuropsychobiology.* 2016;73(1):35-42.

273. Eisendrath SJ, Gillung E, Delucchi KL, et al. A Randomized Controlled Trial of Mindfulness-Based Cognitive Therapy for Treatment-Resistant Depression. *Psychother Psychosom.* 2016;85(2):99-110.

274. Souza LH, Salum GA, Mosqueiro BP, Caldieraro MA, Guerra TA, Fleck MP. Interpersonal psychotherapy as add-on for treatment-resistant depression: A pragmatic randomized controlled trial. *J Affect Disord.* 2016;193:373-380.

275. Buszewicz M, Griffin M, McMahon EM, Walters K, King M. Practice nurse-led proactive care for chronic depression in primary care: a randomised controlled trial. *Br J Psychiatry.* 2016;208(4):374-380.

276. Meuldijk D, Carlier IV, van Vliet IM, et al. The clinical effectiveness of concise cognitive behavioral therapy with or without pharmacotherapy for depressive and anxiety disorders; a pragmatic randomized controlled equivalence trial in clinical practice. *Contemp Clin Trials.* 2016;47:131-138.

277. Kampmann IL, Emmelkamp PM, Hartanto D, Brinkman WP, Zijlstra BJ, Morina N. Exposure to virtual social interactions in the treatment of social anxiety disorder: A randomized controlled trial. *Behav Res Ther.* 2016;77:147-156.

278. Dahlin M, Andersson G, Magnusson K, et al. Internet-delivered acceptance-based behaviour therapy for generalized anxiety disorder: A randomized controlled trial. *Behav Res Ther.* 2016;77:86-95.

279. Kasper S, Volz HP, Dienel A, Schlafke S. Efficacy of Silexan in mixed anxiety-depression--A randomized, placebo-controlled trial. *Eur Neuropsychopharmacol.* 2016;26(2):331-340.

280. Lin CY, Liang SY, Chang YC, et al. Adjunctive sarcosine plus benzoate improved cognitive function in chronic schizophrenia patients with constant clinical symptoms: A randomised, double-blind, placebo-controlled trial. *World J Biol Psychiatry.* 2017;18(5):357-368.

281. Littlewood E, Duarte A, Hewitt C, et al. A randomised controlled trial of computerised cognitive behaviour therapy for the treatment of depression in primary care: the Randomised Evaluation of the Effectiveness and Acceptability of Computerised Therapy (REEACT) trial. *Health Technol Assess.* 2015;19(101):viii, xxi-171.

282. Steibliene V, Bunevicius A, Savickas A, Prange AJ, Jr., Nemeroff CB, Bunevicius R. Triiodothyronine accelerates and enhances the antipsychotic effect of risperidone in acute schizophrenia. *J Psychiatr Res.* 2016;73:9-16.

283. McLean CP, Zandberg LJ, Van Meter PE, Carpenter JK, Simpson HB, Foa EB. Exposure and response prevention helps adults with obsessive-compulsive disorder who do not respond to pharmacological augmentation strategies. *J Clin Psychiatry.* 2015;76(12):1653-1657.

284. Bonnin CM, Torrent C, Arango C, et al. Functional remediation in bipolar disorder: 1-year follow-up of neurocognitive and functional outcome. *Br J Psychiatry.* 2016;208(1):87-93.

285. Holliday R, Williams R, Bird J, Mullen K, Suris A. The role of cognitive processing therapy in improving psychosocial functioning, health, and quality of life in veterans with military sexual trauma-related posttraumatic stress disorder. *Psychol Serv.* 2015;12(4):428-434.

286. Burton C, Szentagotai Tatar A, McKinstry B, et al. Pilot randomised controlled trial of Help4Mood, an embodied virtual agent-based system to support treatment of depression. *J Telemed Telecare.* 2016;22(6):348-355.

287. Schreiner A, Aadamsoo K, Altamura AC, et al. Paliperidone palmitate versus oral antipsychotics in recently diagnosed schizophrenia. *Schizophr Res.* 2015;169(1-3):393-399.

288. Morgensterns E, Alfredsson J, Hirvikoski T. Structured skills training for adults with ADHD in an outpatient psychiatric context: an open feasibility trial. *Atten Defic Hyperact Disord.* 2016;8(2):101-111.

289. Aljumah K, Hassali MA. Impact of pharmacist intervention on adherence and measurable patient outcomes among depressed patients: a randomised controlled study. *BMC Psychiatry.* 2015;15:219.

290. Silva BA, Cassilhas RC, Attux C, et al. A 20-week program of resistance or concurrent exercise improves symptoms of schizophrenia: results of a blind, randomized controlled trial. *Braz J Psychiatry.* 2015;37(4):271-279.

291. Michalak J, Schultze M, Heidenreich T, Schramm E. A randomized controlled trial on the efficacy of mindfulness-based cognitive therapy and a group version of cognitive behavioral analysis system of psychotherapy for chronically depressed patients. *J Consult Clin Psychol.* 2015;83(5):951-963.

292. Valenstein M, Pfeiffer PN, Brandfon S, et al. Augmenting Ongoing Depression Care With a Mutual Peer Support Intervention Versus Self-Help Materials Alone: A Randomized Trial. *Psychiatr Serv.* 2016;67(2):236-239.

293. Florea I, Danchenko N, Brignone M, Loft H, Rive B, Abetz-Webb L. The effect of vortioxetine on health-related quality of life in patients with major depressive disorder. *Clin Ther.* 2015;37(10):2309-2323 e2306.

294. Robinson DG, Gallego JA, John M, et al. A Randomized Comparison of Aripiprazole and Risperidone for the Acute Treatment of First-Episode Schizophrenia and Related Disorders: 3-Month Outcomes. *Schizophr Bull.* 2015;41(6):1227-1236.

295. Yeh EC, Huang MC, Tsai CJ, Chen CT, Chen KY, Chiu CC. Early treatment response predicted subsequent clinical response in patients with schizophrenia taking paliperidone extended-release. *Psychiatry Res.* 2015;230(1):13-18.

296. Lopez-Navarro E, Del Canto C, Belber M, et al. Mindfulness improves psychological quality of life in community-based patients with severe mental health problems: A pilot randomized clinical trial. *Schizophr Res.* 2015;168(1-2):530-536.

297. Priebe S, Kelley L, Omer S, et al. The Effectiveness of a Patient-Centred Assessment with a Solution-Focused Approach (DIALOG+) for Patients with Psychosis: A Pragmatic Cluster-Randomised Controlled Trial in Community Care. *Psychother Psychosom.* 2015;84(5):304-313.

298. Visser HA, van Megen H, van Oppen P, et al. Inference-Based Approach versus Cognitive Behavioral Therapy in the Treatment of Obsessive-Compulsive Disorder with Poor Insight: A 24-Session Randomized Controlled Trial. *Psychother Psychosom.* 2015;84(5):284-293.

299. Reme SE, Grasdal AL, Lovvik C, Lie SA, Overland S. Work-focused cognitive-behavioural therapy and individual job support to increase work participation in common mental disorders: a randomised controlled multicentre trial. *Occup Environ Med.* 2015;72(10):745-752.

300. Polusny MA, Erbes CR, Thuras P, et al. Mindfulness-Based Stress Reduction for Posttraumatic Stress Disorder Among Veterans: A Randomized Clinical Trial. *JAMA.* 2015;314(5):456-465.

301. Loh SY, Abdullah A, Abu Bakar AK, Thambu M, Nik Jaafar NR. Structured Walking and Chronic Institutionalized Schizophrenia Inmates: A pilot RCT Study on Quality of Life. *Glob J Health Sci.* 2015;8(1):238-248.

302. Naber D, Hansen K, Forray C, et al. Qualify: a randomized head-to-head study of aripiprazole once-monthly and paliperidone palmitate in the treatment of schizophrenia. *Schizophr Res.* 2015;168(1-2):498-504.

303. Mauri M, Mauri MC, Adami M, Reggiardo G, Giulio C. Efficacy and tolerability of paliperidone ER in patients with unsatisfactorily controlled schizophrenia by other antipsychotics: a flexible-dose approach. *Int Clin Psychopharmacol.* 2015;30(6):329-337.

304. Martin PR, Aiello R, Gilson K, Meadows G, Milgrom J, Reece J. Cognitive behavior therapy for comorbid migraine and/or tension-type headache and major depressive disorder: An exploratory randomized controlled trial. *Behav Res Ther.* 2015;73:8-18.

305. Knekt P, Heinonen E, Harkapaa K, et al. Randomized trial on the effectiveness of long- and short-term psychotherapy on psychosocial functioning and quality of life during a 5-year follow-up. *Psychiatry Res.* 2015;229(1-2):381-388.

306. Chen YL, Pan AW, Hsiung PC, et al. Life Adaptation Skills Training (LAST) for persons with depression: A randomized controlled study. *J Affect Disord.* 2015;185:108-114.

307. Schneier FR, Campeas R, Carcamo J, et al. Combined Mirtazapine and Ssri Treatment of Ptsd: A Placebo-Controlled Trial. *Depress Anxiety.* 2015;32(8):570-579.

308. ter Huurne ED, de Haan HA, Postel MG, van der Palen J, VanDerNagel JE, DeJong CA. Web-Based Cognitive Behavioral Therapy for Female Patients With Eating Disorders: Randomized Controlled Trial. *J Med Internet Res.* 2015;17(6):e152.

309. Targum SD, Wedel PC, Fava M. Changes in cognitive symptoms after a buspirone-melatonin combination treatment for Major Depressive Disorder. *J Psychiatr Res.* 2015;68:392-396.

310. Young S, Khondoker M, Emilsson B, et al. Cognitive-behavioural therapy in medication-treated adults with attention-deficit/hyperactivity disorder and co-morbid psychopathology: a randomized controlled trial using multi-level analysis. *Psychol Med.* 2015;45(13):2793-2804.

311. Kastner D, Buchtemann D, Warnke I, et al. Clinical and functional outcome of assertive outreach for patients with schizophrenic disorder: Results of a quasi-experimental controlled trial. *Eur Psychiatry.* 2015;30(6):736-742.

312. Schreiner A, Bergmans P, Cherubin P, et al. Paliperidone palmitate in non-acute patients with schizophrenia previously unsuccessfully treated with risperidone long-acting therapy or frequently used conventional depot antipsychotics. *J Psychopharmacol.* 2015;29(8):910-922.

313. Reimherr FW, Marchant BK, Gift TE, Steans TA, Wender PH. Types of adult attention-deficit hyperactivity disorder (ADHD): baseline characteristics, initial response, and long-term response to treatment with methylphenidate. *Atten Defic Hyperact Disord.* 2015;7(2):115-128.

314. Andersson E, Hedman E, Enander J, et al. D-Cycloserine vs Placebo as Adjunct to Cognitive Behavioral Therapy for Obsessive-Compulsive Disorder and Interaction With Antidepressants: A Randomized Clinical Trial. *JAMA Psychiatry.* 2015;72(7):659-667.

315. Mennin DS, Fresco DM, Ritter M, Heimberg RG. An Open Trial of Emotion Regulation Therapy for Generalized Anxiety Disorder and Cooccurring Depression. *Depress Anxiety.* 2015;32(8):614-623.

316. Mueser KT, Gottlieb JD, Xie H, et al. Evaluation of cognitive restructuring for post-traumatic stress disorder in people with severe mental illness. *Br J Psychiatry.* 2015;206(6):501-508.

317. van der Voort TY, van Meijel B, Hoogendoorn AW, Goossens PJ, Beekman AT, Kupka RW. Collaborative care for patients with bipolar disorder: Effects on functioning and quality of life. *J Affect Disord.* 2015;179:14-22.

318. Leppanen V, Hakko H, Sintonen H, Lindeman S. Comparing Effectiveness of Treatments for Borderline Personality Disorder in Communal Mental Health Care: The Oulu BPD Study. *Community Ment Health J.* 2016;52(2):216-227.

319. Malchow B, Keller K, Hasan A, et al. Effects of Endurance Training Combined With Cognitive Remediation on Everyday Functioning, Symptoms, and Cognition in Multiepisode Schizophrenia Patients. *Schizophr Bull.* 2015;41(4):847-858.

320. Martiny K, Refsgaard E, Lund V, et al. Maintained superiority of chronotherapeutics vs. exercise in a 20-week randomized follow-up trial in major depression. *Acta Psychiatr Scand.* 2015;131(6):446-457.

321. Ran MS, Chan CL, Ng SM, Guo LT, Xiang MZ. The effectiveness of psychoeducational family intervention for patients with schizophrenia in a 14-year follow-up study in a Chinese rural area. *Psychol Med.* 2015;45(10):2197-2204.

322. Lavretsky H, Reinlieb M, St Cyr N, Siddarth P, Ercoli LM, Senturk D. Citalopram, methylphenidate, or their combination in geriatric depression: a randomized, double-blind, placebo-controlled trial. *Am J Psychiatry.* 2015;172(6):561-569.

323. Ucok A, Saka MC, Bilici M. Effects of paliperidone extended release on functioning level and symptoms of patients with recent onset schizophrenia: An open-label, single-arm, flexible-dose, 12-months follow-up study. *Nord J Psychiatry.* 2015;69(6):426-432.

324. Kim JM, Stewart R, Bae KY, et al. Effects of depression co-morbidity and treatment on quality of life in patients with acute coronary syndrome: the Korean depression in ACS (K-DEPACS) and the escitalopram for depression in ACS (EsDEPACS) study. *Psychol Med.* 2015;45(8):1641-1652.

325. Kilbourne AM, Goodrich DE, Nord KM, et al. Long-Term Clinical Outcomes from a Randomized Controlled Trial of Two Implementation Strategies to Promote Collaborative Care Attendance in Community Practices. *Adm Policy Ment Health.* 2015;42(5):642-653.

326. Huys D, Bartsch C, Koester P, et al. Motor Improvement and Emotional Stabilization in Patients With Tourette Syndrome After Deep Brain Stimulation of the Ventral Anterior and Ventrolateral Motor Part of the Thalamus. *Biol Psychiatry.* 2016;79(5):392-401.

327. Wesner AC, Gomes JB, Detzel T, Guimaraes LS, Heldt E. Booster Sessions after Cognitive-Behavioural Group Therapy for Panic Disorder: Impact on Resilience, Coping, and Quality of Life. *Behav Cogn Psychother.* 2015;43(5):513-525.

328. Chiliza B, Ojagbemi A, Esan O, et al. Combining depot antipsychotic with an assertive monitoring programme for treating first-episode schizophrenia in a resource-constrained setting. *Early Interv Psychiatry.* 2016;10(1):54-62.

329. Goto T, Hirata Y, Takita Y, et al. Efficacy and Safety of Atomoxetine Hydrochloride in Asian Adults With ADHD. *J Atten Disord.* 2017;21(2):100-109.

330. Stech EP, Grierson AB, Chen AZ, Sharrock MJ, Mahoney AEJ, Newby JM. Intensive one-week internet-delivered cognitive behavioral therapy for panic disorder and agoraphobia: A pilot study. *Internet Interv.* 2020;20:100315.

331. Manolache R, Alexandru I, Morariu E, Ghenoiu I, Lecu R. P. 313 Effectiveness of combined opioid antagonist and moodstabilizer treatment in bipolar depression and alcohol use disorder. *Eur Neuropsychopharm.* 2020;31:S56.

332. Juretic TG, Letica-Crepulja M, Stevanovic A, Ruzic K, Roncevic-Grzeta I, Dosen A. The effects of psychosocial day care program on clinical symptoms and quality of life of persons with depression: a prospective study. *Eur J Psychiat.* 2020;34(1):27-35.

333. Gaudiano BA, Ellenberg S, Ostrove B, et al. Feasibility and Preliminary Effects of Implementing Acceptance and Commitment Therapy for Inpatients With Psychotic-Spectrum Disorders in a Clinical Psychiatric Intensive Care Setting. *J Cogn Psychother.* 2020;34(1):80-96.

334. Norton J, Fredericks D, Alva G, et al. Open-Label Study of Pimavanserin Patients With Comorbid Parkinson's Disease and Depression. *Movement Disord.* 2019;34:S68-S69.

335. Shafti SS, Kaviani H. Evaluation of Second Generation Antipsychotics, as Augmentative Plan, in Treatment-Resistant Obsessive-Compulsive Disorder. *Current Psychopharmacology.* 2019;8(2):146-154.

336. Shah A, Northcutt J. An open-label, flexible dose adaptive study evaluating the efficacy of vortioxetine in subjects with panic disorder. *Ann Gen Psychiatr.* 2018;17.

337. Tang VM, Blumberger DM, McClintock SM, et al. Magnetic Seizure Therapy in Treatment-resistant Schizophrenia: a Pilot Study. *Front Psychiatry.* 2018;8.

338. Singh A, Beniwal RP, Kukshal P, Bhatia T, Thelma BK, Deshpande SN. A preliminary study of association of genetic variants with early response to olanzapine in schizophrenia. *Indian J Psychiat.* 2018;60(1):10-16.

339. Lalande L, King R, Bambling M, Schweitzer RD. An Uncontrolled Clinical Trial of Guided Respiration Mindfulness Therapy (GRMT) in the Treatment of Depression and Anxiety. *J Contemp Psychother.* 2017;47(4):251-258.

340. Glick I, Davis J. Should Antipsychotic Medications for Schizophrenia be Given for a Lifetime? Replication of a Naturalistic, Long-Term, Follow-Up Study. *Neuropsychopharmacology.* 2017;42:S616-S617.

341. Buchanan R, Kelly DL, Strauss GP, et al. Combined Oxytocin and CBSST for Social Function in People With Schizophrenia. *Neuropsychopharmacology.* 2017;42:S436-S437.

342. Madrid A, Smith D, Alvarez-Horine S, Saljooqi K, Dagum P, Mahableshwarkar A. Assessing Anhedonia With Quantitative Tasks, Digital and Patient Reported Measures in a Multi-Center, Double-Blind Trial With BTRX-246040 for the Treatment of Major Depressive Disorder. *Neuropsychopharmacology.* 2017;42:S372-S372.

343. Miller BJ, Pikalov A, Siu C, et al. Inflammatory Markers and Cognitive Performance in Patients with Schizophrenia Treated with Lurasidone. *Aust Nz J Psychiat.* 2019;53:144-145.

344. De la Gandara JJ, Mayo S. Maintenance to win: prospective observational study of extended-release injectable aripiprazole for maintenance therapy in psychotic outpatients. *Eur Neuropsychopharm.* 2017;27:S892-S893.

345. Goud AC, Shewade DG, Kumar RR, Surendiran A. Effect of dopamine D2 receptor and serotonin 2A receptor gene polymorphisms on response to risperidone in schizophrenia. *Eur Neuropsychopharm.* 2017;27:S958-S959.

346. Tomassini L, Perrini F, Cuomo I, Motta P, Amici E, De Filippis S. Efficacy, safety, and tolerability of vortioxetine in the treatment of mood disorders. *Eur Neuropsychopharm.* 2017;27:S863-S864.

347. Salzman P, Calabrese JR, Sanchez R, et al. Symptoms and functioning in aripiprazole once-monthly maintenance treatment of bipolar I disorder: a blinded, placebo-controlled, randomised study. *Eur Neuropsychopharm.* 2017;27:S830-S830.

348. Guillena SLR, de Diego BOPG, Navarro R, Santamaria O, Sanchez-Luengo FG. Effectiveness and tolerability of vortioxetine for major depressive disorder. *Eur Neuropsychopharm.* 2017;27:S847-S848.

349. Nunes SOV, Porcu M, Vargas HO, Urbano M. N-acetylcysteine as an adjunctive treatment to reduce depressive symptom severity when C-reactive protein levels are increased. *Eur Neuropsychopharm.* 2017;27:S788-S788.

350. Fernandez-Miranda J, Frias-Ortiz DF, Diaz-Fernandez S, Rubio-Rodriguez L. Bipolar type I and substance use disorders: safety, tolerability and effectiveness of asenapine adjunctive treatment. *Eur Neuropsychopharm.* 2017;27:S816-S817.

351. Cuomo I, Kotzalidis GD, De Persis S, Perrini F, Amici E, De Filippis S. Aripiprazole LAI vs. paliperidone LAI in inpatients with psychosis comorbid with substance use disorder: clinical and QoL effects at 1-year follow-up. *Eur Neuropsychopharm.* 2017;27:S952-S953.

352. Anand R, Hartman R, Graham S, Forrest E, Faravelli L. Evenamide, a Putative Antipsychotic, Targets Abnormal Electrical Activity and Glutamatergic Abnormalities to Improve Psychotic Symptoms in Patients with Schizophrenia: Results from a Phase Ii, Placebo-Controlled Trial. *Schizophrenia Bull.* 2017;43:S13-S14.

353. Mofsen R, Cheng CT, Nemeth G, Barabassy A, Earley W. Efficacy of Cariprazine in Patients with Schizophrenia Based on Stage of Illness. *Schizophrenia Bull.* 2017;43:S55-S55.

354. Zrinzo L, Tyagi H, Foltynie T, et al. A randomised controlled trial of Deep Brain Stimulation in Severe Refractory Obsessive Compulsive Disorder. Stereotact Funct Neurosurg 2017.

355. Voort JLV, Morgan RJ, Kung S, et al. Continuation phase intravenous ketamine in adults with treatment resistant depression. *J Affect Disorders.* 2016;206:300-304.

356. Schlaepfer T, Bewernick B, Kayser S, Gippert S, Switala C, Coenen V. Deep Brain Stimulation to the Medial Forebrain Bundle in Treatment-Resistant Depression-A Placebo Controlled Clinical Study. Paper presented at: NEUROPSYCHOPHARMACOLOGY2016.

357. Sturup AE, Jensen H, Dolmer S, et al. Discontinuation Versus Maintenance Therapy with Antipsychotic Medication in Schizophrenia - Tailor: A Randomized Controlled Trial. *Early Interv Psychia.* 2018;12:141-141.

358. Jauhar S, Veronese M, Nour MM, et al. Determinants of treatment response in first-episode psychosis: an F-18-DOPA PET study. *Mol Psychiatr.* 2019;24(10):1502-1512.

359. Zarranz I, Lopez MDP. Quality of life related to physical function and health: 2-years follow-up of outpatients with mental disorder treated with paliperidone palmitate. *Eur Neuropsychopharm.* 2016;26:S557-S558.

360. Waite S, Galvez V, Li A, et al. Patient-Rated Quality of Life after Electroconvulsive Therapy: A Multisite Naturalistic Australian Study. *Aust Nz J Psychiat.* 2016;50:34-34.

361. Gavrilova S, Kolykhalov I, Ponomareva E, Selezneva N. A clinical trial of the use of agomelatine for the treatment of depression in elderly patients in out-patient conditions. *Neuroscience and Behavioral Physiology.* 2016;46(3):365-370.

362. Kaltsatou A, Kouidi E, Fountoulakis K, et al. Effects of exercise training with traditional dancing on functional capacity and quality of life in patients with schizophrenia: a randomized controlled study. *Clin Rehabil.* 2015;29(9):882-891.

363. Philipp M, Kohnen R, Hiller KO. Hypericum extract versus imipramine or placebo in patients with moderate depression: randomised multicentre study of treatment for eight weeks. *BMJ.* 1999;319(7224):1534-1538.

364. Fortner MR, Brown K, Varia IM, Gersing KR, O'Connor C, Doraiswamy PM. Effect of Bupropion SR on the Quality of Life of Elderly Depressed Patients With Comorbid Medical Disorders. *Prim Care Companion J Clin Psychiatry.* 1999;1(6):174-179.

365. Gleason OC, Yates WR, Isbell MD, Philipsen MA. An open-label trial of citalopram for major depression in patients with hepatitis C. *J Clin Psychiatry.* 2002;63(3):194-198.

366. Ceroni GB, Rucci P, Berardi D, Ceroni FB, Katon W. Case review vs. usual care in primary care patients with depression: a pilot study. *Gen Hosp Psychiatry.* 2002;24(2):71-80.

367. Miner CM, Brown EB, Gonzales JS, Munir R. Switching patients from daily citalopram, paroxetine, or sertraline to once-weekly fluoxetine in the maintenance of response for depression. *J Clin Psychiatry.* 2002;63(3):232-240.

368. Marangell LB, Rush AJ, George MS, et al. Vagus nerve stimulation (VNS) for major depressive episodes: one year outcomes. *Biol Psychiatry.* 2002;51(4):280-287.

369. Gask L, Dowrick C, Dixon C, et al. A pragmatic cluster randomized controlled trial of an educational intervention for GPs in the assessment and management of depression. *Psychol Med.* 2004;34(1):63-72.

370. Trick L, Stanley N, Rigney U, Hindmarch I. A double-blind, randomized, 26-week study comparing the cognitive and psychomotor effects and efficacy of 75 mg (37.5 mg b.i.d.) venlafaxine and 75 mg (25 mg mane, 50 mg nocte) dothiepin in elderly patients with moderate major depression being treated in general practice. *J Psychopharmacol.* 2004;18(2):205-214.

371. Gulseren L, Gulseren S, Hekimsoy Z, Mete L. Comparison of fluoxetine and paroxetine in type II diabetes mellitus patients. *Arch Med Res.* 2005;36(2):159-165.

372. Cole MG, McCusker J, Elie M, Dendukuri N, Latimer E, Belzile E. Systematic detection and multidisciplinary care of depression in older medical inpatients: a randomized trial. *CMAJ.* 2006;174(1):38-44.

373. Wise TN, Wiltse CG, Iosifescu DV, Sheridan M, Xu JY, Raskin J. The safety and tolerability of duloxetine in depressed elderly patients with and without medical comorbidity. *Int J Clin Pract.* 2007;61(8):1283-1293.

374. Rollman BL, Belnap BH, LeMenager MS, et al. Telephone-delivered collaborative care for treating post-CABG depression: a randomized controlled trial. *JAMA.* 2009;302(19):2095-2103.

375. Kendrick T, Chatwin J, Dowrick C, et al. Randomised controlled trial to determine the clinical effectiveness and cost-effectiveness of selective serotonin reuptake inhibitors plus supportive care, versus supportive care alone, for mild to moderate depression with somatic symptoms in primary care: the THREAD (THREshold for AntiDepressant response) study. *Health Technol Assess.* 2009;13(22):iii-iv, ix-xi, 1-159.

376. Brothers BM, Yang HC, Strunk DR, Andersen BL. Cancer patients with major depressive disorder: testing a biobehavioral/cognitive behavior intervention. *J Consult Clin Psychol.* 2011;79(2):253-260.

377. Han C-h, Chung M-i, Bose S, et al. The effect of Gami Guibitang (GGBT), a medicinal herbal formulation, on moderate depression. *European Journal of Integrative Medicine.* 2012;4(4):e400-e407.

378. Bewernick BH, Kayser S, Sturm V, Schlaepfer TE. Long-term effects of nucleus accumbens deep brain stimulation in treatment-resistant depression: evidence for sustained efficacy. *Neuropsychopharmacology.* 2012;37(9):1975-1985.

379. Cao Y, Li W, Shen J, Malison RT, Zhang Y, Luo X. Health-related quality of life and symptom severity in Chinese patients with major depressive disorder. *Asia Pac Psychiatry.* 2013;5(4):276-283.

380. Zhang B, Xuefan D, Weihong L, et al. Effect of group cognitive-behavioral therapy on the quality of life and social functioning of patients with mild depression. *Shanghai archives of psychiatry.* 2016;28(1):18.

381. Shinmei I, Kobayashi K, Oe Y, et al. Cognitive behavioral therapy for depression in Japanese Parkinson's disease patients: a pilot study. *Neuropsychiatr Dis Treat.* 2016;12:1319-1331.

382. Huang CJ, Huang YH, Lin CH. Factors Related to the Changes in Quality of Life for Patients With Depression After an Acute Course of Electroconvulsive Therapy. *J ECT.* 2017;33(2):126-133.

383. Yang WC, Lin CH, Wang FC, Lu MJ. Factors related to the improvement in quality of life for depressed inpatients treated with fluoxetine. *BMC Psychiatry.* 2017;17(1):309.

384. Jiang W, Whellan DJ, Adams KF, et al. Long-Chain Omega-3 Fatty Acid Supplements in Depressed Heart Failure Patients: Results of the OCEAN Trial. *JACC Heart Fail.* 2018;6(10):833-843.

385. Trottier-Duclos F, Desbeaumes Jodoin V, Fournier-Gosselin MP, et al. A 6-Year Follow-up Study of Vagus Nerve Stimulation Effect on Quality of Life in Treatment-Resistant Depression: A Pilot Study. *J ECT.* 2018;34(4):e58-e60.

386. Deslandes AC, Moraes H, Alves H, et al. Effect of aerobic training on EEG alpha asymmetry and depressive symptoms in the elderly: a 1-year follow-up study. *Braz J Med Biol Res.* 2010;43(6):585-592.

387. Revicki DA, Simon GE, Chan K, Katon W, Heiligenstein J. Depression, health-related quality of life, and medical cost outcomes of receiving recommended levels of antidepressant treatment. *J Fam Pract.* 1998;47(6):446-452.

388. Thompson C, Peveler RC, Stephenson D, McKendrick J. Compliance with antidepressant medication in the treatment of major depressive disorder in primary care: a randomized comparison of fluoxetine and a tricyclic antidepressant. *Am J Psychiatry.* 2000;157(3):338-343.
